# Halogen‐Driven Ion Transport Homogenization in 3D Hierarchical MOF for Ultrastable Solid‐State Lithium Metal Batteries

**DOI:** 10.1002/anie.202511822

**Published:** 2025-07-20

**Authors:** Xingxing Zhang, Hongli Chen, Qingmei Su, Xinglong Deng, Dequn Zhao, Weihao Shi, Liming Wang, Jinqi Chen, Fan Xi, Zeming He, Ping Yu, Guoxiu Wang, Wenhuan Huang

**Affiliations:** ^1^ Xi'an Key Laboratory of Advanced photo‐electronics Materials and Energy Conversion Device, Technological Institute of Materials & Energy Science (TIMES) Xijing University Xi'an 710123 China; ^2^ Centre for Clean Energy Technology, School of Mathematical and Physical Science, Faculty of Science University of Technology Sydney Broadway New South Wales 2007 Australia; ^3^ Materials Institute of Atomic and Molecular Science, School of Physics & Information Science Shaanxi University of Science and Technology Xi'an 710021 P.R. China; ^4^ Key Laboratory of Chemical Additives for China National Light Industry, College of Chemistry and Chemical Engineering Shaanxi University of Science and Technology Xi'an 710021 P.R. China; ^5^ Flexible Energy storage and Interfacial Chemistry Key Laboratory of Shaanxi University, College of Chemistry and Chemical Engineering Shaanxi University of Science and Technology Xi'an 710021 P. R. China

**Keywords:** Hierarchical ion‐transport network, Lithium halides, MOF confinement, Solid‐state electrolyte

## Abstract

Solid‐state lithium metal batteries (SSLMBs) are hindered by limited ionic conductivity, heterogeneous lithium flux and interfacial instability of solid‐state electrolytes. Herein, we report a hierarchical ion‐transport network formed by confining lithium halides (LiX, X═Cl, Br, I) within the mesoporous cages of MIL‐100(Al), synergistically integrated with a PVDF‐HFP polymer matrix. The 3D interconnected pores (0.5–1 nm) of MIL‐100(Al) not only spatially confine anions via size‐selective sieving but also enable continuous Li⁺ transport through tunable host–guest interactions between the Lewis‐acidic metal nodes and lithium halides. Among these, the LiI‐embedded composite (E‐LiI) exhibits a high Li⁺ transference number (0.88 at 25 °C) and favorable interfacial kinetics, attributed to strong anion coordination and homogeneous Li⁺ plating. Structural characterizations confirm uniform LiX distribution within the MOF framework. In addition, density functional theory (DFT) calculations and COMSOL simulation elucidate halogen‐dependent desolvation energetics and Li^+^ transport kinetics. SSLMBs employing E‐LiI electrolytes demonstrate exceptional cycling stability (capacity retention ∼100% after 600 cycles at 2C) with high‐voltage cathodes and wide‐temperature adaptability. This work advances the rational design of multi‐scale ion‐conductive frameworks and the pivotal role of lithium halide in regulating Li deposition kinetics, offering a transformative strategy for high‐energy‐density solid‐state battery systems.

## Graphical abstract

The hierarchical pore channels of MIL‐100 confine LiI to form a conductive ionic network, while its triple‐level cross‐linked multi‐stage porous restricts TFSI⁻ anion mobility, enabling rapid Li⁺ transport and uniform deposition by optimizing ion flux and suppressing space charge effects.

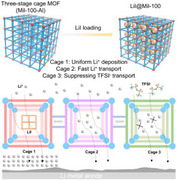

## Introduction

With the increasing demand for high‐safety and high‐energy‐density energy storage devices in new energy vehicles and portable electronics, solid‐state lithium metal batteries (SSLMBs) emerge as a pivotal breakthrough candidate for next‐generation battery systems. This is attributed to their theoretical energy density exceeding 500 Wh kg⁻^1^ (approximately twice that of conventional lithium‐ion batteries) and their ability to completely eliminate safety hazards associated with flammable or leaking liquid electrolytes.^[^ ^1^, ^2^, ^3^ ^]^ However, the core challenge lies in developing solid‐state electrolyte materials that synergistically combine high ionic conductivity, superior interfacial compatibility, and dendrite‐suppressing capabilities. While composite strategies incorporating inorganic fast‐ion conductors (e.g., ceramics, sulfides, oxides, etc.) enhance lithium transport via interfacial percolation, intrinsic limitations persist.^[^ ^4^, ^5^, ^6^, ^7^ ^]^ Young's modulus mismatch between rigid inorganic fillers and soft matrices exacerbates interfacial stress and space charge accumulation, fostering lithium dendrite nucleation under high current densities (>0.5 mA cm⁻^2^). Moreover, inert filler surfaces lack dynamic ion‐regulation capabilities, failing to balance lithium flux or suppress anion polarization, which critically undermines both ionic transport homogeneity and cycling stability.^[^ ^8^, ^9^, ^10^ ^]^ These deficiencies demand a fundamental re‐evaluation of filler design principles for advanced composite electrolytes.

To overcome the aforementioned limitations, metal‐organic frameworks (MOFs), featuring designable topological architectures (with tunable pore sizes ranging from 0.5 to 3 nm) and high specific surface areas (>1000 m^2^ g^−1^), demonstrate significant potential in regulating ion transport and modulating lithium deposition behavior when composited with PVDF‐HFP‐based polymer electrolytes.^[^ ^11^, ^12^ ^]^ This establishes MOFs as an advanced frontier in functionalized solid‐state electrolyte composites. The hierarchical pore channels of MOFs restrict anion migration through size‐sieving mechanisms. Furthermore, the inherent Lewis acidity of MOFs enables interactions with polymer chains by accepting lone electron pairs from polar groups, thereby weakening polymer crystallinity and enhancing ion transport rates.^[^ ^13^, ^14^ ^]^ However, the intrinsically insulating nature of MOFs in terms of ionic conductivity remains insufficient to fulfill the requirements for high‐performance electrolytes.^[^ ^15^ ^]^ Consequently, further research and innovation remain imperative to address these challenges in practical applications.

The confinement effect of MOF pore channels, which synergistically enhances ion transport by hosting guest ionic conductors, represents the most promising approach to addressing this challenge. However, existing studies primarily focus on the incorporation of single‐component lithium salts or ionic liquids (e.g., LiTFSI, LiClO_4_, [EMIM] [TFSI], etc.), with limited systematic investigation into the interplay between MOF pores and guest ion conductors.^[^ ^16^, ^17^, ^18^, ^19^ ^]^ Additionally, decomposition byproducts (e.g., Li_2_O, Li_2_S) from conventional guest ion conductors tend to form high‐impedance solid electrolyte interphases (SEI) on lithium metal surfaces, resulting in sluggish interfacial kinetics.^[^ ^20^, ^21^ ^]^ Consequently, there is an urgent need to explore synergistic mechanisms between novel lithium salt systems and MOF confinement effects to overcome current limitations. Lithium halides (LiX, X═F, Cl, Br and I), as emerging ionic conductors, exhibit superior ionic transport properties. However, their direct integration with polymers often deteriorates interfacial contact due to high crystallinity.^[^ ^22^, ^23^, ^24^ ^]^ This necessitates the development of composite strategies with porous MOFs to achieve complementary material performance. Although LiF demonstrates exceptional performance as both an ionic conductor and an SEI component, its poor solubility in most organic solvents hinders effective confinement within MOF pores to form high‐quality composite ionic conductors.^[^ ^25^ ^]^ Therefore, among candidate lithium halides, highly soluble LiCl, LiBr, and LiI emerge as promising guest species for MOF confinement, enabling the fabrication of advanced hybrid ionic conductors.

In this work, MIL‐100(Al) with a three‐dimensional hierarchical pore architecture (interconnected 0.5–0.9 nm micropores) is utilized to spatially confine and host LiCl, LiBr, and LiI, forming three distinct ionic conductors. The three‐dimensional hierarchical pore structure (with three distinct porous architectures functionally designed to simultaneously achieve lithium halide guest accommodation, Li⁺ ion conduction, and TFSI⁻ anion migration confinement) achieves topology optimization of an ion transport “trunk‐branch” network. Moreover, MIL‐100(Al) exhibits exceptional physicochemical stability, ensuring structural integrity during lithium halide loading and prolonged battery cycling. Transmission electron microscopy (TEM) and energy‐dispersive spectroscopy (EDS) confirm the successful encapsulation of lithium halide nanoparticles within MIL‐100(Al) cages, further validated by powder X‐ray diffraction (PXRD) and Brunauer–Emmett–Teller (BET) analyses. Subsequently, these MOF/LiX composites are incorporated into a PVDF‐HFP matrix to fabricate hybrid solid‐state electrolytes (denoted as E‐LiX, X═Cl, Br, I). Theoretical calculations and COMSOL simulation studies systematically reveal the influence of different lithium halides as cooperative ionic conductors on Li⁺ transport and deposition behavior, establishing a structure‐property relationship between chemical attributes and electrochemical performance. Electrochemical experiments elucidate the distinct roles of lithium halides, highlighting that the E‐LiI electrolyte achieves both high ionic migration rates (5.2 × 10^−4^ S cm^−1^) and uniform Li⁺ deposition at the Li anode interface. The assembled LFP||Li cells deliver an initial specific capacity of 115.6 mAh g^−1^ at 2C, with a capacity retention of ∼100% after 600 cycles, alongside remarkable cycling stability over a wide temperature range. Notably, E‐LiI‐based pouch cells paired with high‐voltage cathode materials demonstrate stable operation, showcasing significant potential for high‐energy‐density battery applications. This study establishes a novel paradigm for the rational design of multi‐scale composite ion transport channels and elucidates the unique mechanistic role of halide chemistry in regulating ion transport and deposition kinetics in solid‐state batteries.

## Results and Discussion

**Figure 1:**
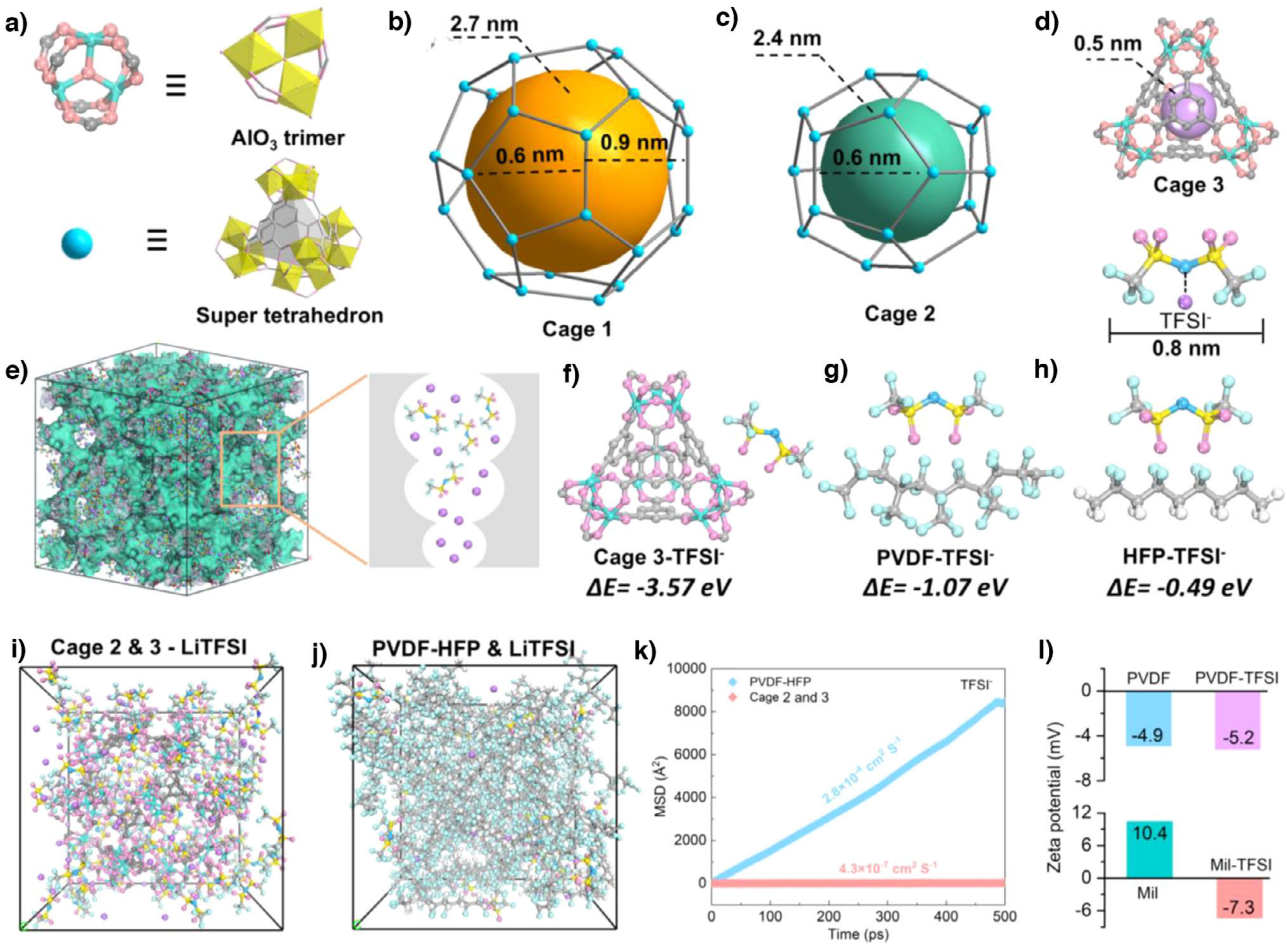
Structure schematic of Mil‐100(Al) and mechanism of Li^+^ transport. a) The Al_3_(μ_3_‐O)X_3_(O_2_CR)_6_ clusters and the supertetraedra formed with trimesate linkers. b) Cage 1 of Mil‐100, c) Cage 2 of Mil‐100, d) Cage 3 of Mil‐100 and size of TFSI^−^. e) Schematic of Mil‐100(Al) for TFSI^−^ confined and Li^+^ transport. (f‐h) Binding energies of Cage 3 and PVDF‐HFP for Li^+^. (i,j) Snapshots of the MD simulation of Cage 2, Cage 3, and PVDF‐HFP with LiTFSI. (k) Mean‐squared displacement (MSD) of TFSI^−^ in Cage 2&Cage 3 and PVDF‐HFP. l) Zeta potential value of PVDF and Mil‐100 with TFSI^−^.

Mil‐100 (Al) was synthesized by hydrothermal method from a mixture of trimesic acid, aluminum nitrate, nine hydrate, and water (Figures 1a and S1). MIL‐100(Al) framework features two mesoporous cages (Cage 1 and Cage 2) and one microporous cage (Cage 3). The two mesoporous cages measure 2.7 and 2.4 nm in size, respectively. Cage 1 is composed of four hexagonal apertures with pore diameters of 0.9 nm and 12 pentagonal apertures of 0.6 nm (Figure 1b). Cage 2 contains 12 pentagonal apertures with 0.6 nm diameters (Figure 1c). Cage 3 contains of a tetrahedron‐like microporous structure exhibiting a cage dimension of 0.5 nm (Figure 1d).^[^ ^26^ ^]^ Notably, the TFSI^−^ size of 0.8 nm (Figure 1d), indicating restricted transport within the confines of Cage 2 and Cage 3.^[^ ^27^ ^]^ Owing to the presence of larger mesoporous cages capable of accommodating guest species, combined with its smaller micropores that effectively confine the migration of bulky guests, MIL‐100(Al) emerges as a promising host material for the development of confined ionic conductors (Figure 1e).

Density functional theory (DFT) calculations and molecular dynamics (MD) simulations were employed to investigate the confinement capability of MIL‐100 (Al) and PVDF‐HFP toward TFSI⁻ anions. The binding energy analysis revealed that Cage 3 of MIL‐100 exhibited a significantly higher binding energy (‐3.57 eV, Figure 1f) with TFSI⁻ compared to the two segments of PVDF‐HFP (1.07 and 0.49 eV, Figure 1g,h), demonstrating superior confinement of TFSI⁻ within Cage 3. Furthermore, solventization models involving LiTFSI with Cage 2, Cage 3, and PVDF‐HFP were constructed for MD simulations to evaluate the diffusion behavior of TFSI⁻ (Figure 1i,j). The mean squared displacement (MSD) analysis indicated that the diffusion coefficient of TFSI^−^ in PVDF‐HFP (2.8 × 10^−4^ cm^2^ S^−1^) at 25 °C substantially exceeded those within Cage 2 & Cage 3 (4.3 × 10^−7^ cm^2^ S^−1^) of MIL‐100 (Figure 1k). This disparity highlights the restricted TFSI^−^ transport in MIL‐100 cavities due to pore confinement effects. Zeta potential analysis further elucidated the enhanced confinement mechanism. The initial zeta potentials of PVDF and MIL‐100 (Al) were −4.9 and + 10.4 mV, respectively, which shifted to −5.2 and ‐7.3 mV after TFSI^⁻^ incorporation (Figure 1l). This reversal in MIL‐100 surface charge confirms strong electrostatic interactions between negatively charged TFSI⁻ and the positively charged framework, facilitating preferential anion adsorption. Collectively, these findings establish that MIL‐100 (Al) effectively immobilizes TFSI⁻ through dual confinement mechanisms, thereby eliminating anion‐induced barriers and promoting unobstructed Li⁺ transport within the porous channels.

**Figure 2:**
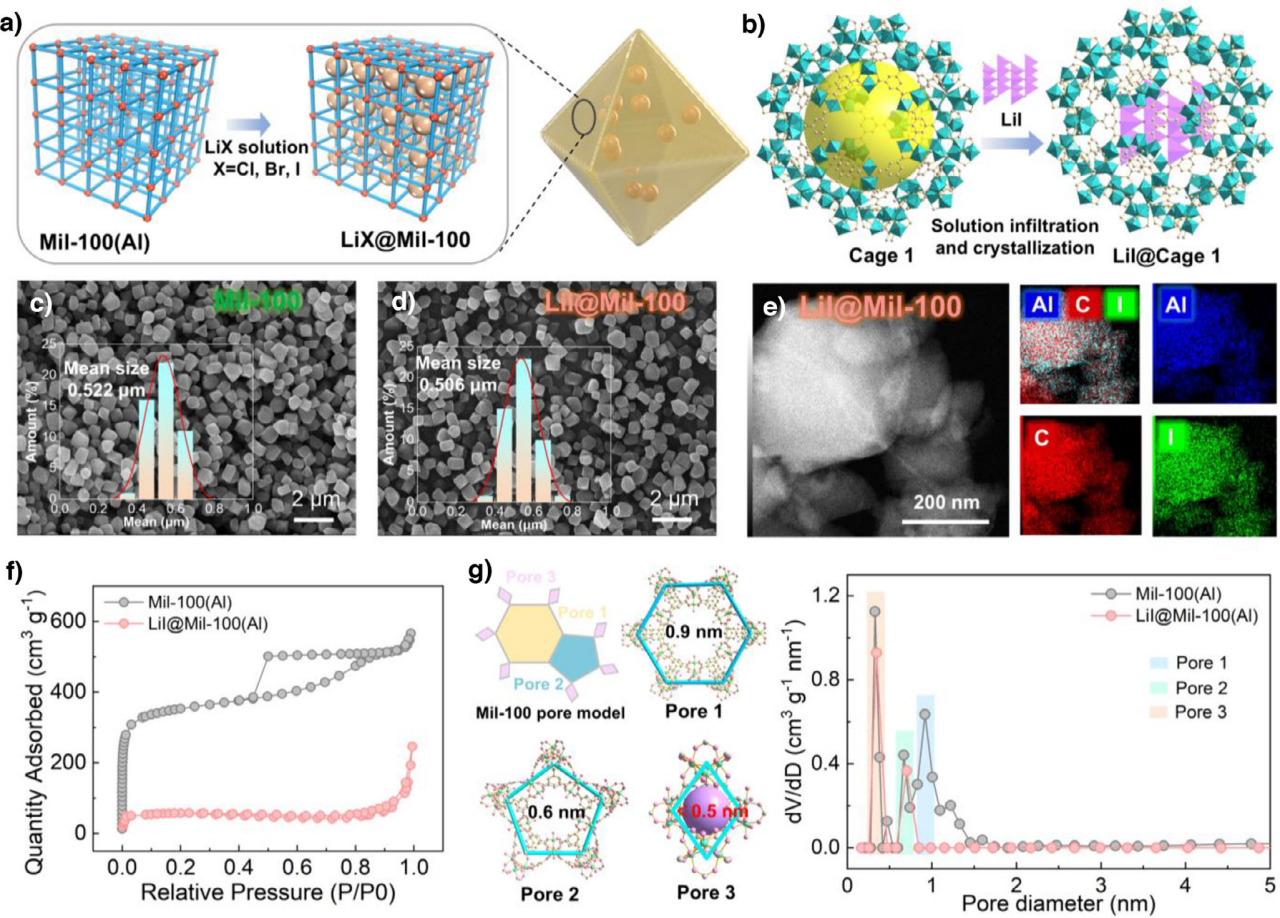
Preparation of LiX@Mil‐100 and characterization. a), b) Schematic diagram of the preparation of LiX@Mil‐100. c), d) SEM images of Mil‐100(Al) and LiI@Mil‐100 (embedded particle size distributions). e) TEM image and EDS mapping of LiI@Mil‐100. f), g) N_2_ sorption isotherms and pore size of Mil‐100(Al) and LiI@Mil‐100 at 77K and 1 atm.

To achieve lithium halide (LiX, X═Cl, Br, or I) ion conductors confined within MIL‐100, an impregnation method, namely the ship‐in‐bottle strategy, was employed to synthesize the composite, ensuring uniform nucleation of LiX nanocrystallines within the MIL‐100 cages. Specifically, pre‐synthesized MIL‐100(Al) powder was soaked in a methanolic solution of LiX under vigorous stirring for 24 h to allow complete infiltration of LiX into the MOF cavities. The resultant material was then centrifuged and thoroughly washed with methanol to remove surface‐adsorbed LiX, followed by drying to yield the final LiX@MIL‐100 composite (Figure 2a,b, and S2). As shown in Figure S3, the PXRD patterns of LiX@MIL‐100 closely match the simulated peaks of pristine MIL‐100(Al), confirming the structural integrity of the MOF framework after LiX incorporation. Notably, no diffraction peaks of LiX were detected for LiX@Mil‐100 prepared by the co‐precipitation method, whereas the physical mixing of LiI and Mil‐100 resulted in the appearance of the characteristic LiI peaks in Mil‐100 (Figure S3, 4), which indicates that LiX within successfully confined within the cages and no crystal aggregation has occurred. Furthermore, SEM images of both MIL‐100(Al) and LiX@MIL‐100 reveal well‐preserved regular octahedral morphologies with no significant difference in particle size (Figure 2c, d, and S5), demonstrating that the impregnation process does not compromise the crystalline architecture of the MIL‐100 matrix. X‐ray photoelectron spectroscopy (XPS) was employed to investigate the potential formation of new chemical bonds (such as Al─X or Li─O─Al bonds) in the LiX@MIL‐100 composite. Comparative analysis revealed no significant changes in the binding energies of the Al 2p and O 1s spectra between the LiX@MIL‐100 composite and pristine MIL‐100 (Figure S6a,b). Furthermore, the Li 1s spectra of the three composite samples exclusively exhibit distinct Li‐X characteristic peaks (Figure S6c). This indicates that no strong chemical interactions or electron transfer occurred between LiX and the MIL‐100 host framework after loading. Consequently, no new chemical bonds were formed; LiX exists only by physical confinement within the structure. Furthermore, elemental distribution maps acquired using TEM‐energy dispersive spectroscopy (TEM‐EDS) confirmed the homogeneous dispersion of Cl, Br, and I species throughout the MIL‐100 matrix across three distinct LiX@MIL‐100 composites (Figures 2e and S7–S9). These collective evidences substantiate that LiX is homogeneously distributed within the host framework while maintaining its structural stability. The actual Li loadings in LiCl@Mil‐100, LiBr@Mil‐100, and LiI@Mil‐100 composites were quantified by ICP‐OES as 11.52 wt%, 10.58 wt%, and 11.02 wt%, respectively (Table S1). These values fall within a narrow range, ensuring that comparisons of ionic conductivity and Li⁺ transport properties across samples are conducted on an equal basis. To verify the successful encapsulation of LiX nanoparticles within the MIL‐100 cages, the specific surface areas and pore sizes of various samples were systematically investigated by N_2_ adsorption‐desorption analysis (Thoroughly washed samples underwent solvent exchange with MeOH at least five times, and dried in a pre‐heated 85 °C oven for 30 min. Resulting powder was then activated for BET measurement at 120 °C for 2 h). The pristine MIL‐100(Al) exhibited a BET surface area of 1611 m^2^ g^−1^ and pore dimensions consistent with literature values (Figure 2f).^[^ ^28^ ^]^ Notably, LiX@MIL‐100 composites demonstrated substantially reduced BET surface areas and narrowed pore sizes, attributable to the occupation of MIL‐100 cavities by highly dispersed LiX nanocrystals (Figure 2f). To further determine the specific cages occupied by LiI within Mil‐100(Al), we designated the 0.9 nm pores as pore 1, the 0.6 nm pores as pore 2, and the channels within cage 3 as pore 3 (less than 0.5 nm). As shown in Figure 2g, the pore size distribution confirms that Mil‐100 possesses a hierarchical pore structure with distributions corresponding to these three pore sizes. Upon loading with LiI nanocrystals, the characteristic peak for pore 1 vanished in the loaded Mil‐100, while no significant changes were observed for pores 2 and 3. This indicates that LiI nanocrystals are predominantly distributed within cage 1 of Mil‐100(Al). These results affirm the successful nucleation of LiX nanocrystals within the cage‐structured MIL‐100 host, ultimately yielding Li@MIL‐100 composite ion conductors with well‐defined architectures. Energy barrier calculations reveal that Li^+^ migration within cage 2 (0.105 eV) and cage 3 (0.088 eV) exhibits lower energy barriers compared to cage 1 (0.125 eV) in MIL‐100 (Figure S10). This further highlights the significant potential of synergistic inter‐cage interactions in promoting Li^+^ mobility.

To synthesize LiX@Mil‐100 ionic conductor‐doped composite solid‐state electrolyte membranes, PVDF‐HFP‐based composite solid electrolytes embedded with LiX@Mil‐100 were prepared via a solution casting method, as illustrated in Figures S11, S12. Specifically, PVDF‐HFP, LiTFSI, and LiX@Mil‐100 were homogeneously dissolved in N‐Methyl−2‐pyrrolidone (NMP) solvent and cast into polytetrafluoroethylene molds. After drying, three composite solid electrolytes (denoted as E‐LiCl, E‐LiBr and E‐LiI) were obtained. Surface and cross‐sectional SEM images revealed that the LiX@Mil‐100 ionic conductors were uniformly dispersed within the PVDF‐HFP matrix, forming dense solid electrolyte membranes with a thickness of ∼50 µm (Figure S13). Furthermore, three batches of electrolyte membranes were fabricated with an average thickness of 50 ±1.5 µm. The thickness for all batches closely matched the target value of 50 µm, exhibiting a narrow error range (Figures S14, S15). This confirms the high reproducibility of the electrolyte membrane fabrication process. XRD patterns confirmed the presence of characteristic peaks corresponding to Mil‐100 (Al) in all electrolyte membranes, and no additional lithium halide characteristic peaks were found, indicating that the crystalline structure of LiX@Mil‐100 remained intact during the fabrication process (Figure S16). Furthermore, compared to pure PVDF‐HFP membranes, the composite membranes doped with LiX@Mil‐100 exhibited attenuated PVDF‐HFP diffraction peaks, demonstrating reduced crystallinity of PVDF‐HFP, which is beneficial for enhancing Li^+^ transport along the polymeric chains. Subsequently, the physical properties of the membranes were systematically evaluated to explore their practical potential under diverse conditions. Flexibility tests revealed that the E‐LiI electrolyte could return to its original morphology after physical folding, rolling, and wrapping while maintaining intrinsic flexibility (Figure S17). Moreover, incorporating LiX@Mil‐100 into PVDF‐HFP significantly improved the thermal stability of the E‐LiX electrolytes, achieving dimensional stability up to 100 °C, which far exceeds that of commercial polypropylene (PP) separators (∼80 °C thermal shrinkage, Figure S18). Thermogravimetric analysis (TGA) further reveals that the E‐LiI electrolyte exhibits superior thermal stability, demonstrating no decomposition at temperatures up to 160 °C, with significant decomposition onset only occurring above 350 °C (Figure S19). The combined advantages of exceptional flexibility and robust thermal stability endow the E‐LiX electrolytes with promising commercial application potential.

**Figure 3:**
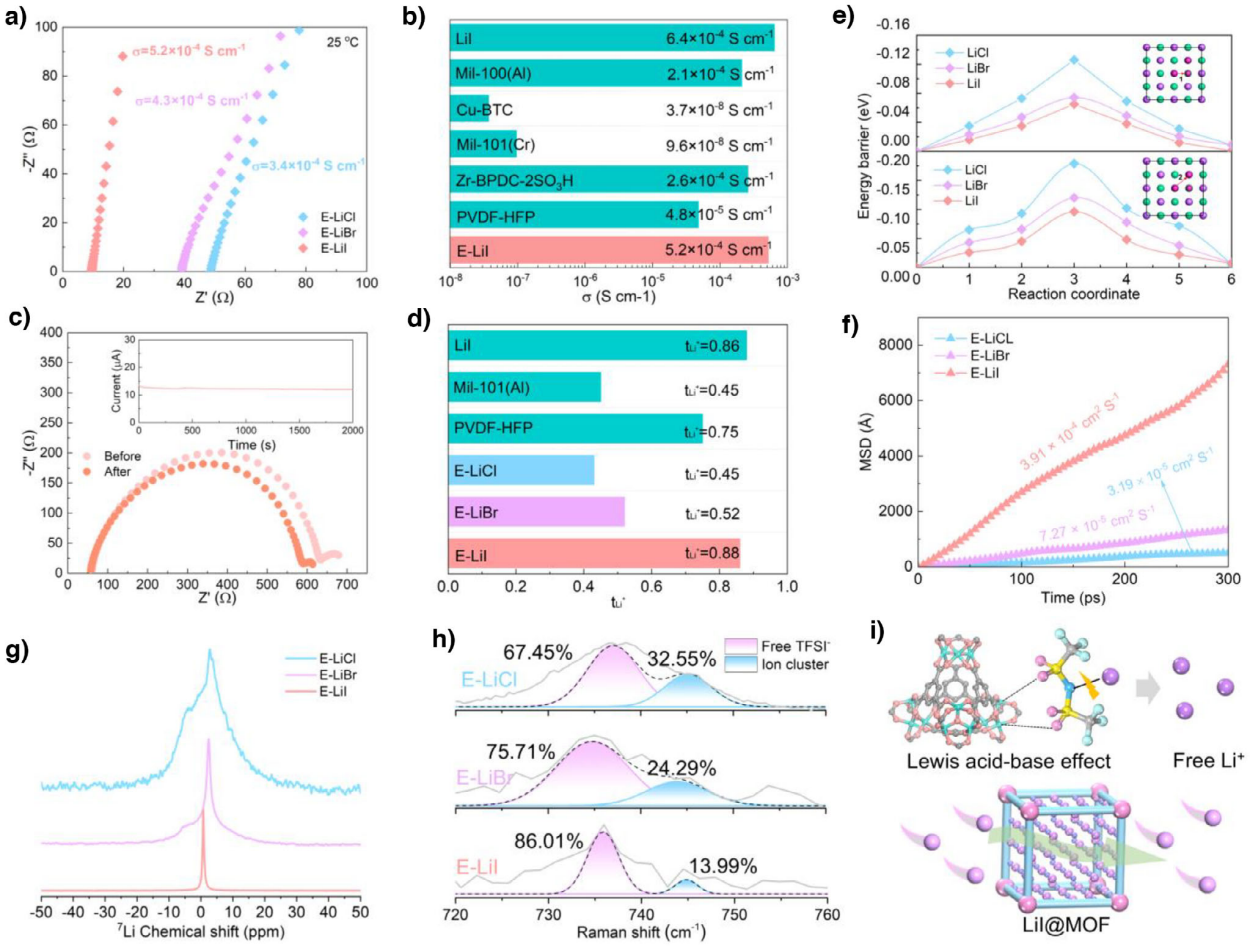
Preparation of E‐LiX Electrolyte Membranes and Li^+^ transport behavior. a) EIS characterization of E‐LiCl, E‐LiBr and E‐LiI at 25 °C. b) Comparison of the Li^+^ conductivity of E‐LiI with other relevant ion conductors. c) EIS spectra before and after polarization of Li|E‐LiI|Li cell, the insets show the corresponding polarization curve. d) Comparison of the t_Li_^+^ transference number of different relevant electrolytes. e) Diffusion barriers for Li^+^ on LiCl, LiBr and LiI. f) Mean‐squared displacement (MSD) of Li/LiCl, Li/LiBr and Li/LiI at 298K. g) 1D ^7^Li nuclear magnetic resonance (NMR) of E‐LiCl, E‐LiBr and E‐LiI. h) Raman spectra of the E‐LiCl, E‐LiBr, E‐LiI and the corresponding quantification results of the TFSI^−^ anion states. i) Schematic of LiTFSI dissociation and Li^+^ transport on LiI@Mil‐100 frameworks.

The influence of different electrolyte membranes on Li^+^ transport was systematically evaluated through measurements of ionic conductivity and lithium‐ion transference number. As shown in Figure 3a, the E‐LiI electrolyte demonstrated the lowest charge transfer resistance (R_ct_) of 9.76 Ω at 25 °C, corresponding to the highest ionic conductivity of 5.2 × 10^−4^ S cm^−1^, which outperformed E‐LiCl (3.4 × 10^−4^ S cm^−1^) and E‐LiBr (4.3 × 10^−4^ S cm^−1^). Notably, MOF‐based electrolytes lacking either PVDF‐HFP matrix or LiI additive exhibited inferior room‐temperature ionic conductivity compared to E‐LiI (Figure 3b).^[^ ^28^, ^29^, ^30^, ^31^ ^]^ The intrinsic ionic conductivity of LiI (6.4 × 10^−4^ S cm^−1^) further confirmed its capability to facilitate Li^+^ transport. The activation energy (Ea) for Li^+^ migration, calculated from temperature‐dependent Rct variations, revealed superior transport kinetics in E‐LiI (E_a_ = 0.53 eV) relative to E‐LiCl (0.98 eV) and E‐LiBr (0.86 eV, Figure S20). Considering the concurrent migration of cations and anions in solid electrolytes, Li^+^ transference numbers (t_Li+_) were tested to assess Li^+^ transport efficiency. As shown in Figure 3c,d, and S21, E‐LiCl and E‐LiBr exhibited t_Li+_ values of 0.45 and 0.52, respectively. In striking contrast, E‐LiI achieved a remarkable t_Li+_ of 0.88, surpassing those of pristine LiI (0.86), MIL‐100(Al) (0.45), and PVDF‐HFP matrix (0.75). These results underscore the effectiveness of E‐LiI in suppressing TFSI^−^ anion mobility while enhancing Li^+^ conduction. The electrochemical stability window was investigated through linear sweep voltammetry (LSV) to evaluate compatibility with high‐voltage cathodes. Benefiting from the synergistic interaction between MIL‐100 Lewis acid sites and PVDF‐HFP chains, all three electrolytes exhibited a voltage window of approximately 4.8 V, representing significant improvement over the literature‐reported 4.5 V for pure PVDF‐HFP (Figure S22). This enhancement indicates the critical role of MIL‐100 integration in broadening the electrochemical stability of polymer‐based electrolytes.

Subsequently, the mechanism underlying the exceptional Li⁺ transport capability of E‐LiI was investigated. DFT calculations were conducted to determine the energy barriers for Li⁺ migration toward halogen atom sites (Cl, Br or I; Path 1) and Li top sites (Path 2), with the diffusion pathways illustrated in the inset of Figures 3e and S23 (the surface energy was calculated to determine the Li^+^ migration pathway, Table S2). Remarkably, LiI exhibited notably lower energy barriers of 0.077 eV (via Path 1 to Li top sites) and 0.075 eV (via Path 2 to I top sites) compared with those of LiCl (0.182 and 0.124 eV) and LiBr (0.128 and 0.096 eV, Figures 3e and S24), suggesting minimal resistance for Li⁺ transport across the LiI surface and enhanced charge/discharge efficiency. Moreover, the chemical interaction of Li^+^ on different LiX (X═Cl, Br or I) surfaces was investigated by calculating the adsorption energies at various adsorption sites (Li‐top site‐1, halogen‐top site‐2, hollow site, and bridge site). As shown in Figure S25, the adsorption energies of Li^+^ on LiI (‐0.11, ‐0.36, ‐0.27, and ‐0.45 eV) exhibit notably higher values compared to those of LiCl (‐0.02, ‐0.27, ‐0.13, and ‐0.26 eV) and LiBr (‐0.02, ‐0.29, ‐0.19, and ‐0.28 eV). These findings reveal that LiI demonstrates stronger participation in the competitive adsorption of Li^+^, thereby facilitating more efficient Li^+^ transport on the LiI surfaces due to its lower energy barrier characteristics. Further by MD simulations quantified Li⁺ diffusion coefficients in lithium halides. The time‐dependent mean square displacement (MSD) analysis revealed a superior Li⁺ diffusion coefficient in LiI (3.91 × 10^−4^ cm^2^ s^−1^), surpassing those of LiCl (3.19 × 10^−5^ cm^2^ s^−1^) and LiBr (7.27 × 10^−5^ cm^2^ s^−1^) by an order of magnitude (Figures 3f and S26, 27 shows the temperature fluctuation profile in the range of 5–10% during the MD simulation of the Li/LiX system, indicating that the kinetic equilibrium has been reached). The high diffusion coefficient in LiI aligns well with experimental conductivity and transference number measurements, unequivocally demonstrating the exceptional potential of LiI in facilitating rapid Li⁺ transport for advanced battery applications.

To investigate the chemical environment and mechanisms of Li^+^ transport in different electrolytes, ^7^Li solid‐state nuclear magnetic resonance (NMR) measurements were performed on various electrolytes to understand the enhanced Li^+^ transport in E‐LiI.^[^ ^32^ ^]^ As shown in Figure 3 g, broader signals were observed in the spectra of E‐LiCl and E‐LiBr, indicating stronger binding states of Li^+^ in these channels with greater resistance to transport. In contrast, the Li^+^ signal in the E‐LiI spectrum became sharper, suggesting that Li^+^ in E‐LiI is more easily released from its coordination state for enhanced mobility. Furthermore, Raman spectroscopy was employed to analyze the concentrations of free TFSI^−^ and ion clusters, thereby determining the ability of different electrolytes to dissociate coordinated Li^+^ (Figure 3 h). In the E‐LiI electrolyte, the concentrations of free TFSI^−^ and ion clusters (LiTFSI) were 86.01% and 13.99%, respectively, with the concentration of free TFSI⁻ being higher than that in E‐LiCl (67.45%) and E‐LiBr (75.71%). This indicates that the introduction of LiI@Mil‐100 into the electrolyte enhances the dissociation of LiTFSI, thereby improving Li^+^ transport. These results demonstrate that incorporating LiI@Mil‐100 into PVDF‐HFP facilitates the dissociation of Li‐TFSI and significantly enhances Li^+^ mobility in E‐LiI (Figure 3i).

**Figure 4:**
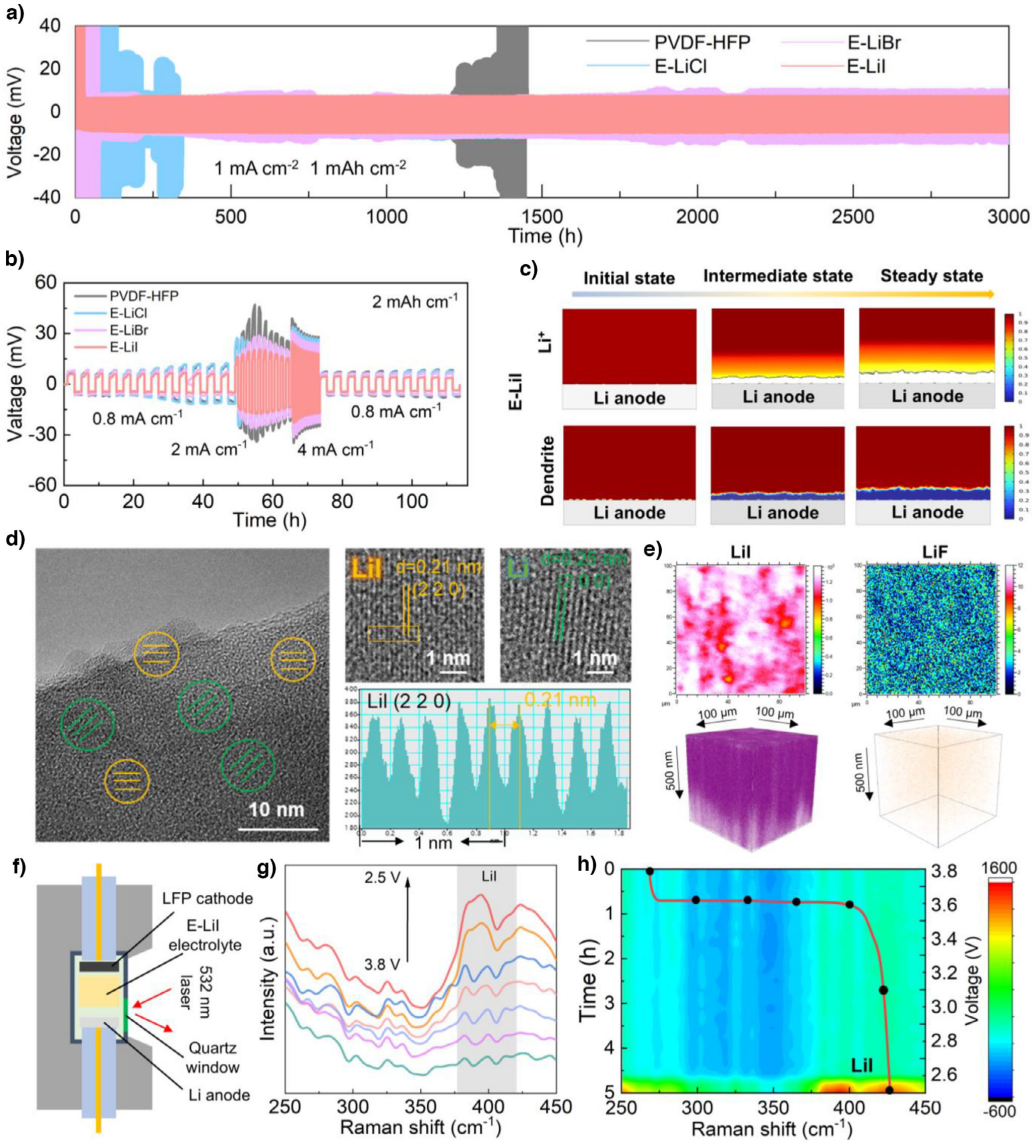
Li^+^ deposition behavior in different electrolytes. a) The Li plating/stripping performances of the Li||PVDF‐HFP||Li, Li||E‐LiCl||Li, Li||E‐LiBr||Li and Li||E‐LiI||Li symmetric cells cyclability at 1 mA cm^−2^, 1 mAh cm^−2^, and b) different current density. c) Distribution for Li^+^ concentration and Li‐deposition of the E‐LiI in different states based on COMSOL simulation analysis. d) Cryo‐TEM characterization of SEI between E‐LiI and Li anode surface. TEM image and HRTEM image, yellow: LiI; green: Li, and corresponding FFT of LiI phase. e) Spatial distribution of LiI and LiF on Li anode surface detected by TOF‐SIMS after LFP/E‐LiI/Li cycling. f) Schematic diagram of Raman test setup. g) Raman spectra of the LFP/E‐LiI/Li cell at different voltages. h) In situ Raman images of the the LFP/E‐LiI/Li cell with. The inset red curve in i) is the voltage profile of the the LFP/E‐LiI/Li cell.

To investigate the Li deposition behavior at electrode–electrolyte interface matched with different electrolytes, Li//Cu half‐cells were assembled to monitor the lithium nucleation process. Specifically, continuous lithium plating on the Cu foil surface for 5 h to evaluate the overpotential of lithium deposition on the Cu foil surface. The E‐LiI electrolyte demonstrated a lower overpotential of −44.6 mV. In contrast, E‐LiCl and E‐LiBr exhibited significantly higher overpotentials of −67.3 and ‐50.5 mV, respectively (Figure S28), attributable to their inferior Li^+^ transport kinetics. SEM characterization of post‐deposition Cu foil surfaces further revealed inhomogeneous lithium deposition morphologies in E‐LiCl and E‐LiBr systems, corroborating their elevated deposition overpotentials (Figure S29). Subsequent evaluation of Li plating/stripping behavior in Li//Li symmetric cells (1, 1 mAh cm^−2^) using PVDF‐HFP, E‐LiCl, E‐LiBr, and E‐LiI electrolytes demonstrated distinct performance variations. As shown in Figure 4a,  E‐LiI achieved a minimal polarization voltage of 10 mV with exceptional stability, while E‐LiCl and E‐LiBr displayed higher polarization voltages of 15 mV due to insufficient Li^+^ transport kinetics. Notably, pristine PVDF‐HFP electrolyte suffered from internal short‐circuiting after 1200 h cycling, underscoring the mechanical reinforcement effect of LiX@Mil‐100 nanoparticles in suppressing lithium dendrite penetration. Furthermore, even the current density was progressively increased from 0.8 to 4 mA cm^−2^ under an areal capacity of 2 mAh cm^−2^, the E‐LiI electrolyte still maintained low and stable polarization voltages compared with PVDF‐HFP, E‐LiCl and E‐LiBr electrolytes (Figure 4b and Table S3), which further corroborates that the rapid ion transport kinetics of E‐LiI effectively reduces the interfacial resistance of Li^+^ at the lithium metal surface. Post‐cycling SEM analysis unveiled compact and homogeneous Li deposition with negligible dendrite formation in E‐LiI systems (Figure S30a,b). In contrast, porous and swollen Li morphologies accompanied by extensive dendrites and dead Li were observed in E‐LiCl and E‐LiBr systems (Figure S30c–f). Step profiler further validated these findings (Figure S31), displaying smooth Li deposition in E‐LiI versus rugged and uneven surfaces in E‐LiCl and E‐LiBr configurations.

The underlying mechanism of Li^+^ deposition behavior was systematically investigated through theoretical calculations and COMSOL multiphysics simulations. Structural optimization and surface charge distribution analysis were performed on three lithium halides (LiCl, LiBr, and LiI). As shown in Figure S32, the I atoms in LiI exhibit less localized charge distribution around the surface compared to the denser charge accumulations near Cl and Br atoms in LiCl and LiBr crystals. This homogenized charge distribution on LiI facilitates uniform Li^+^ transport along the surface, thereby promoting homogeneous deposition of Li. From energy band structure perspectives, calculated band gaps reveal a critical correlation with ionic diffusion barriers. Narrow‐bandgap materials typically demonstrate lower Li^+^ diffusion barriers, which is particularly advantageous for achieving fast ion transport kinetics. Theoretical calculations identify LiI as possessing the narrowest band gap (4.235 eV, Figure S33a), significantly smaller than those of LiCl (6.312 eV, Figure S33b) and LiBr (5.216 eV, Figure S33c). This electronic structure characteristic corresponds to reduced energy barriers for Li^+^ migration in LiI. Density of States (DOS) and Partial Density of States (PDOS) analyses further confirm that the valence bands of these halides are predominantly governed by the p‐orbitals of halogen atoms (Figure S34). These computational insights provide fundamental guidance for developing advanced halogen‐based ionic conductors. To further elucidate the above view of lithium deposition behavior, the variation of Li^+^ concentrations and the distribution of lithium deposition during Li^+^ deposition using three different solid‐state electrolytes were explored by COMSOL Multiphysics field simulations (based on the actual electrochemical parameters of the three electrolytes).^[^ ^33^, ^34^ ^]^ With time evolution, severe ionic concentration polarization and heterogeneous Li^+^ distribution emerge rapidly in E‐LiCl and E‐LiBr electrolytes, directly inducing localized electric field distortions and consequent dendritic lithium growth (Figure S35). In contrast, the E‐LiI electrolyte maintains remarkably uniform Li^+^ concentration profiles throughout prolonged cycling simulations (Figure 4c). This exceptional ionic homogeneity enables steady interfacial potential distribution and dendrite‐suppressed lithium deposition. The combined theoretical calculation and COMSOL simulation results conclusively demonstrate that the LiI electrolyte ensures thermodynamically favorable Li^+^ redistribution and kinetically balanced plating/stripping processes, ultimately achieving stabilized electrolyte/electrode interfaces and prolonged cycling durability. This systematic investigation establishes fundamental structure‐property relationships for lithium halide electrolytes and provides critical design criteria for next‐generation lithium halide ion conductors and interfacial phases.

The influence of SEI composition on Li^+^ deposition behavior at cycled lithium metal anodes was further investigated by Cryo‐Transmission Electron Microscopy (Cryo‐TEM, Figure S36). High‐resolution TEM (HRTEM) images and corresponding Fast Fourier Transform (FFT) analysis revealed distinct crystalline components in the SEI layers that LiI predominated in E‐LiI‐derived interphase (Figure 4d), while LiCl and LiBr constituted the SEI phases in E‐LiCl and E‐LiBr, respectively (FigureS37, 38). Furthermore, complementary Time of Fight Secondary Ion Mass Spectrometry (TOF‐SIMS) and in‐situ Raman spectroscopy tests were performed, further providing evidence for the previously presented scenarios regarding the evolution of SEI on lithium metal surfaces (Figure 4e–h). TOF‐SIMS results confirm that the cycled Li metal surface in LFP/E‐LiI/Li cells forms a LiI‐rich SEI layer (Figures 4e and S39), directly corroborating our prior cryo‐TEM observations. Crucially, in situ Raman spectroscopy dynamically captured interfacial evolution during cycling, revealing progressive emergence and intensification of characteristic LiI Raman peaks on the Li surface (Figure 4 g,h), demonstrating real‐time LiI accumulation at the electrode‐electrolyte interface.^[^ ^35^, ^36^ ^]^ Notably, the LiI‐enriched SEI demonstrates superior capability in guiding homogeneous Li^+^ deposition. These collective results highlight the synergistic mechanism of E‐LiI electrolyte that the Mil‐100(Al) frameworks effectively restrict TFSI⁻ mobility while LiI facilitates rapid Li^+^ conduction, collectively enabling spatially uniform Li deposition and dendrite suppression. This dual‐functionality establishes critical prerequisites for achieving enhanced long‐term cyclability in lithium metal batteries.

**Figure 5:**
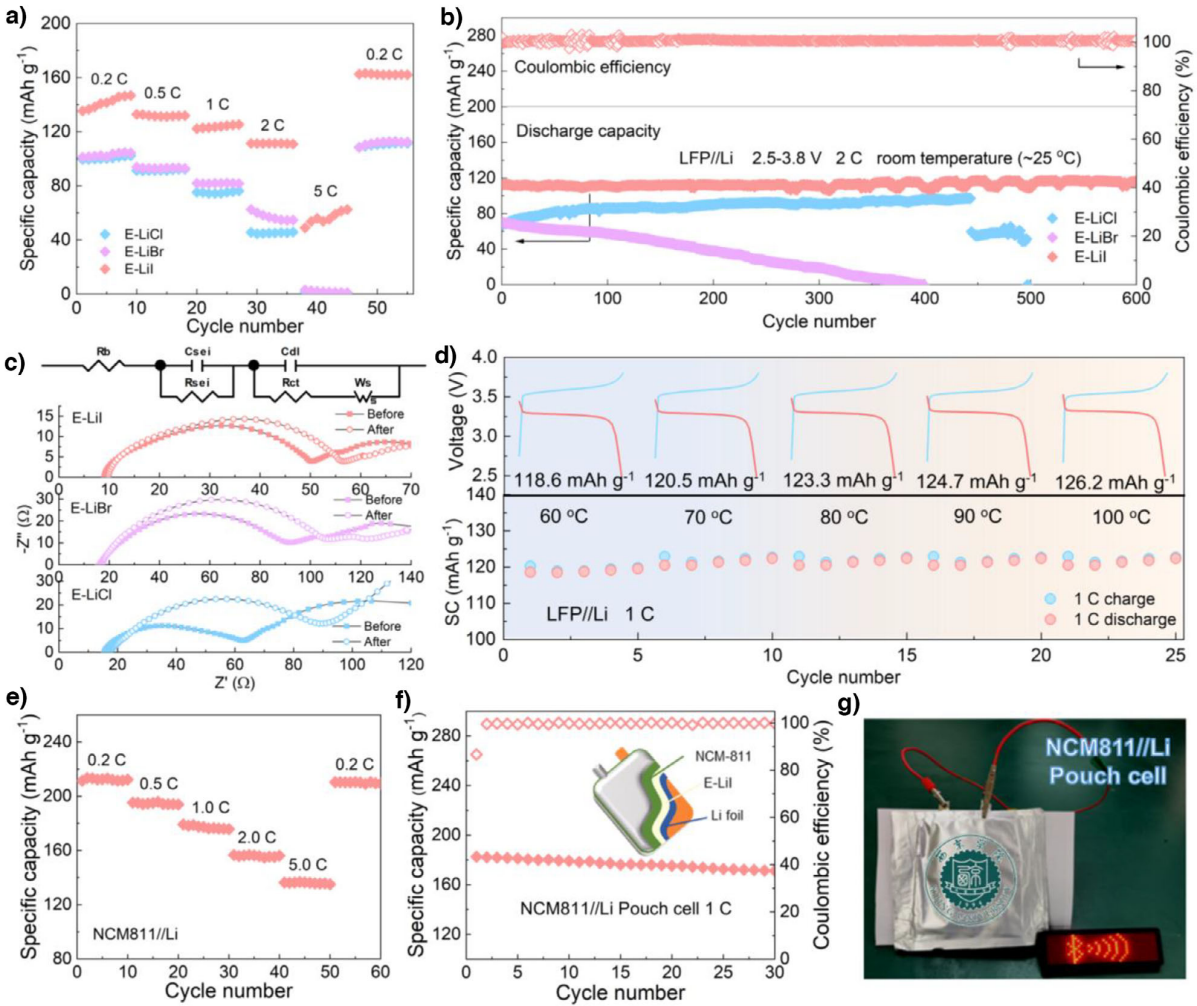
Performance of LiFePO_4_//Li and NCM811//Li solid‐state batteries. a) Rate performance of the LiFePO_4_//Li cells. b) Cycling stability of the LiFePO_4_//Li cells. c) Electrochemical impedance spectroscopy of the LiFePO_4_//Li cells before and after the rate test. d) Performance of LiFePO_4_/E‐LiI/Li batteries at different temperatures. e) Rate performance of the NCM811/E‐LiI/Li cell. f) Cycling stability of the NCM811/E‐LiI/Li pouch cell. g) Optical photograph of NCM811/E‐LiI/Li pouch cell.

To further validate the application potential of E‐LiI in SSLMBs, solid‐state electrolytes assembled with LiFePO_4_(LFP) and LiNi_0.8_Mn_0.1_Co_0.1_O_2_(NCM811) cathodes were investigated. The LFP/E‐LiI/Li cell delivered an initial discharge specific capacity of 132.4 mAh g^−1^ at 1 C and maintained stable operation over 200 cycles without capacity decay. In contrast, LFP/E‐LiCl/Li and LFP/E‐LiBr/Li cells exhibited significantly lower capacities (<100 mAh g^−1^, Figure S40). Furthermore, to meet the demand for diverse rate capabilities, the discharge capacities of LFP//Li cells were evaluated at various current densities (Figure 5a). The LFP/E‐LiI/Li cell demonstrated discharge capacities of ∼140, 135, 120, 115 and 80 mAh g^−1^ at 0.2, 0.5, 1, 2 and 5C, respectively, with negligible capacity loss upon returning to 0.2C, attributed to the rapid Li⁺ transport enabled by the E‐LiI electrolyte. By comparison, the capacities of LFP/E‐LiCl/Li and LFP/E‐LiBr/Li cells were markedly inferior, especially at 5C the capacity decays to almost 0 mAh g^−1^. Further investigations into the charge transfer resistance (Rct) at the electrode‐electrolyte interfaces before and after rate testing were conducted to elucidate the Li^+^ transport dynamics at the interface (Figure 5c). For LFP/E‐LiI/Li, the Rct increased slightly from 40.1 initially to 45.5 Ω after cycling. In contrast, both LFP/E‐LiCl/Li and LFP/E‐LiBr/Li exhibited higher initial Rct values of 44.7and 74.4 Ω, respectively, which further increased to 73.2 and 87.0 Ω after cycling. These results indicate larger initial resistances and greater increases in impedance for the LiCl and LiBr based systems, highlighting that the LFP/E‐LiI/Li configuration exhibits superior electrode‐electrolyte interfacial contact and a higher potential for facilitating uniform Li^+^ deposition. To address the demand for higher current densities and longer cycling lifetimes, long‐term stability evaluations of LFP//Li batteries assembled with three different electrolytes were conducted under a high current density of 2C. As shown in Figure 5b, the LFP/E‐LiCl/Li and LFP/E‐LiBr/Li batteries exhibited short‐circuiting and rapid capacity decay during cycling. This was attributed to the uneven deposition of Li^+^ on the lithium metal surface, leading to significant lithium dendrite formation and dead lithium accumulation. In contrast, the LFP/E‐LiI/Li battery demonstrated a discharge capacity close to 120 mAh g⁻¹ and maintained stable cycling for 600 cycles without noticeable capacity decay, showcasing excellent long‐term cycling performance. This result further highlights that the E‐LiI electrolyte effectively promotes uniform Li deposition and nucleation while suppressing lithium dendrite growth. Notably, compared with previously reported MOF‐based composite solid‐state electrolytes, the LFP//Li battery assembled with the E‐LiI electrolyte exhibited highly competitive performance (Table S4). To further validate the application potential of E‐LiI electrolyte under high‐temperature conditions, we conducted continuous cycling performance tests on the battery within a temperature range of 60∼100 °C. As shown in Figure 5d, the LFP/E‐LiI/Li cell exhibited a discharge capacity exceeding 120 mAh g^−1^, which opens possibilities for extreme environment applications and serves as a new paradigm for the design of high‐safety solid‐state electrolytes.

To demonstrate the potential of E‐LiI solid‐state electrolytes for use with high‐voltage cathode materials (NCM811) to assemble batteries with higher energy density, we first assembled NCM811/E‐LiI/Li batteries and tested their discharge capacities at different current densities within a voltage range of 2.7∼4.3 V. As shown in Figure 5e, the NCM811/E‐LiI/Li battery exhibited discharge capacities of 213.4, 195.3, 179.2, 156.7 and 136.4 mAh g^−1^ at 0.2, 0.5, 1, 2 and 5C, respectively. Upon returning to 0.2 C, the battery maintained a capacity of 210.4 mAh g^−1^, indicating that the E‐LiI electrolyte enables excellent fast charge/discharge capability in batteries paired with high‐voltage cathode materials. Finally, NCM811//Li pouch cells were assembled using E‐LiI electrolytes and NCM811 cathodes to evaluate their potential for high‐energy‐density practical batteries. As shown in Figure 5f, the resulting NCM811/E‐LiI/Li pouch cell exhibited outstanding capacity and stable cycling performance at 1C, while also being able to continuously power an LED panel (Figure 5g).

## Conclusion

In summary, a novel solid‐state electrolyte has been developed by encapsulating lithium halides (LiX, X═Cl, Br, I) within the mesoporous structure of MIL‐100(Al), combined with PVDF‐HFP polymer matrix. The three‐stage pore architecture of MIL‐100(Al) effectively confined LiX and optimized ion transport while restricting anion migration. The successful encapsulation and structural integrity have been confirmed by advanced characterizations. Moreover, Theoretical calculations and COMSOL simulations have elucidated the mechanisms by which lithium halides influence Li^+^ deposition and transport at the surface of lithium metal anodes, establishing a theoretical model for lithium halide@MOF ion conductors. The resulting electrolytes exhibited high ionic conductivity (5.2 × 10^−4^ S cm^−1^ for E‐LiI), superior Li⁺ interfacial kinetics, and stable cycling performance in LFP||Li cells (∼100% capacity retention after 600 cycles at 2 C). Pouch cells with E‐LiI also demonstrated stable operation with high‐voltage cathodes. This work highlights the importance of synergistic confinement effects and chemical compatibility in advancing solid‐state electrolytes for high‐energy‐density batteries.

## Conflict of Interests

The authors declare no conflict of interest.

## Supporting information



Supporting Information

## Data Availability

The data that support the findings of this study are available from the corresponding author upon reasonable request
