# Supplementary material for: Halogen‐Driven Ion Transport Homogenization in 3D Hierarchical MOF for Ultrastable Solid‐State Lithium Metal Batteries
Source: Angew Chem Int Ed Engl. 2025 Jul 20;64(37):e202511822. doi: 10.1002/anie.202511822 (PMC12416445; doi:10.1002/anie.202511822)
Supplement: Supplementary file 1 — Supporting Information [file ANIE-64-e202511822-s001.docx]

**Supporting information**

**Halogen-Driven Ion Transport Homogenization in 3D Hierarchical MOF for Ultrastable Solid-State Lithium Metal Batteries**

Xingxing Zhang,^a#^ Hongli Chen,^b#^ Qingmei Su,^c*^ Xinglong Deng,^d^ Dequn Zhao,^d^ Weihao Shi,^c^ Liming Wang,^c^ Jinqi Chen,^a,c^ Fan Xi,^a^ Zeming He,^a^ Ping Yu,^a*^ Guoxiu Wang,^b*^ Wenhuan Huang,^d,e*^

**Methods**

**Materials.**

The following chemicals were used as received without further purification: Poly (vinylidene fluoride-cohexafluoropropylene) (PVDF-HFP, Mw=4000000) was obtained from Sigma-Aldrich, benzene tricarboxylic acid (99.9%, C_9_H_6_O_6_), 1,3,5-Benzenetricarboxylic acid (99%, C_9_H_6_O_6_), Aluminium nitrate nonahydrate (Al(NO_3_)_3_·9H_2_O, 99.5%) were purchased from Shanghai Macklin Biochemical Technology Co., Ltd. Bis(trifluoromethane)sulfonimide lithium salt (LiTFSI, 99%), ethanol (CH_3_CH_2_OH, 99.8%) and 1-methyl-2-pirrolidone anhydrous (NMP, 99.5%) were obtained from Shanghai Aladdin Biochemical Technology Co., Ltd. Deionized water. LiFePO_4_ (LFP), LiNi_0.8_Co_0.1_Mn_0.1_O_2_ (NCM811), Super-P (99.9%) and polyvinylidene difluoride (PVDF), 2032 Coin cell shell were purchased from Guangdong Canrd New Energy Technology Co., Ltd. Aluminum foil (18 μm, 99.3%) and copper foil (9 μm, 99.8%) were supplied from Kejing Star Technology Corp.

**Characterizations**

Powder X-ray diffraction (PXRD) was performed using a diffractometer (D8-Discover, Bruker Co., USA) with a Cu-Kα X-ray radiation source (λ=0.154056 nm). The Raman spectroscopy was performed on a Renishaw Invia Raman spectrometer under a backscattering geometry (532 nm) to characterize the E-LiCl, E-LiBr and E-LiI electrolytes. The overall morphology and microstructure were investigated by Field Emission Scanning Electron Microscope (SEM) (Quanta-450-FEG, FEI Co., U.K.) with an acceleration voltage of 20 kV. The X-ray photoelectron spectroscopy (XPS) analysis was carried out by ESCALAB 250 Xi spectrometer (VG Scientific Co., UK), and all the binding energies obtained in XPS spectra were calibrated using the C 1 s peak at 284.6 eV. The cycled Li metal anode was transferred into a glovebox with a sealed Ar-filled vessel for SEM and XPS examinations. All cryo-TEM characterizations were performed on a Double spherical aberration correction transmission electron microscope (JEM-ARM300F) operated at 300 kV with a 31660 cryo-transfer holder. The nanostructure and elemental distribution of SEI components within the electrolyte membrane were studied by adding liquid nitrogen at -170 °C. The cycled Li metal anode was transferred into a glovebox with a sealed Ar-filled vessel for SEM, XPS, and TEM examinations. Time-of-flight secondary ion mass spectrometry (TOF-SIMS) analysis was performed using a PHI nano TOF||Time-of-Flight SIMS equipped with a 30 kV Bi-cluster liquid metal ion gun.

**Synthesis of Mil-100(Al)**

Mil-100(Al) was synthesized via a hydrothermal route according to previously reported procedures. 3.5 g of Al(NO_3_)_3_·9H_2_O was completely dissolved in 60 mL of deionized water under magnetic stirring. Subsequently, 4 mol/L nitric acid was slowly added dropwise to adjust the pH value while maintaining continuous agitation. Following this, 1.5 g of 1,3,5-benzenetricarboxylic acid was introduced into the homogeneous solution under vigorous stirring. The resultant mixture was transferred into a 100 mL Teflon-lined stainless-steel autoclave and subjected to hydrothermal treatment at 210 °C for 4 h. After natural cooling to room temperature, the obtained product was isolated by filtration, thoroughly washed with deionized water, and dried under vacuum to yield a pale-yellow powdery sample, denoted as Mil-100(Al).

**Synthesis of LiX@Mil-100(X=Cl, Br, I)**

In a typical synthesis procedure, 0.5 g of LiX (X = Cl, Br, I) was separately dissolved in 20 mL of methanol under vigorous stirring until complete dissolution. Subsequently, 1.0 g of Mil-100(Al) powder was added to each LiX-containing solution. The resulting mixtures were continuously stirred for 24 h at ambient temperature. The solids were then collected via filtration and thoroughly washed with methanol three times to eliminate residual LiX species adsorbed on the Mil-100 surfaces. Finally, the samples were dried under vacuum to obtain the lithium-incorporated ionic conductors, designated as LiCl@Mil-100, LiBr@Mil-100, and LiI@Mil-100, respectively.

**Preparation of E-LiX (X=Cl, Br, I) solid-state electrolytes**

Typically, 1 g of PVDF-HFP and 0.125 g of LiTFSI are dispersed in 20 ml of NMP to form a homogeneous solution. Subsequently, 1 g of LiCl@Mil-100, LiBr@Mil-100 and LiI@Mil-100 with same mass were added, respectively. The mixture was stirred at 60 °C overnight. Finally, the uniformly stirred solution was cast on the polytetrafluoroethylene mold. After the solvent was evaporated, the film was further dried under vacuum at 60°C. Upon evaporation of the solvents, the resulting polymer electrolyte could be peeled off as a self-standing membrane (denoted as E-LiCl, E-LiBr and E-LiI).

**Assembly of solid-state batteries**

The prepared E-LiCl, E-LiBr and E-LiI membranes were cut into 19 mm diameter discs and placed in a glove box filled with argon atmosphere. PVDF powder was dried overnight at 100 °C under vacuum to remove moisture before use. Commercial NCM811/LiFePO_4_, super-P and PVDF were mixed in NMP with a mass ratio of 8:1:1 to obtain the cathode mixture. The slurry was then bladed on Al foil to render uniform coating, which was further dried in a vacuum oven at 100 °C for at least 48 h and the mass loading not less than 5 mg cm^-1^. Different solid electrolyte membranes were used (thickness of ~50 μm) to replace polypropylene (PP) as separator. CR2032-type coin cells in an Ar-filled glove box (both O_2_ and H_2_O contents were below 0.01 ppm) were assembled for electrochemical tests (In order to improve the poor solid-solid contact between the solid electrolyte and electrode, 5 μL trace lithium secondary liquid electrolyte was added). The NCM811/LFP//Li full cells were assembled to test cycle performance. The Li//Li and Li//Cu cells were assembled to test Li deposition performance. For NCM811/E-LiI/Li pouch cells, NCM-811 cathode (mass loading: 5 mg/cm^2^, 4×4.5 cm) and Li metal anode (4.5×5 cm, 50 μm Li) were stacked one by one and separated by E-LiI electrolyte (5×5.5 cm).

**Electrochemical measurements**

Ionic conductivity was determined by EIS after placing the solid electrolyte between two electrodes (stainless steel) contacts in a CR2032 coin cell. CHI660E electrochemical workstation (Shanghai, China) was used for measurements at various temperatures from 25 to 80 °C with a step size of 10 °C and frequency ranges from 10^6^ to 0.1 Hz. The bulk resistance of the samples was calculated from the EIS curve. The ionic conductivity was calculated according to the following equation (1):

$$\sigma=\frac{d}{SR_{b}} (1)$$

where *d*(cm) was the thickness of the solid electrolyte, *R*_b_(Ω) and *S*(cm^2^) represented the bulk resistance and the effective area of the solid electrolyte, respectively, and σ is the ionic conductivity (S cm^-1^).

The activation energy E_a_ was calculated according to Arrhenius equation (2):

$$\sigma=AT^{-1}e^{-E_{a}/RT} (2)$$

where A was the characteristic constant of the given reaction, e is the base of the natural logarithm (2.718), R is the gas constant (8.314 J mol^-1^ K^-1^), T is the thermodynamic temperature, and *E*_a_ is the activation energy (kJ mol^-1^).

The electrochemical stability of the electrolyte was investigated by linear sweep voltammetry (LSV) on a Chenhua CHI660E electrochemical workstation. The lithium|solid electrolyte|stainless steel cell was scanned from 0 to 6 V at a scan rate of 10 mV s^-1^ at 25 ^o^C.

The lithium transference number (t_Li_^+^) was obtained by using AC impedance and DC potentiostatic polarization measurements with Li//Li symmetric cell. A DC potential (ΔV=10 mV) was applied for 5000 s to gain the initial and steady currents. Meanwhile, the AC impedance spectra of the same cell were measured before and after polarization. The value of *t_Li_^+^* was been calculated by equation (3):

$$t_{{Li}^{+}}=\frac{I_{s}(\Delta V-I_{0}R_{0})}{I_{0}(\Delta V-I_{S}R_{S})} (3)$$

Where *R*_0_ and *R*_s_ are the AC impedances before and after polarization, respectively. *I*_0_ and *I*_s_ are the initial and steady currents respectively.

The galvanostatic discharge-charge performances of the cells were evaluated on a *Neware* battery test system at room temperature. The half cells of Li-Cu were operated for the initial three cycles at 0.01-1.0 V at 0.05 mA cm^-2^ to stabilize the SEI. Subsequently, deposit lithium onto a Cu foil for 10 h. The Li//Li symmetric cells were cycled using bare Li anode at 1 mA cm^−2^ with a capacity of 1 mAh cm^-2^. The LMBs were assembled using LiFePO_4_ (1C=170 mAh/g) as cathode and metallic lithium as cathode and NCM811 (1C=200 mAh/g) as cathode and metallic lithium as cathode. The charge and discharge tests of LiFePO_4_ǁLi cells were carried out between 2.5 and 3.8 V and NCM811ǁLi cells were carried out between 2.7 and 4.3 V.

**Computational Methods and Details**

All theoretical calculations in this work were performed using *Materials Studio 2019* based on density functional theory (DFT). The calculations employed two complementary modules with specific methodologies as described below.

**DMol³ Calculations for Adsorption Energies, Migration Barriers and Surface Energies**

Geometry optimizations and energy calculations for Li⁺ adsorption and migration were conducted using the *DMol3* module. We employed the Perdew-Burke-Ernzerhof (PBE) generalized gradient approximation (GGA) functional for exchange-correlation interactions. The double numerical plus polarization (DNP) basis set with orbital cutoff of 4.4 Å was used with all-electron treatment. Monkhorst-Pack k-point meshes of 3×3×1 were applied for Brillouin zone sampling in slab models (surface thickness >15 Å). The self-consistent field (SCF) convergence thresholds were set to 1×10⁻⁵ Ha for energy and 1×10⁻^6^ Ha for electron density. Structural optimizations were fully converged with maximum force <0.002 Ha/Å, maximum displacement <0.005 Å, and energy change <1×10⁻^5^ Ha.

The Li⁺ adsorption energy (𝐸_ads_​) was calculated as (4):

$$E_{ads}=E_{Li+/substrate}-E_{Li+}-E_{substrate} (4)$$

where *E*_Li+/substrate_​ is the energy of the adsorption system, *E*_substrate_​ represents the pristine surface (LiCl, LiBr or LiI), and *E*_Li+​_ denotes the energy of an isolated Li⁺ ion with charge correction using the vacuum level method.

Migration energy barriers were determined through linear synchronous transit (LST) and quadratic synchronous transit (QST) transition state searches. Each transition state was verified to have one imaginary frequency (<200 cm⁻¹) through vibrational frequency analysis. The migration barrier (Δ𝐸) was defined as (5):

$$\Delta E=E_{TS}-E_{Initial} (5)$$

where *E*_TS_​ is the transition state energy and *E*_Initial_​ is the initial state energy.

To compute surface energy in Materials Studio, first optimize the bulk crystal structure in CASTEP. Record the optimized bulk energy 𝐸bulk*E*bulk​. Subsequently, cleave the target surface to construct an asymmetric slab model with ≥3 atomic layers thickness and ≥15 Å vacuum layer. Apply Make P1 symmetry treatment, fix the bottom 2–3 atomic layers, and perform surface relaxation. The Li⁺ with LiX of surface energy (𝐸_surf_) was calculated as (6):

$$E_{Surf}=\frac{E_{Slab}-nE_{Bulk}}{2A} (6)$$

where E_slab_ is the total energy of the optimized slab model, E_slab_ is the bulk energy of a perfect crystal unit cell per atom, A is the surface area of the slub model. N is the atomic numbers in slab.

***CASTEP* Calculations for Electronic Structure Properties**

Electronic structure analyses (differential charge density, band structure, and density of states) were performed using the *CASTEP* module. Norm-conserving pseudopotentials with a plane-wave cutoff energy of 408 eV were employed (validated through convergence testing), and the PBE-GGA functional was used consistently. Slab models with >20 Å vacuum layers were sampled with a Monkhorst-Pack k-point mesh of 4×4×4.

The differential charge density (Δ𝜌) was computed as (7):

$$\Delta\rho=\rho_{total}\left( \frac{{Li}^{+}}{substrate} \right)-\rho_{substrate}-\rho_{Li+} (7)$$

with is values set at ±0.005 e·Å⁻^3^. Band structures were calculated along high-symmetry paths (e.g., Γ–M–K–Γ) using Fermi level (E_F) as 0 eV reference. Density of states (DOS) analysis included total DOS and projected DOS (PDOS) decomposed into atomic orbital contributions (s, p orbitals for Li, Cl, I and Li) from converged ground-state structures, with Gaussian smearing of 0.1 eV.

**Structural Models and Validation**

All surfaces were modeled as 3-layer slabs with bottom atoms fixed to mimic bulk environments. Migration paths were initially mapped through potential energy surface scanning and refined via LST/QST. Critical parameters (cutoff energy, k-point density) were validated through energy convergence tests. Transition states were additionally confirmed using the nudged elastic band (NEB) method. Computational protocols follow established benchmarks in ionic migration studies.

**Molecular dynamics (MD) simulations**

Diffusion coefficients in this work were calculated using molecular dynamics (MD) simulations performed with the Forcite module in *Materials Studio* software, simulations utilized the COMPASS III force field. The amorphous initial configuration was constructed using the Amorphous Cell tool and stabilized through sufficient geometry optimization followed by a 20 ps NVT ensemble relaxation. Subsequently, the system was equilibrated at the target conditions (temperature: 298 K) for 100 ps in the NPT ensemble, employing the Andersen thermostat and Berendsen barostat with a time step of 1 fs. Production dynamics were then conducted in the NVT ensemble for 300 or 500 ps with a 1 fs timestep. Atomic trajectories were recorded every 1 ps, yielding 300 or 500 frames for analysis.

The self-diffusion coefficient (*D*) was computed from the mean squared displacement (MSD) using the Einstein-Smoluchowski relation (8):

$$D=\frac{1}{6N}\lim_{n\to\infty} \left\langle\sum_{i=1}^{N} \left| \vec{r_{i}} \right. \right.\left( t \right)-\vec{r_{0}}\left. \left( 0 \right) \right|^{2} (8)$$

where *N* is the number of diffusing particles, and *𝑟⃗𝑖(𝑡)ri​(t)* denotes the position vector of particle *i* at time *t*. *D* was extracted as 1/6 of the slope of the linear region (*t₁*–*t₂*) in the MSD versus time curve, determined via constrained linear regression (goodness-of-fit *R²* > 0.99). Data from only the stable diffusive regime were considered. For ionic systems (e.g., electrolytes), the diffusion coefficients of cations (*D₊*) and anions (*D₋*) were calculated separately.

**Finite element simulations.**

In order to simulate the Li deposition behaviors, a Finite Element Analysis (FEA) model was performed using COMSOL Multiphysics 6.2 software with the “Tertiary Current Distribution” and “Phase Field” module. The size of the entire two-dimensional model for electric field distribution analysis was set to 40×30 μm. A transient simulation of the process was carried out in an area filled with electrolyte. The plating process was simulated using the phase field method.

The Li^+^ transfers by the concentration diffusion in model follow the Fick's law as shown in equation (9) and (10):

$$N_{i}=J_{i}={-D}_{i}\nabla c_{i} (9)$$

$\frac{\partial c_{i}}{\partial t}+\nabla J_{i}=R_{i,tot} (10)$

where $J_{i}$ is the ion flux, $D_{i}$ is the diffusion coefficient of electrolytes, $c_{i}$ is the ion concentration of electrolytes, $\nabla c_{i}$ is concentration gradient.

The relation between the diffusion coefficient and electric mobility follows the Nernst-Einstein relation as shown in equation (11):

$$N_{i}={-D}_{i}\nabla c_{i}-z_{i}u_{m,i}Fc_{i}{\nabla\emptyset}_{l}+uc_{i}=J_{i}+uc_{i} (11)$$

where $z_{i}$ is the transfer number ($z_{Li}=1$), $u_{m,i}$ is the electric mobility coefficient, $F$ is the Faraday constant (96485 C mol^-1^), 𝜙 is the electrolyte potential.

The equilibrium potential of the electrode surface follows the Nernst equation as shown in equation (12) and (13):

$E_{eq}=-\frac{\Delta G}{nF}$ (12)

$E_{eq}=E_{eq,ref}-\frac{\mathrm{RT}}{nF}ln\prod_{i} \left( \frac{\alpha_{i}}{\alpha_{i,ref}} \right)^{v_{i}} (13)$

where $E_{eq}$ is the electrode potential, $E_{eq,ref}$ is the standard electrode potential, $\Delta G$ is the Gibbs free energy, R is the ideal gas constant, T is the temperature, $n$ is the transfer electron number of the reaction, $\alpha_{i}$ is the electrode reactive ion concentration, $\alpha_{i,ref}$ is the standard electrode reactive ion concentration, $v_{i}$ is the reaction stoichiometric number.

The electrode reaction for the electrode surface follows the Butler-Volmer kinetics expression, as shown in equation (14):

$$i_{loc}=i_{0}\left( exp\left( \frac{\alpha_{a}F\eta}{RT} \right)-exp\left( \frac{-\alpha_{c}F\eta}{RT} \right) \right) (14)$$

where $i_{loc}$ is the local current density at the electrode/electrolyte interface, $i_{0}$ is the exchange current density, and is the cathodic and anodic charge transfer coefficients, $\eta$ is the activation overpotential.

The Comsol simulation parameters are shown in the table and figure below:

| Parameter | Sample | Value (Unit) |
| --- | --- | --- |
| Diffusion Coefficient | E-LiCl | 7.27e-12 (m²/s) |
|  | E-LiBr | 3.19e-12 (m²/s) |
|  | E-LiI | 3.91e-11 (m²/s) |
| Exchange current density | E-LiCl | 0.095 (mA/cm²) |
|  | E-LiBr | 0.107 (mA/cm²) |
|  | E-LiI | 0.165 (mA/cm²) |
| Ionic conductivity | E-LiCl | 3.4e-4 (S/cm) |
|  | E-LiBr | 4.3e-4 (S/cm) |
|  | E-LiI | 5.2e-4 (S/cm) |
| Initial concentration | All | 1 (M) |
| Faraday constant | All | 96485 (C/mol) |
| Temperature | All | 25 (^o^C) |
| Transfer number | All | 1 |
| Charge Transfer Coefficient (Cathode) | All | 1.5 |
| Charge Transfer Coefficient (Anode) | All | 0.5 |
| Transfer electron number of the reaction | All | 1 |
| Mesh Size | All | The physical field control grid has a coarser cell size |

**
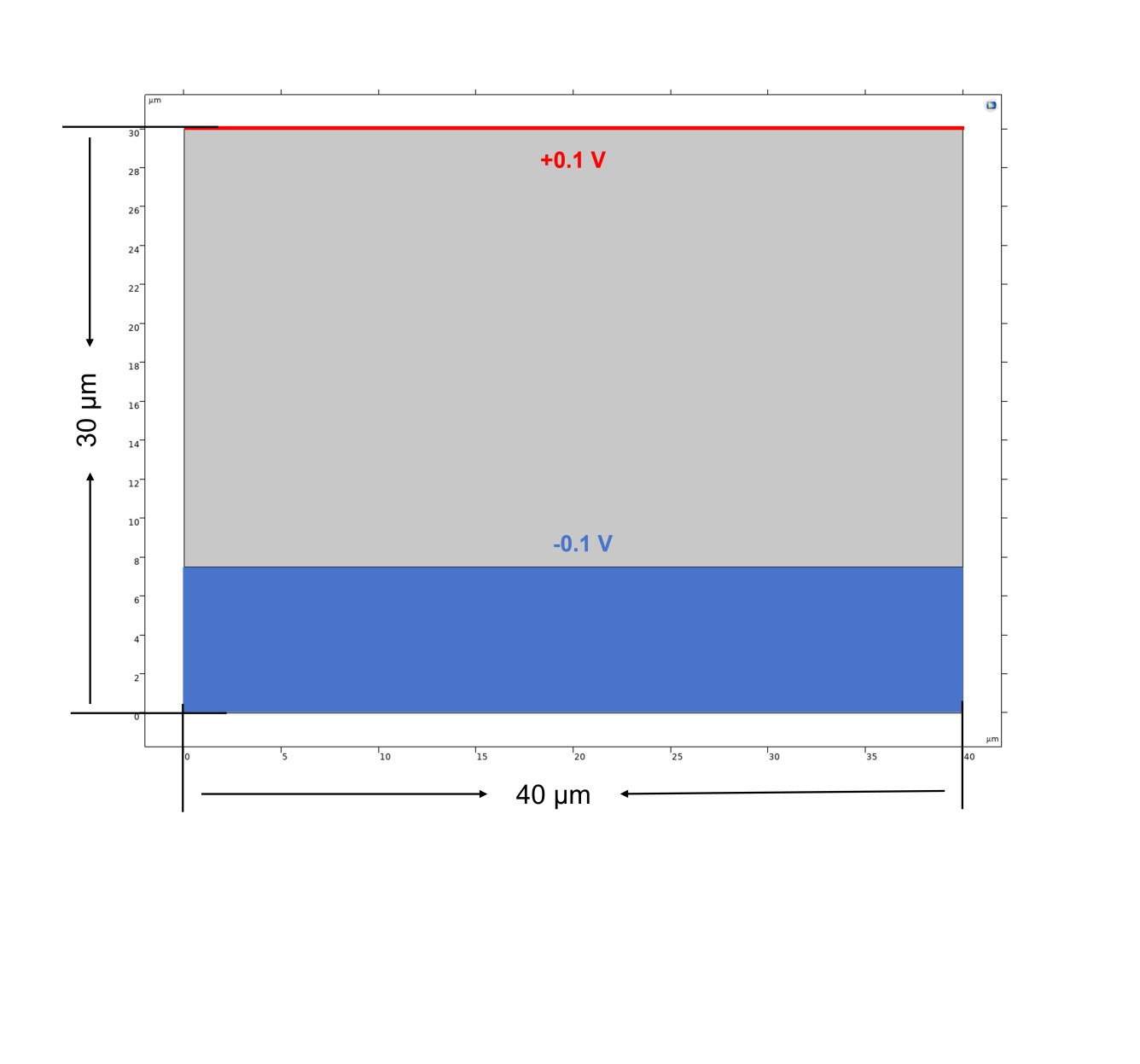
**

**Figures**


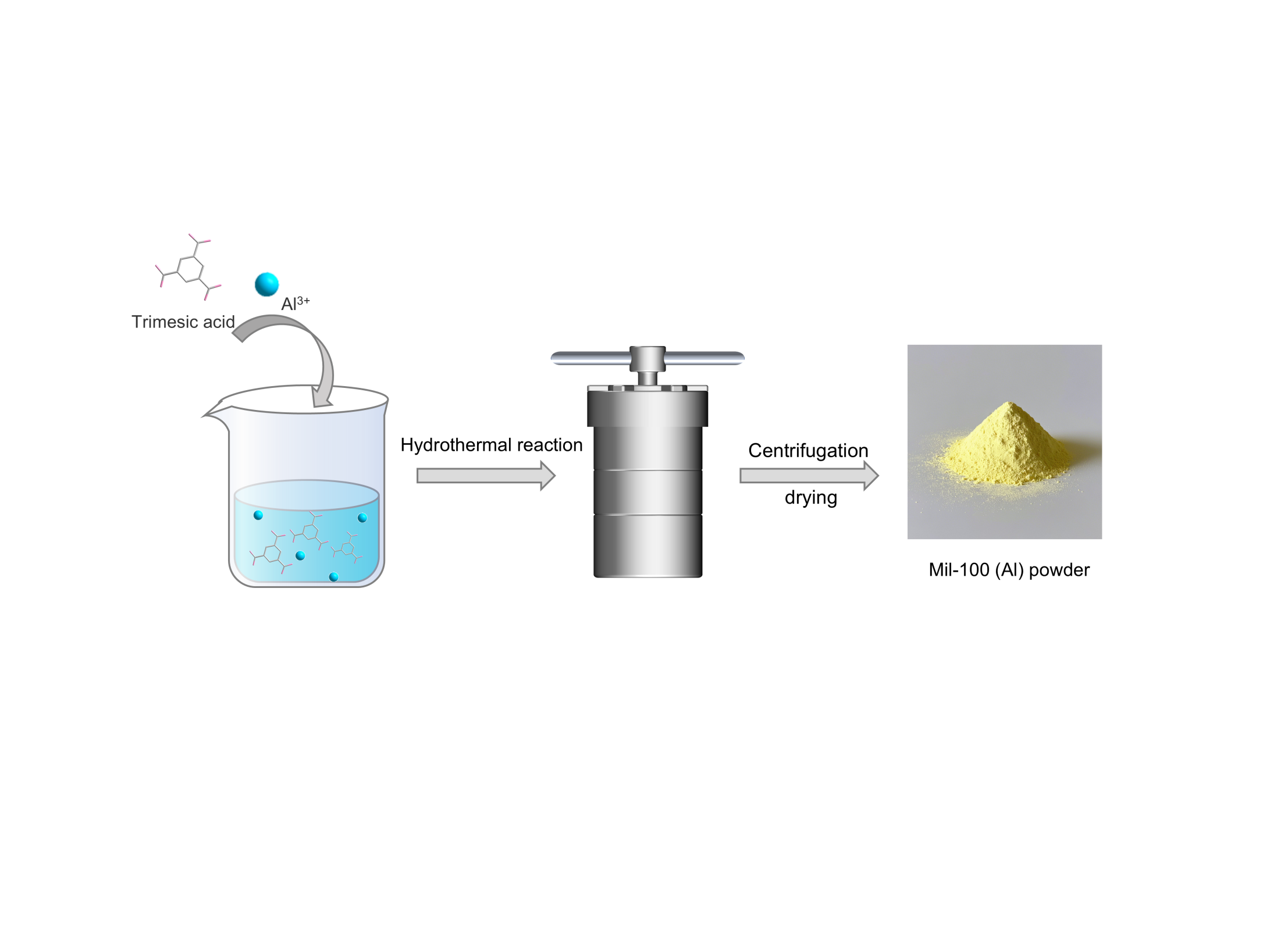


**Figure S1.** Schematic diagram of the preparation of Mil-100(Al) powder.

**
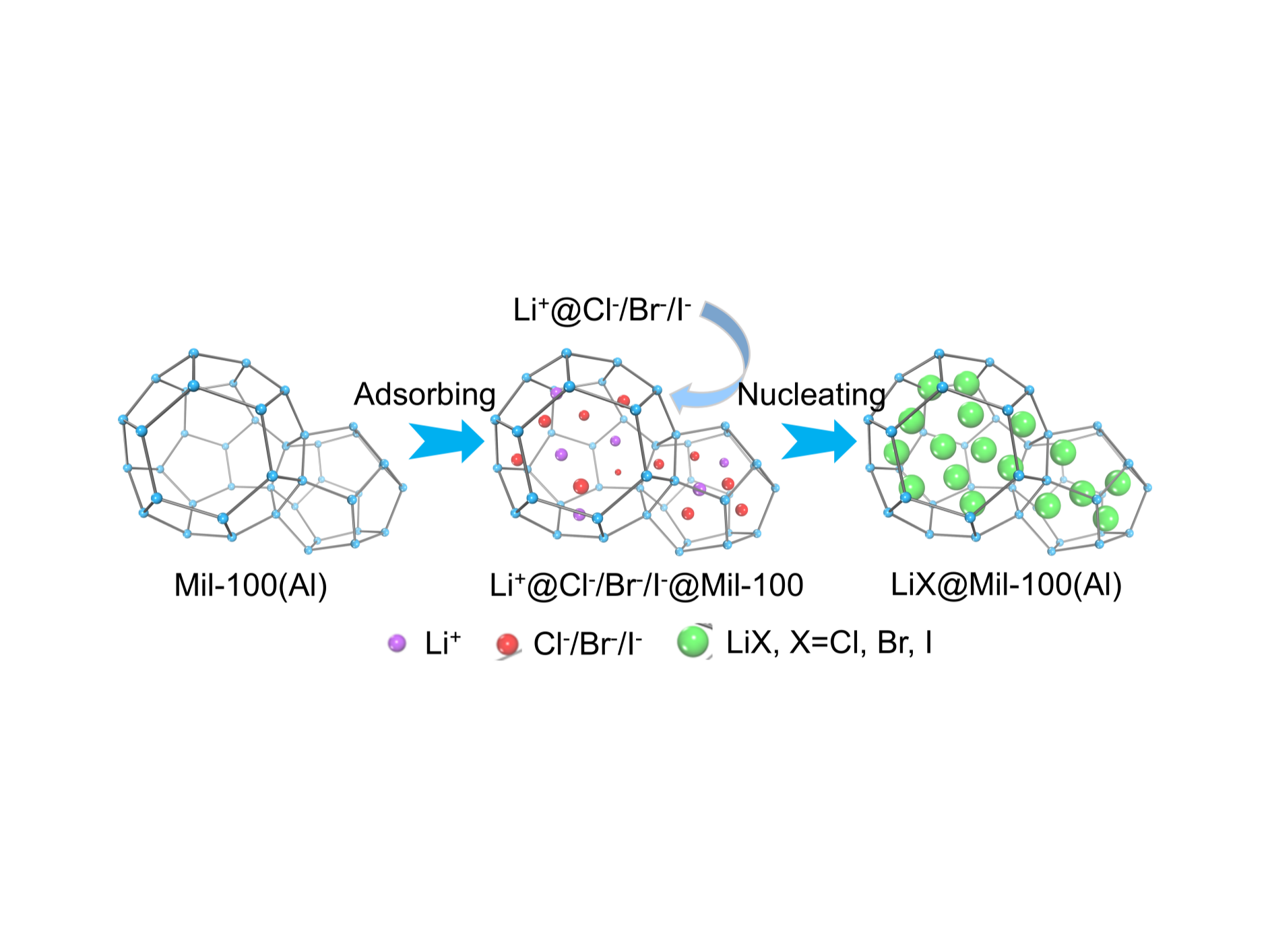
**

**Figure S2.** Schematic diagram of preparation of LiX@Mil-100(Al) composite ionic conductor.


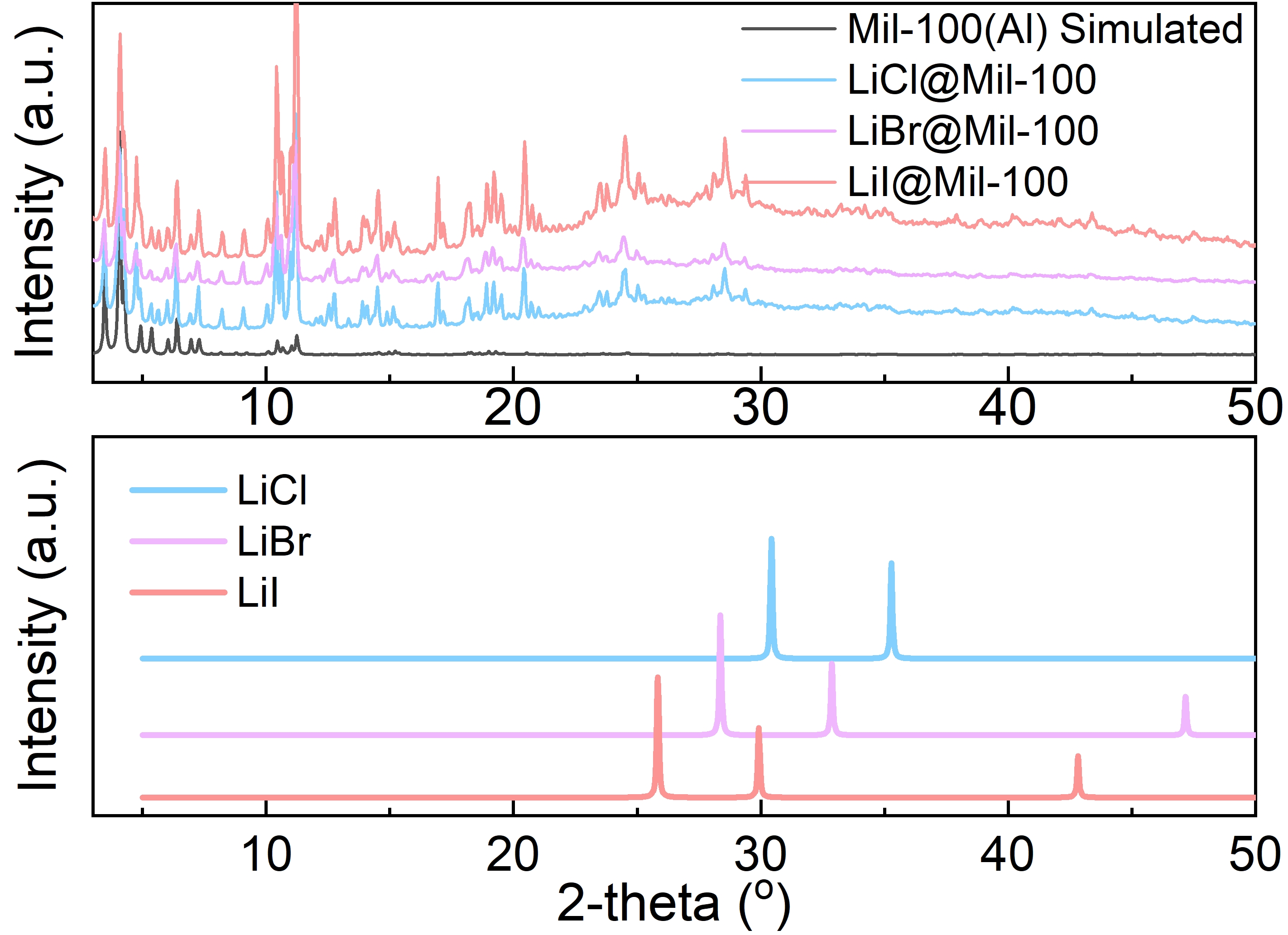


**Figure S3.** PXRD pattern of Mil-100(Al), LiCl@Mil-100, LiBr@Mil-100, LiI@Mil-100 and LiX (X=Cl, Br and I) powder.


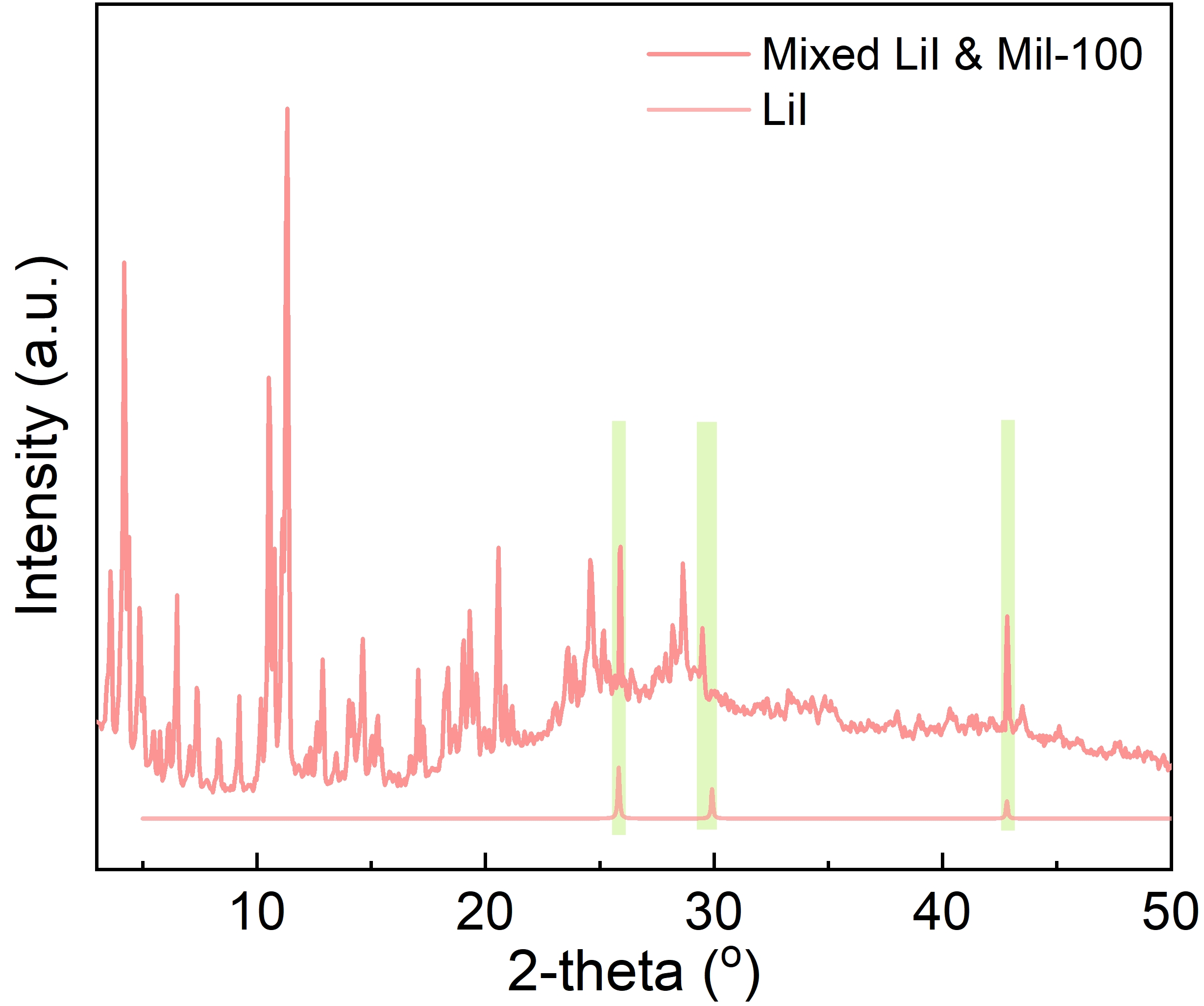


**Figure S4.** XRD patterns of LiI and mixed LiI & Mil-100.

**
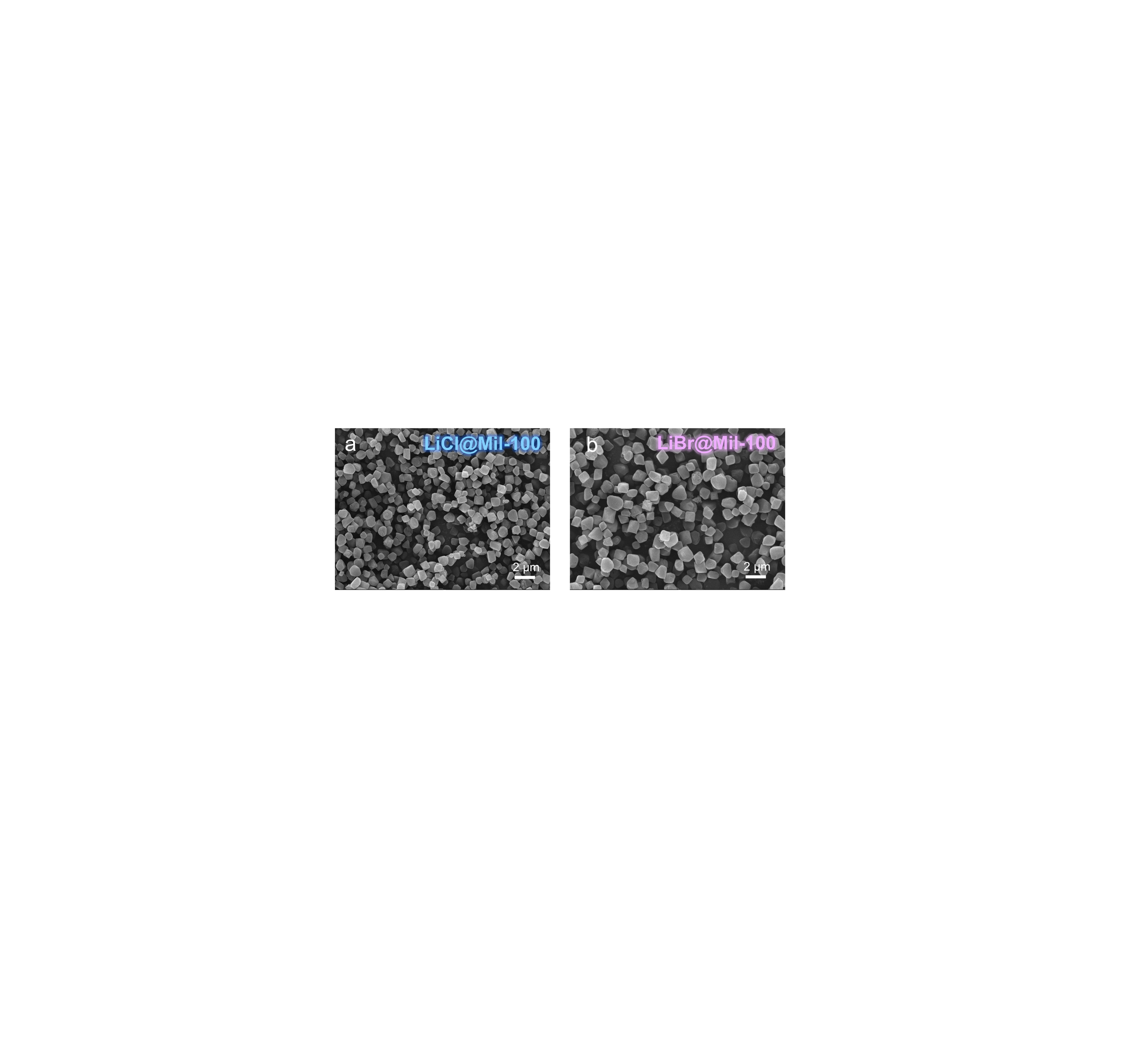
**

**Figure S5.** SEM images of (a) LiCl@Mil-100 and (b) LiBr@Mil-100,


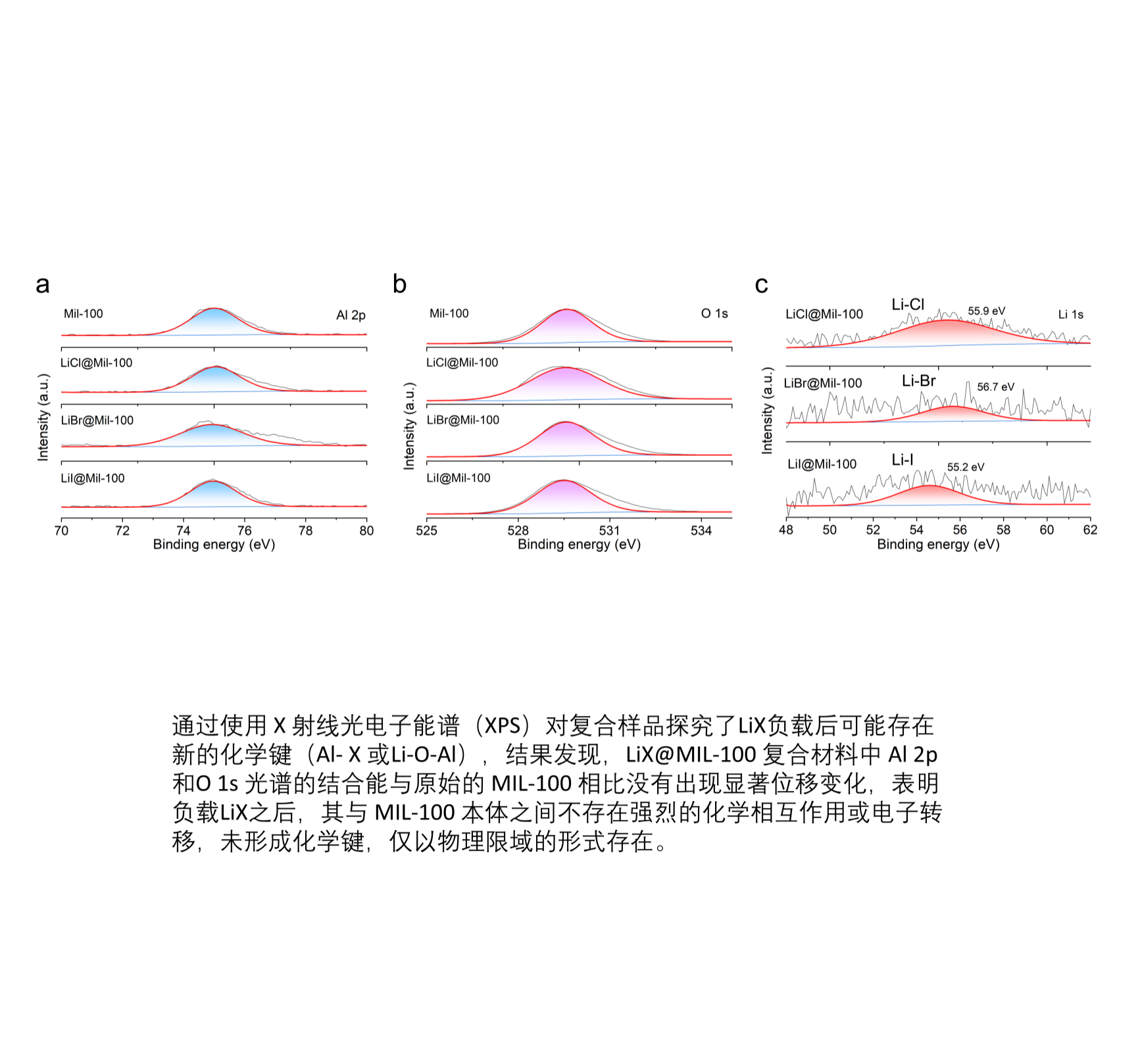


**Figure S6.** (a) Al 2p, (b) O 1s and (c) Li 1s XPS spectra of Mil-100 and LiX@Mil-100.


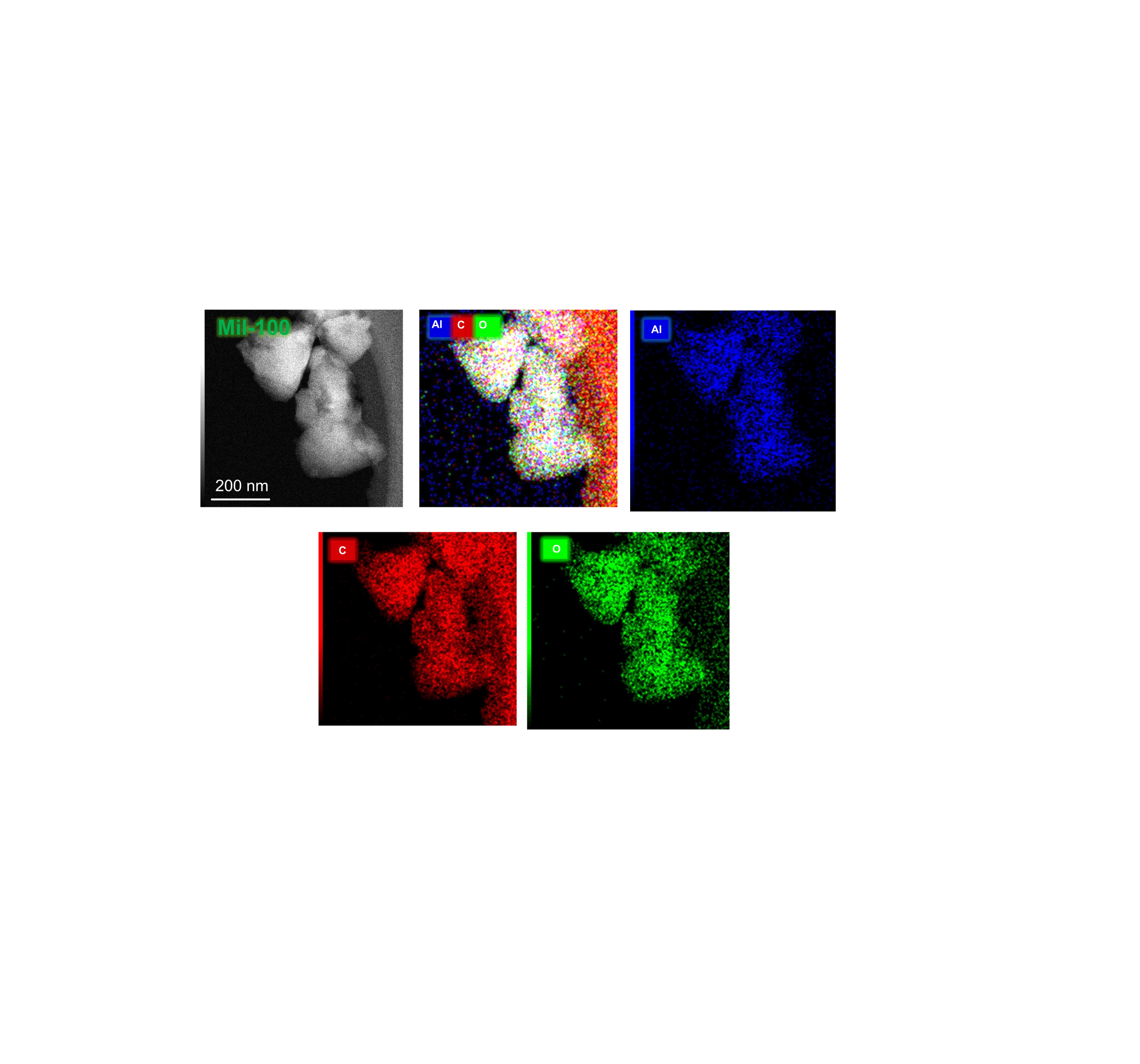


**Figure S7.** TEM-EDS mapping of Mil-100.


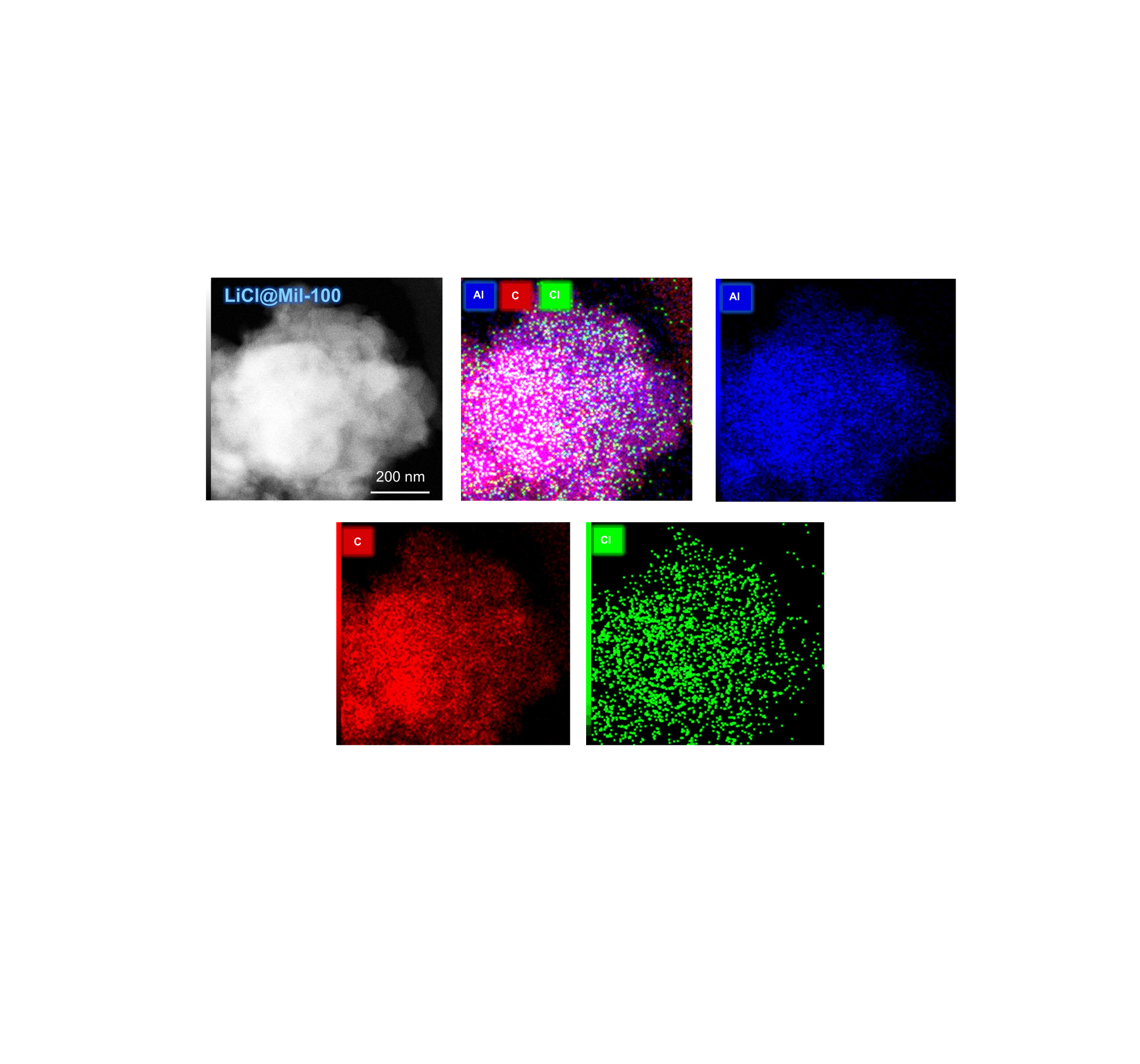


**Figure S8.** TEM-EDS mapping of LiCl@Mil-100.


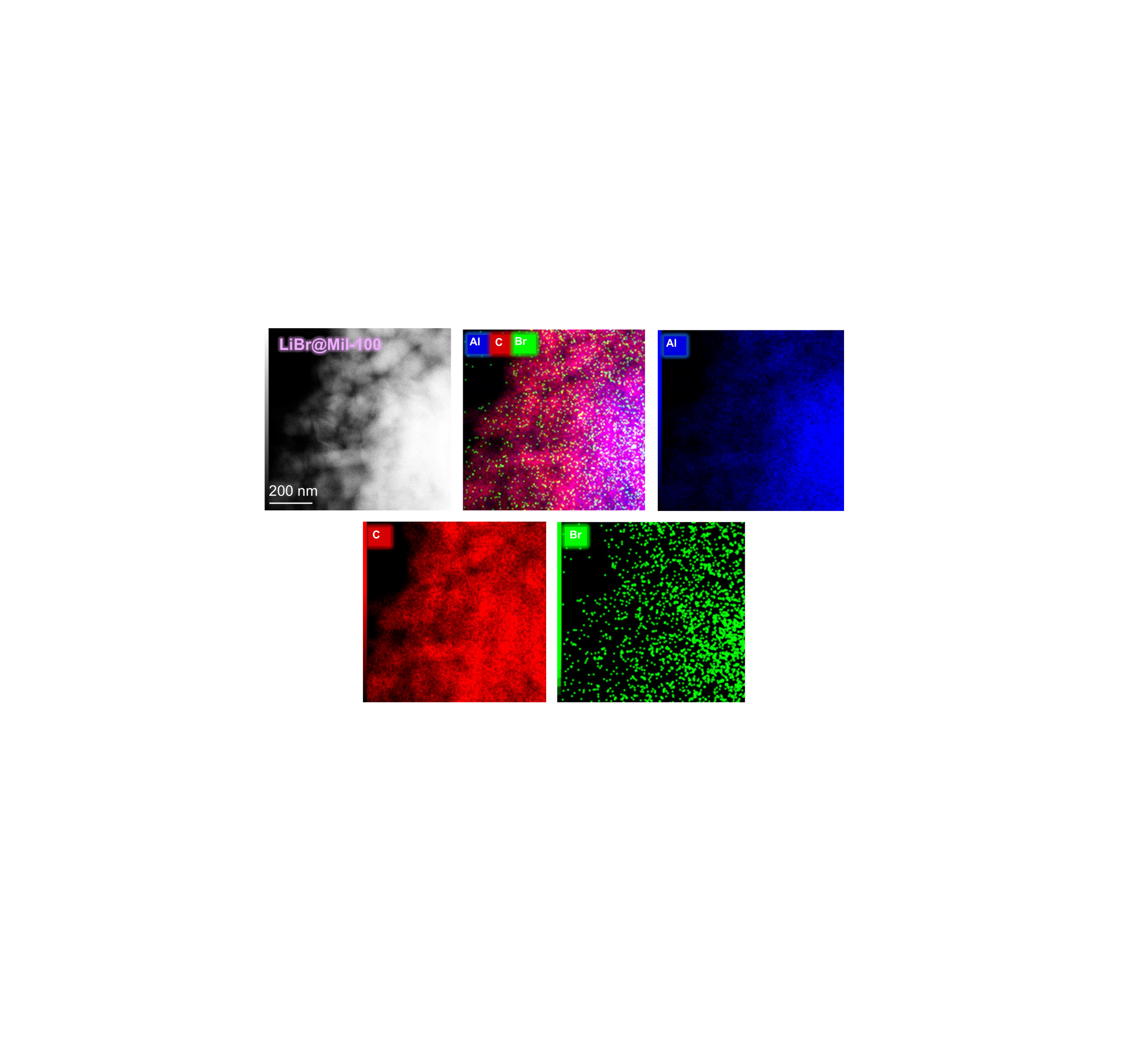


**Figure S9.** TEM-EDS mapping of LiBr@Mil-100.


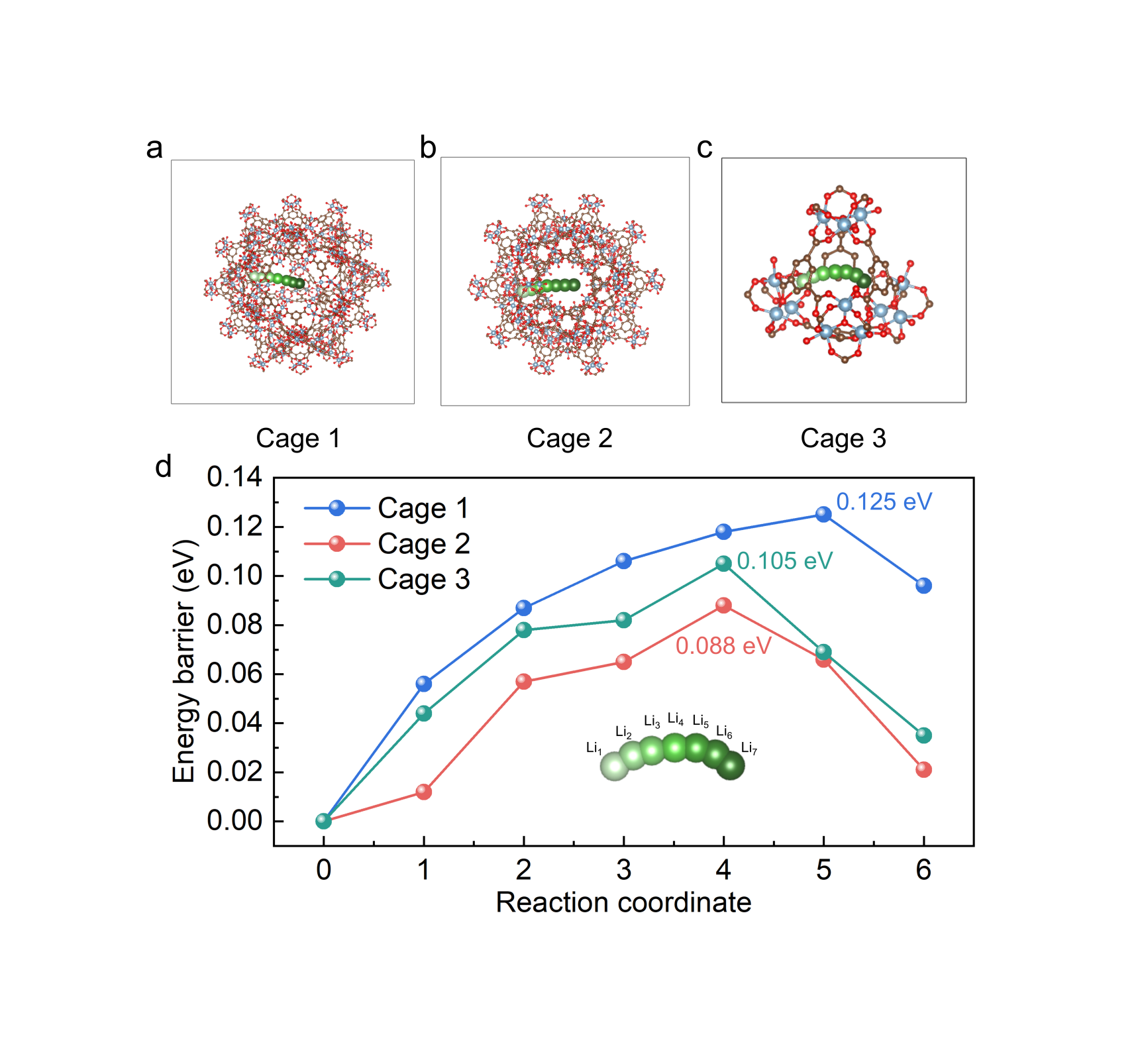


**Figure S10.** Migration paths and migration energy barriers of Li^+^ in different cages of Mil-100.

**
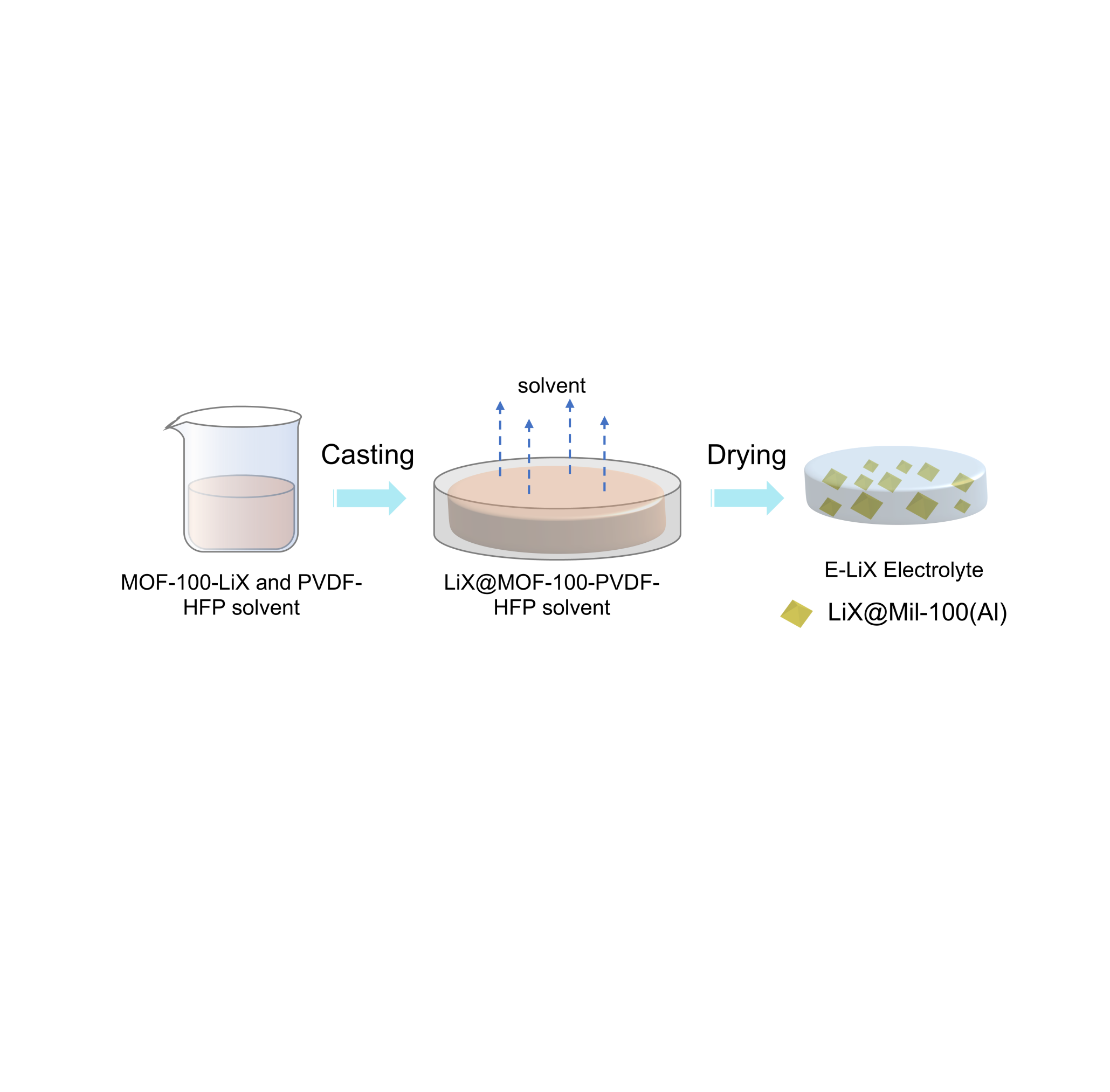
**

**Figure S11.** Schematic illustration of the preparation of the E-LiX electrolytes.

**
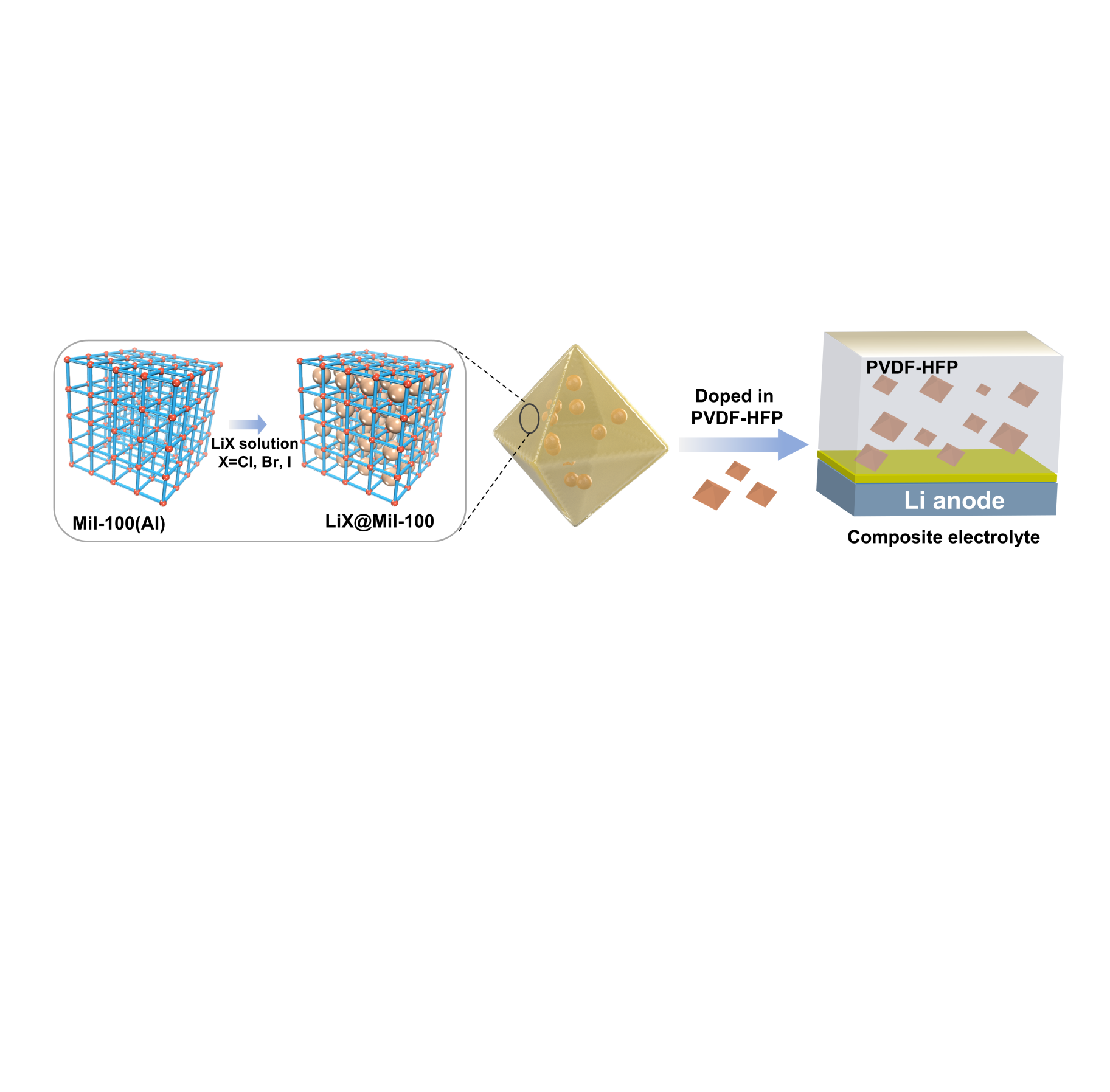
**

**Figure S12.** Schematic diagram of preparation of composite electrolyte.

**
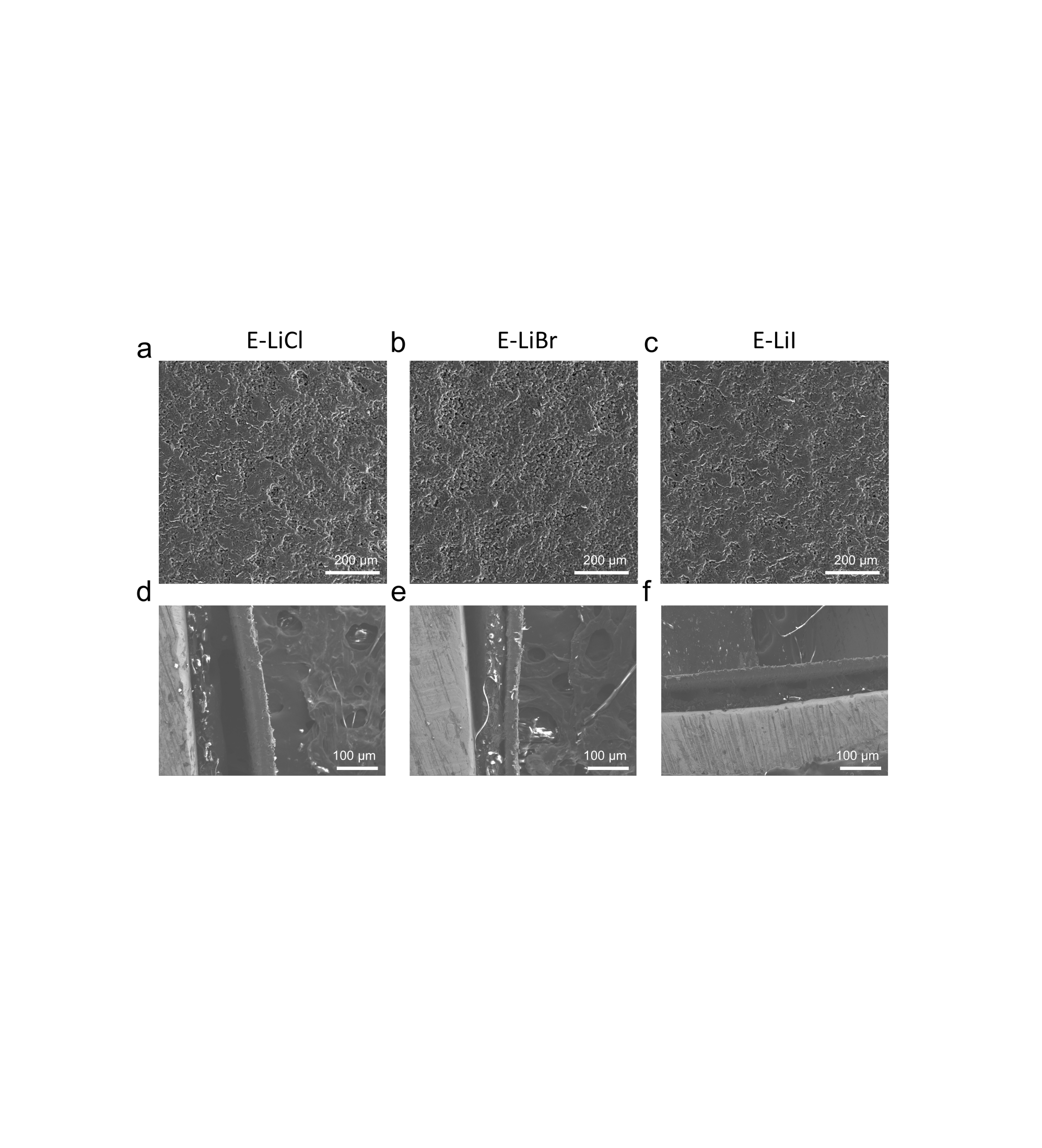
**

**Figure S13.** Surface and cross-sectional SEM images of E-LiCl, E-LiCl and E-LiI electrolyte membranes.


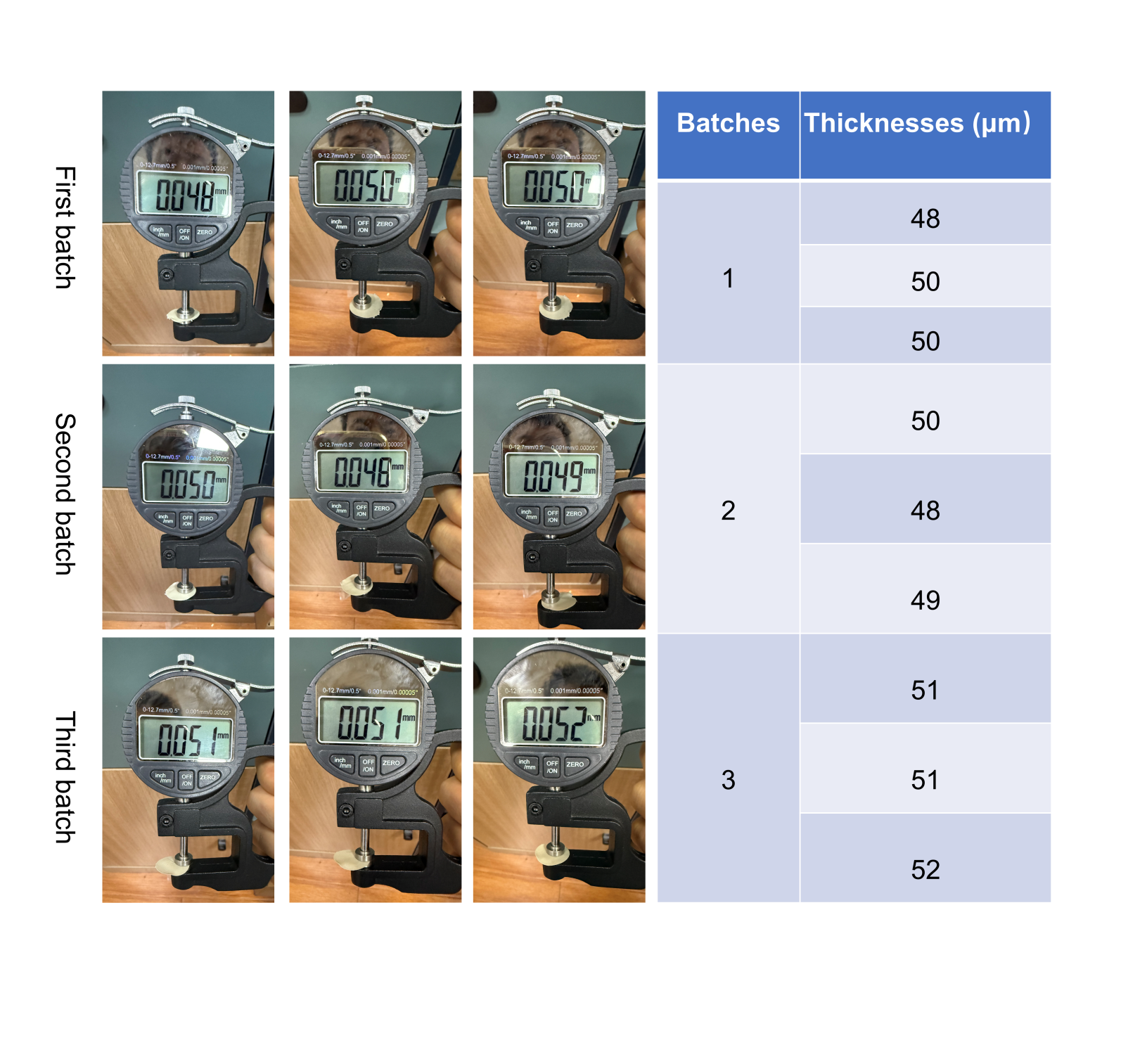


**Figure S14.** Thickness of different batches of E-LiI electrolyte membranes.


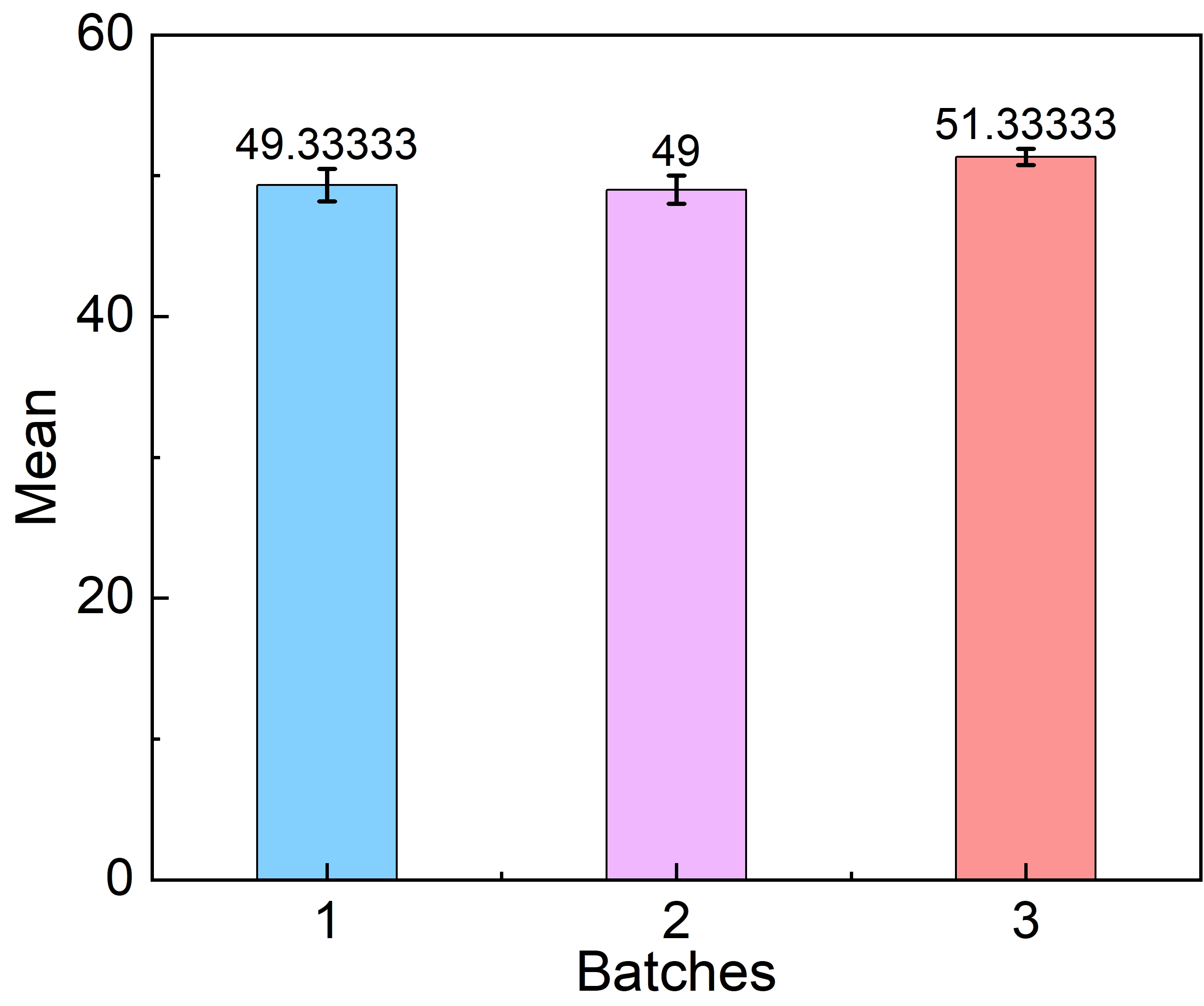


**Figure S15.** Mean thickness of different batches of E-LiI electrolyte membranes.


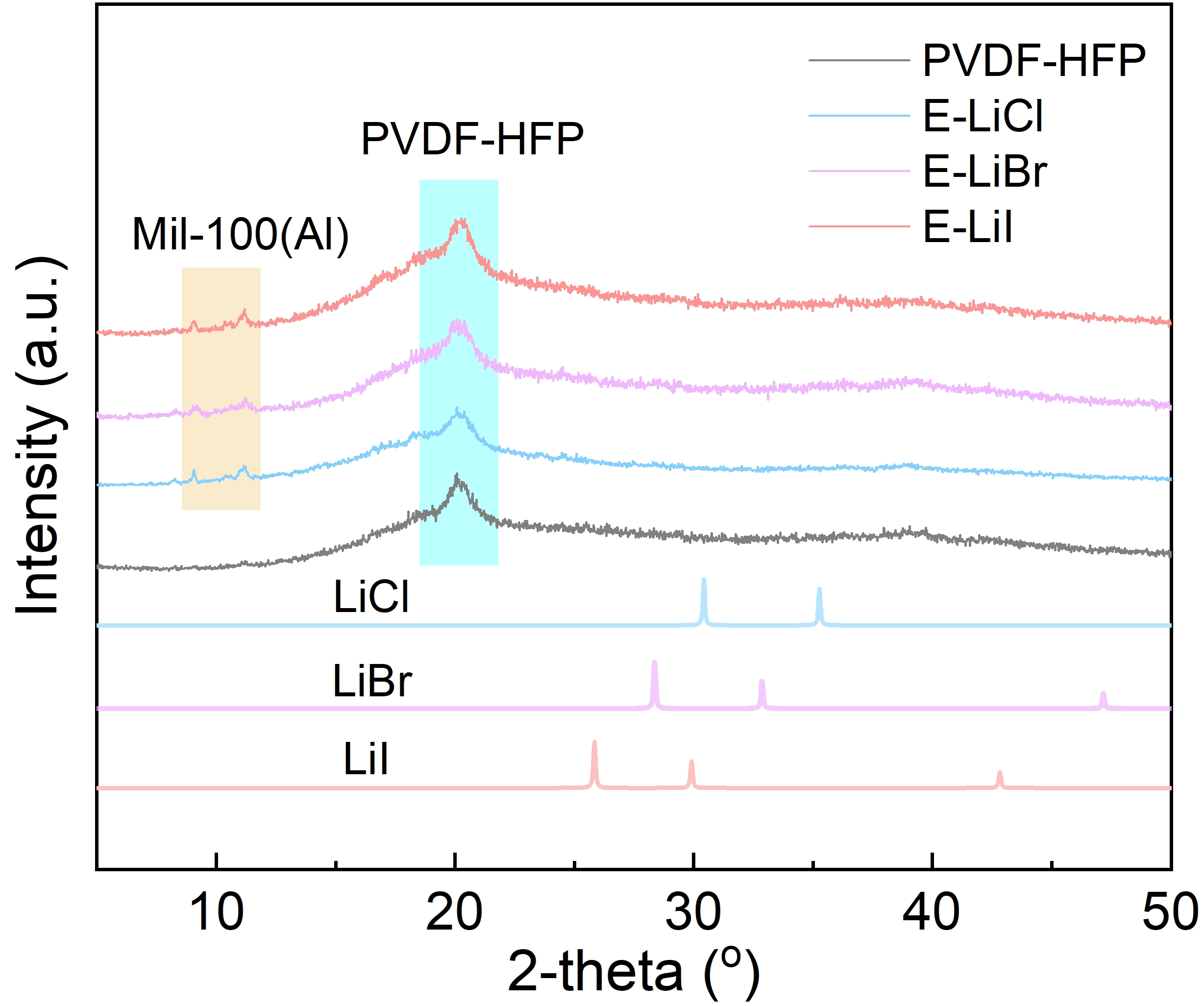


**Figure S16.** XRD pattern of PVDF-HFP, E-LiCl, E-LiBr, E-LiI, and LiX (X=Cl, Br and I) powder.


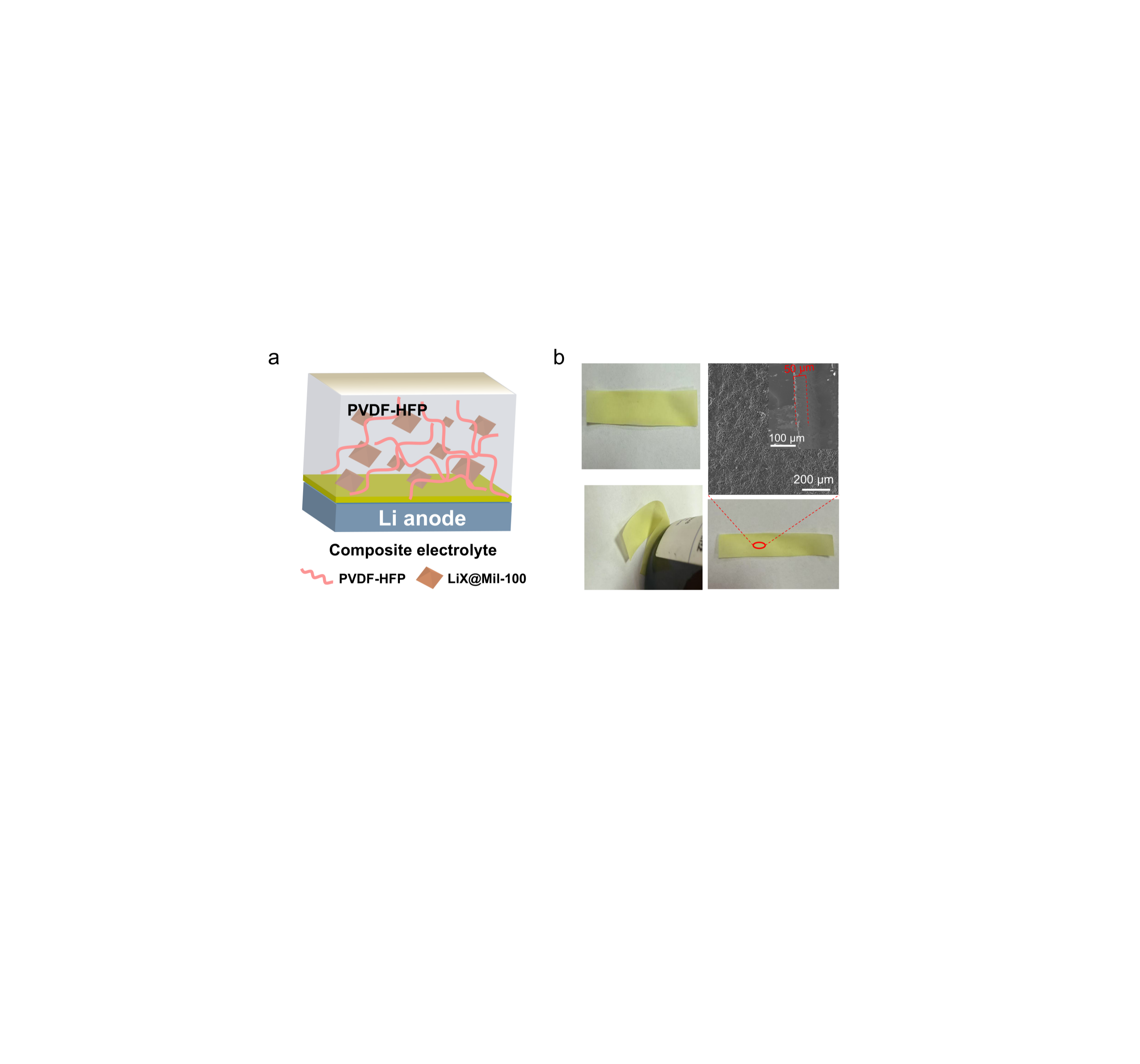


**Figure S17.** (a) Schematic diagram of E-LiX electrolytes. (b) Flexibility of E-LiI electrolyte in digital photos.


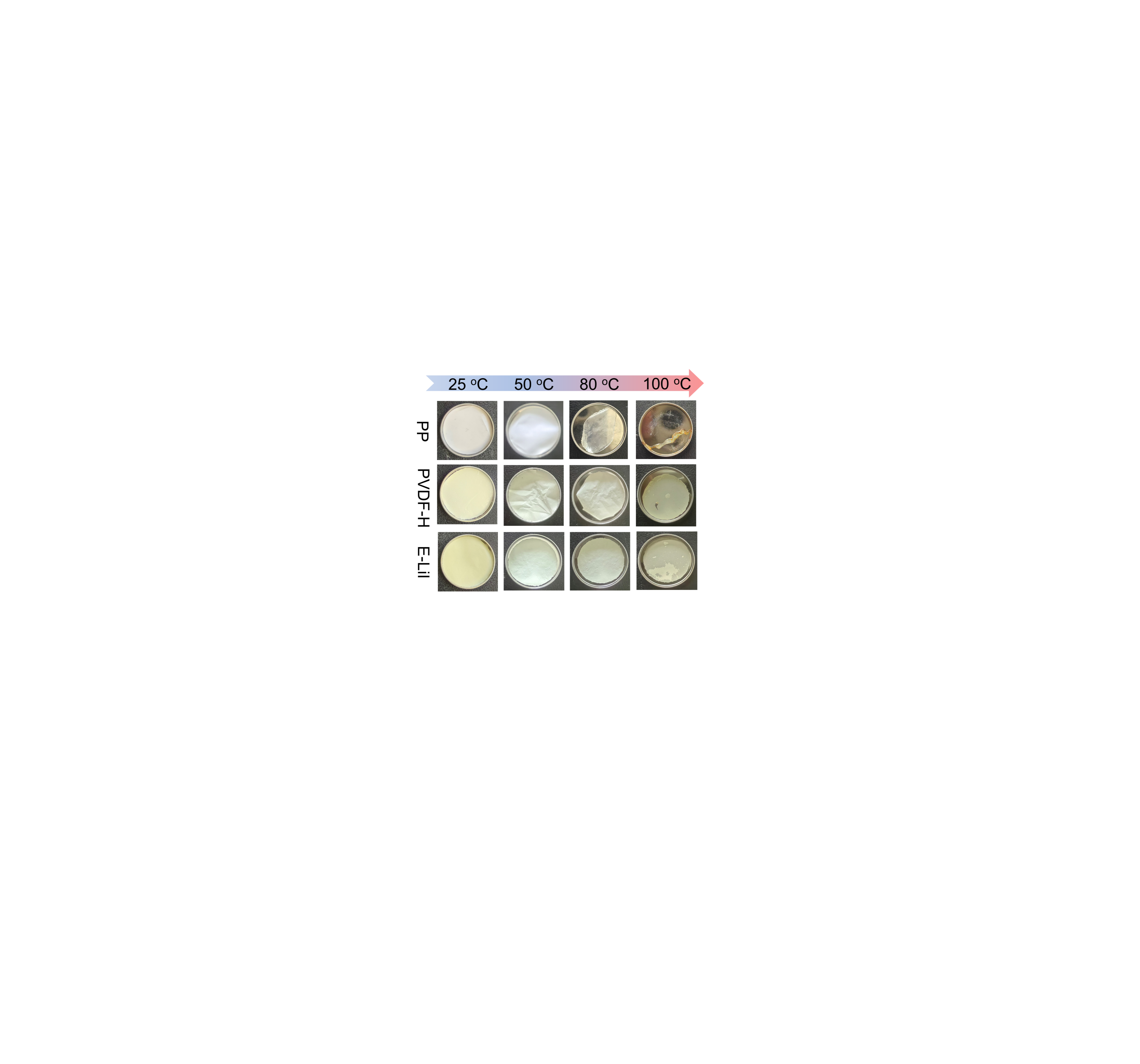


**Figure S18.** Thermal stability of commercial PP separator, PVDF-HFP and E-LiI.

**
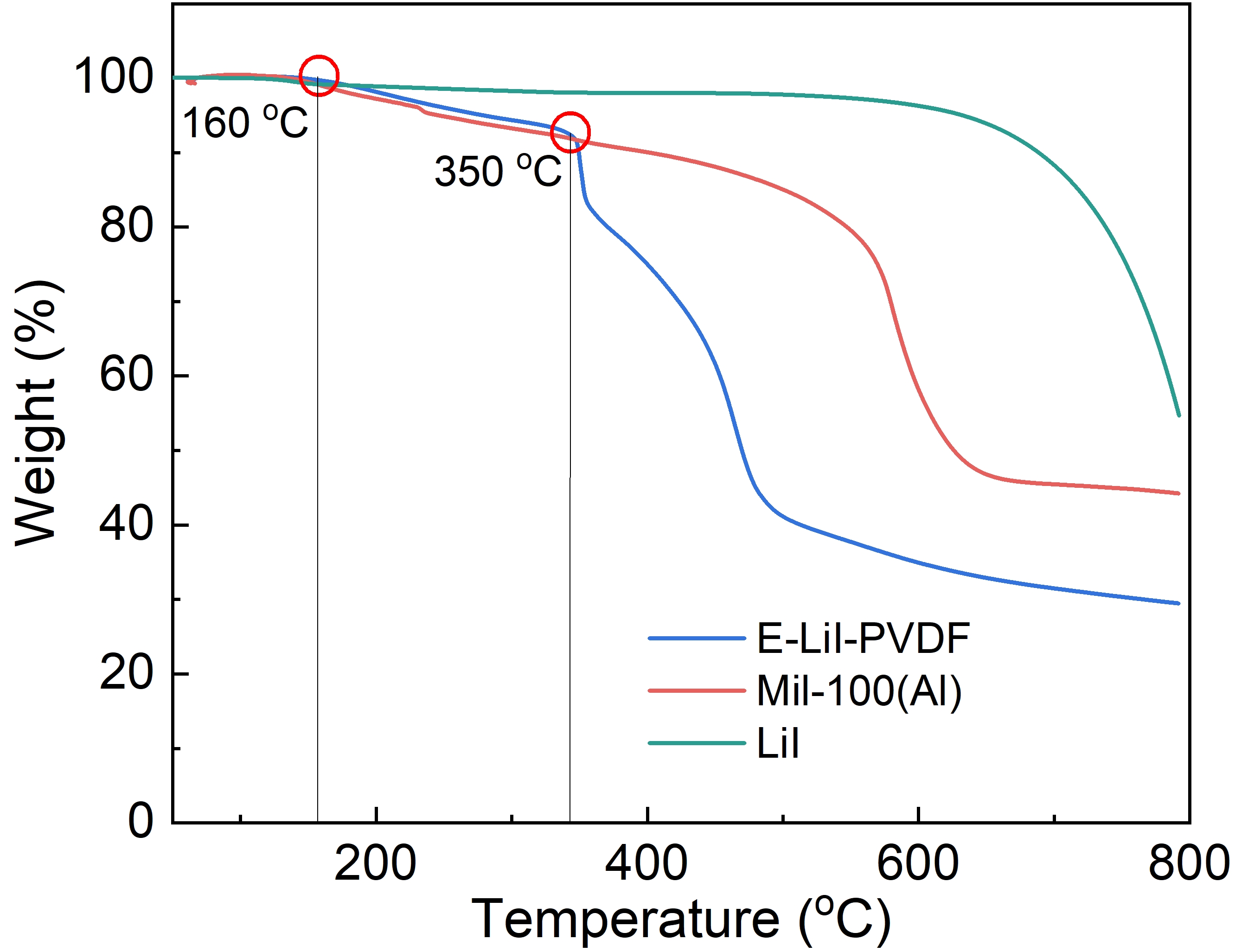
**

**Figure S19.** TGA curves of LiI, Mil-100 and E-LiI-PVDF.

**
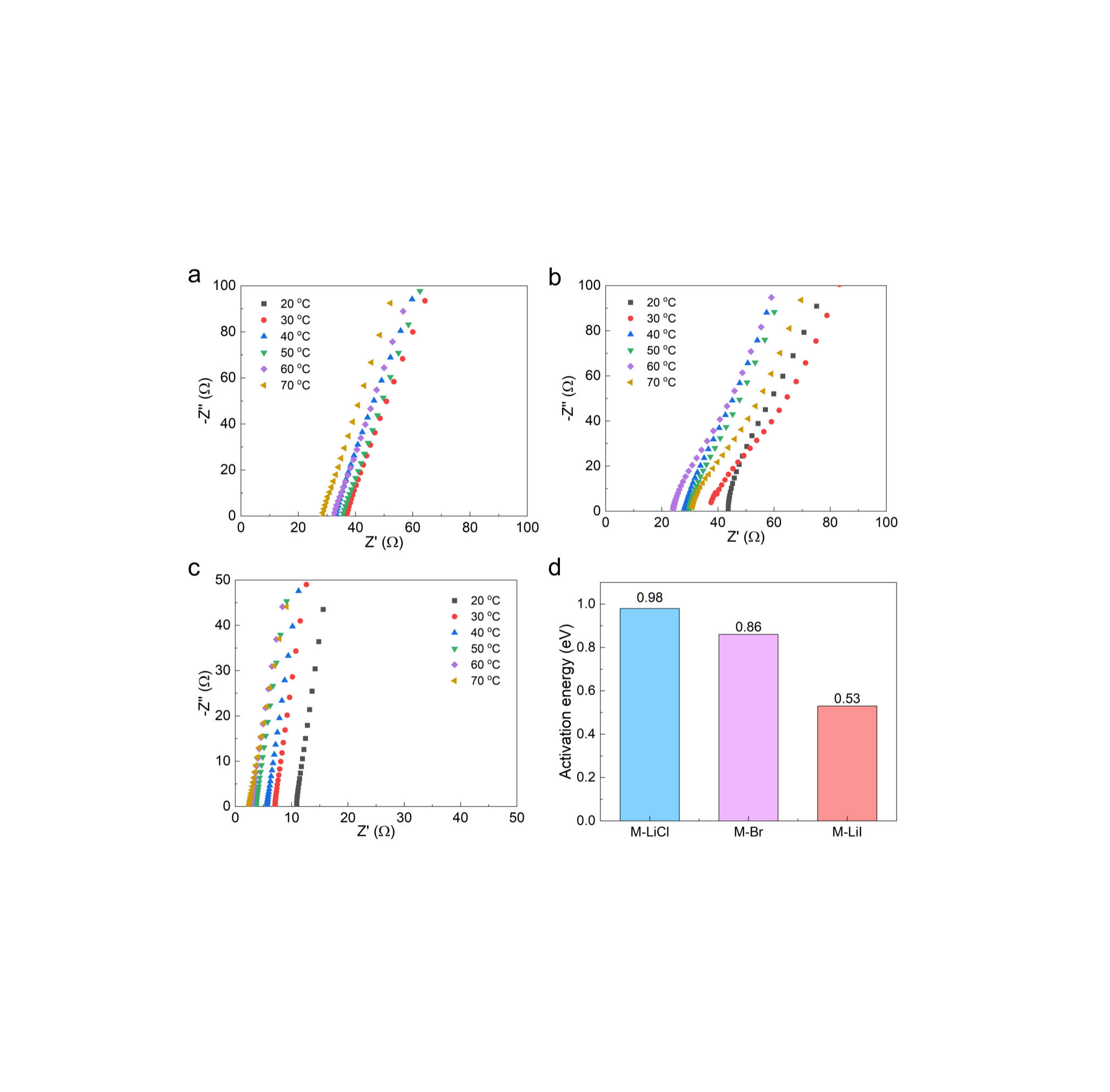
**

**Figure S20.** EIS of SS//SS cells at different temperatures using the (a) E-LiCl, (b)E-LiBr and (C) E-LiI. (d) Activation energy (E_a_) calculation of E-LiCl, E-LiBr and E-LiI.

**
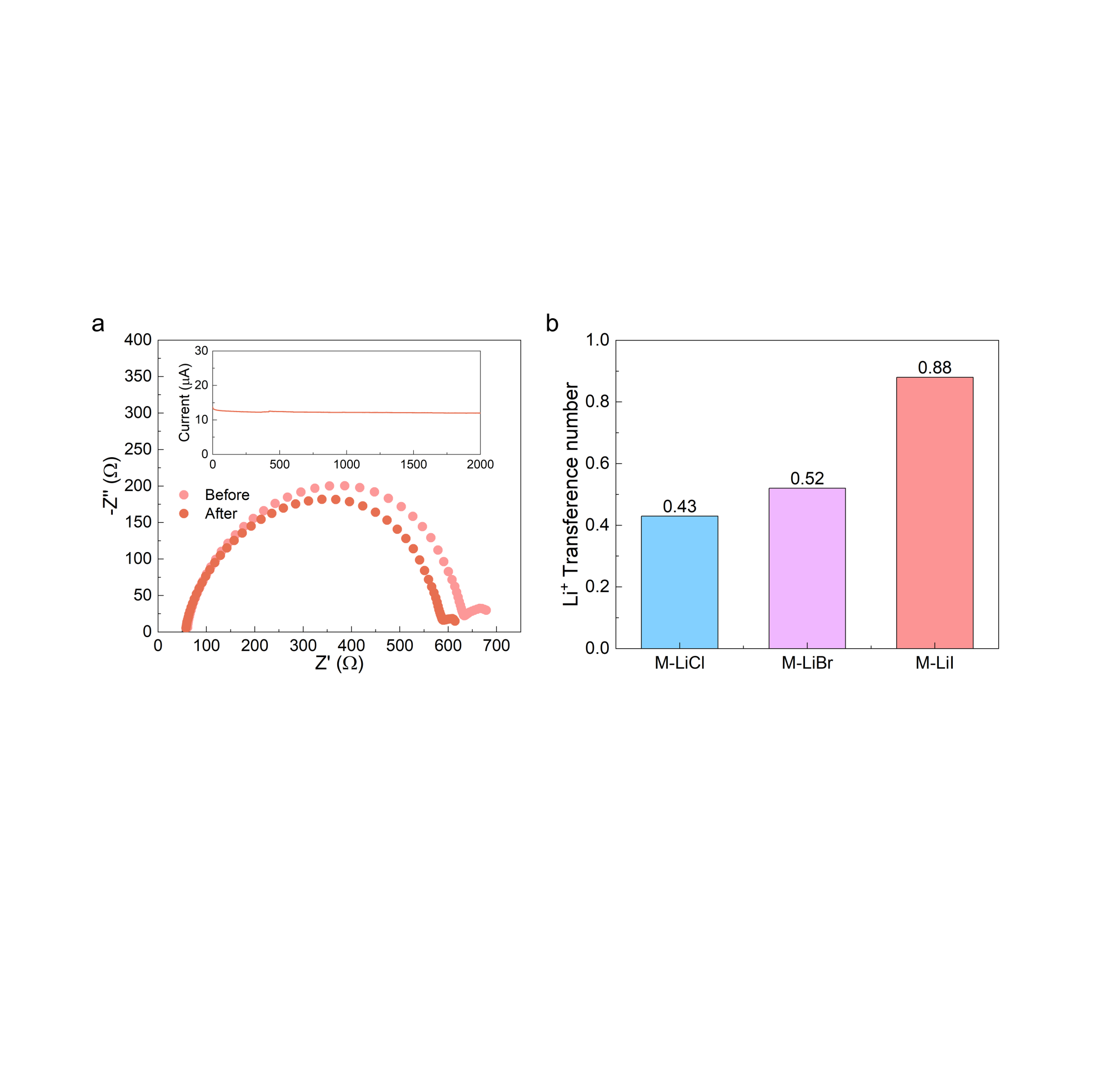
**

**Figure S21.** Current-time curves of the Li/different electrolyte/Li symmetric batteries with a voltage of 10 mV and the inset shows the electrochemical impedance spectroscopy of the battery before and after polarization at 25 ^o^C.

**
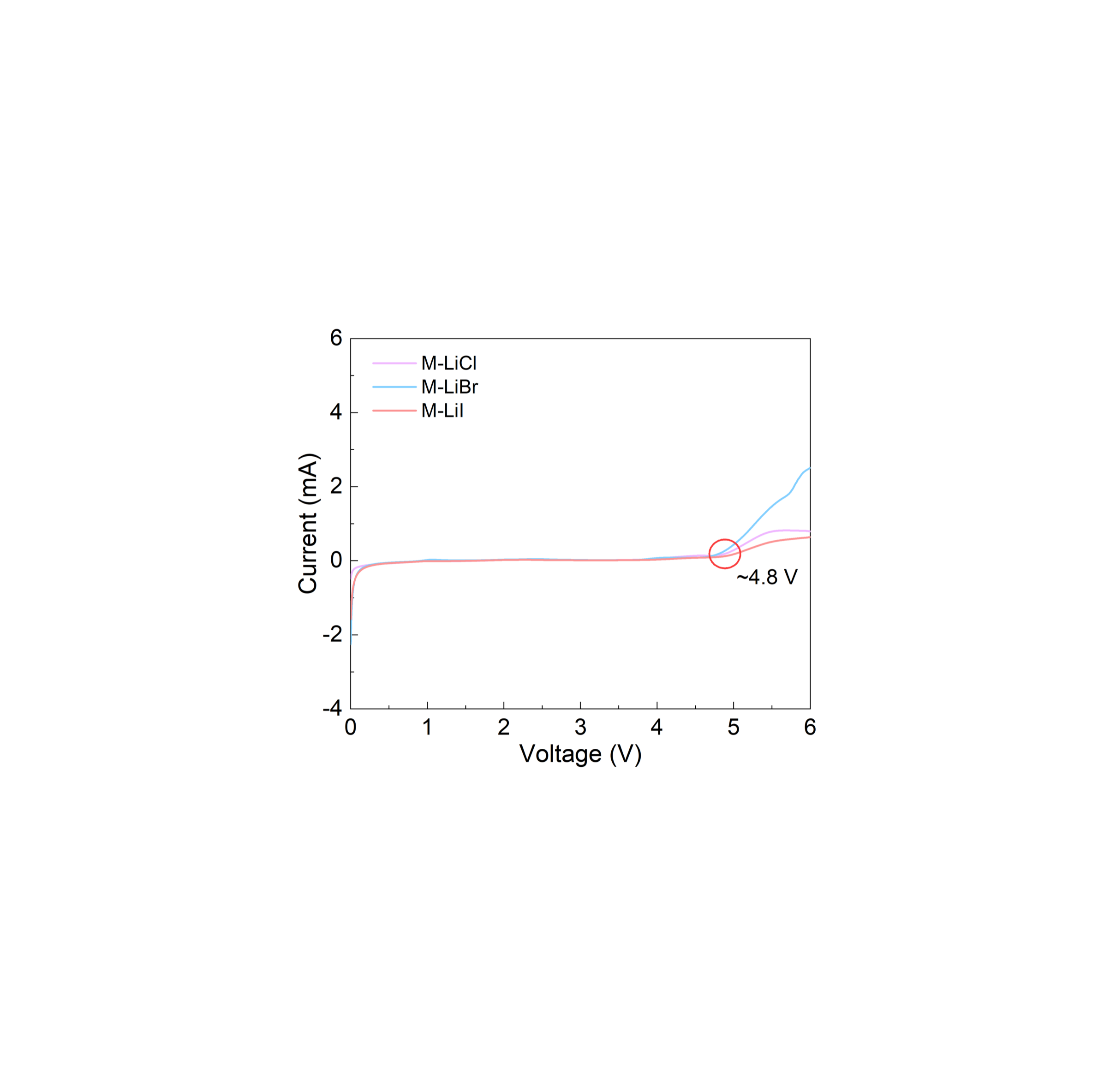
**

**Figure S22.** LSV curves of the E-LiCl, E-LiBr and E-LiI.

**
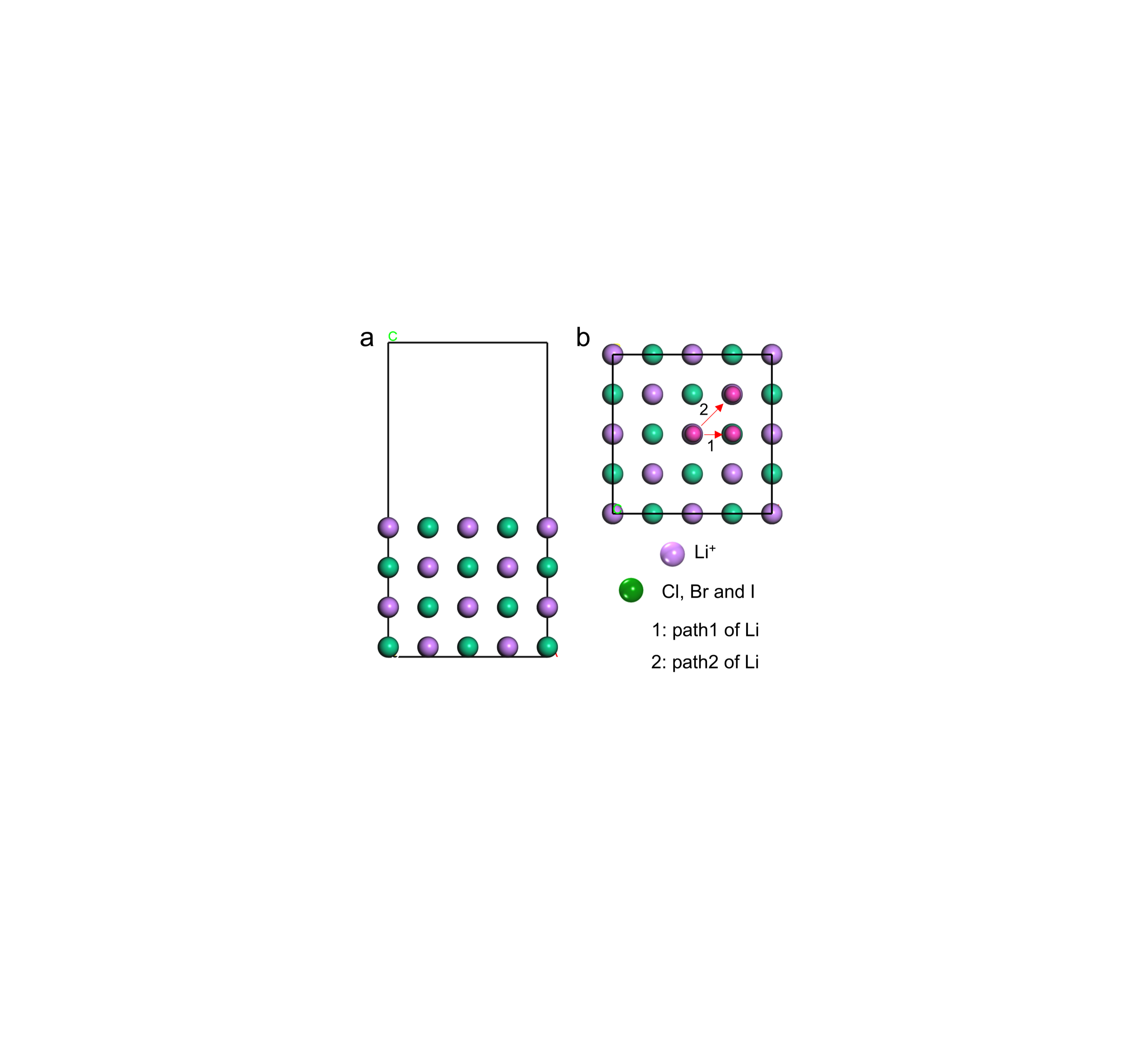
**

**Figure S23.** (a) Side view and (b) top view of LiX and two diffusion paths of Li+.

**
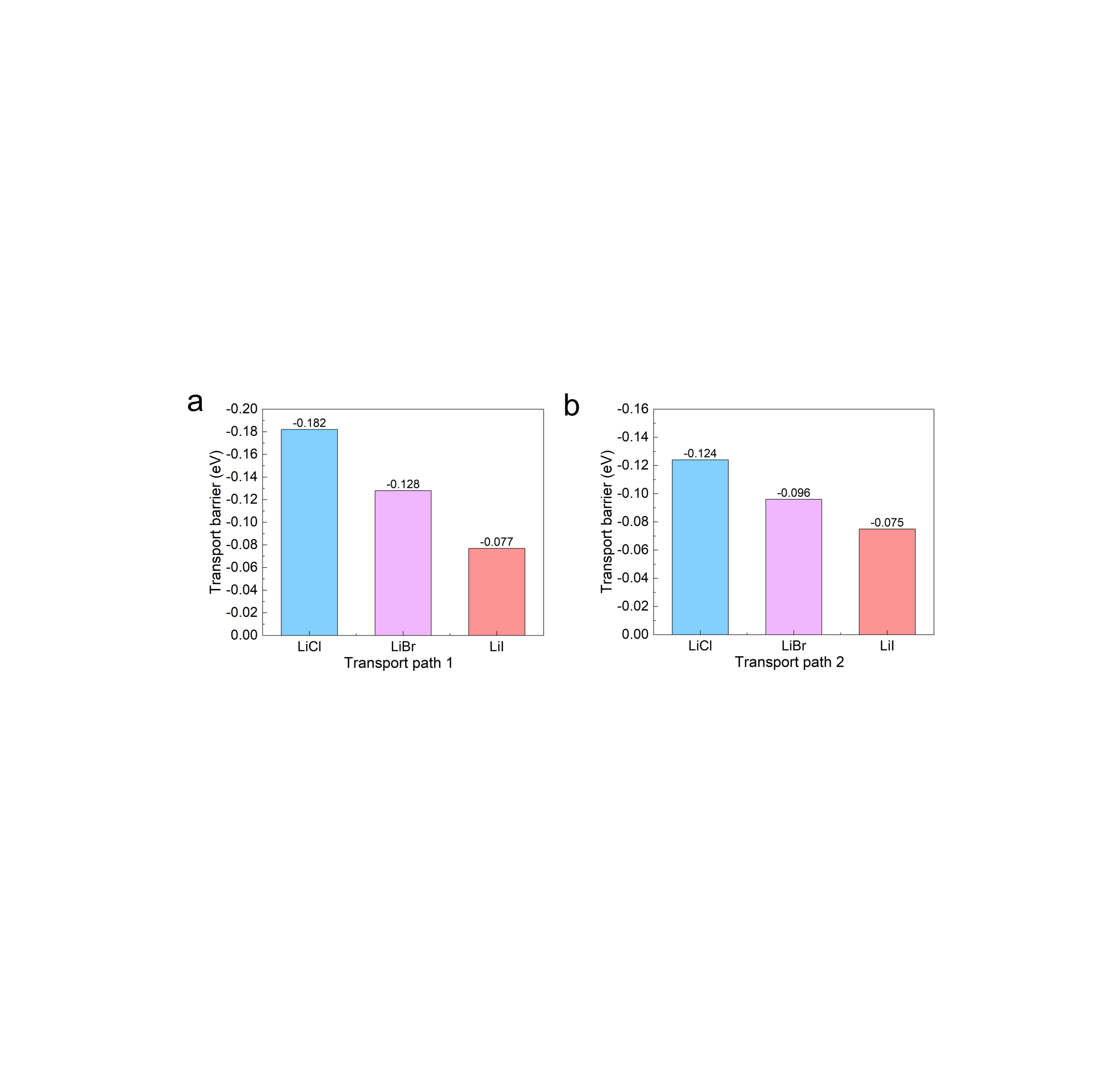
**

**Figure S24.** Energy to be overcome for Li transport in different transport paths on LiCl, LiBr and LiI.

**
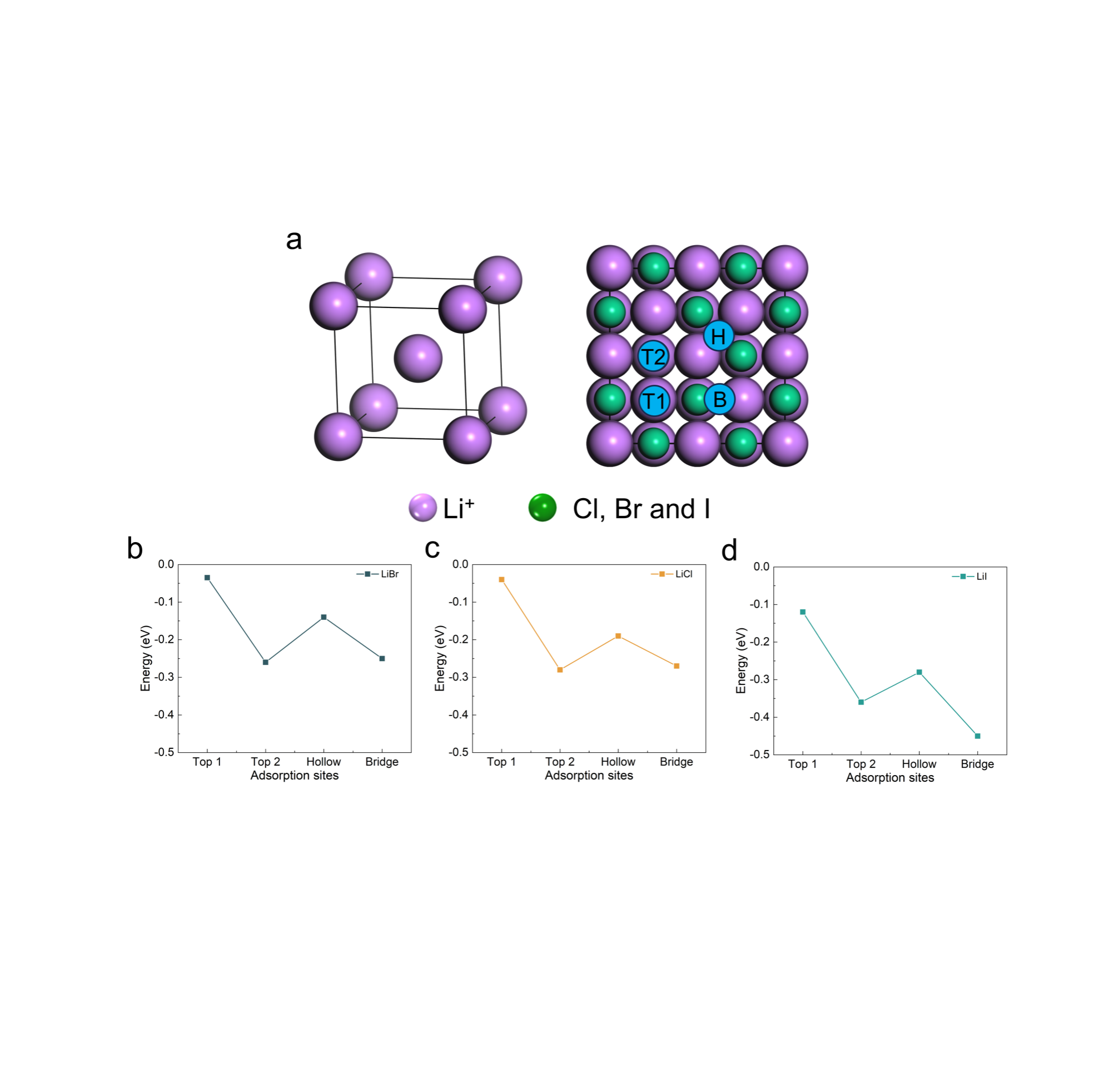
**

**Figure S25.** (a) Li metal cell and the schematic diagram of different adsorption sites on the surface of LiX. Adsorption energies of Li at different sites on the surfaces of (b) LiBr, (c) LiCl, and (d) LiI.

**
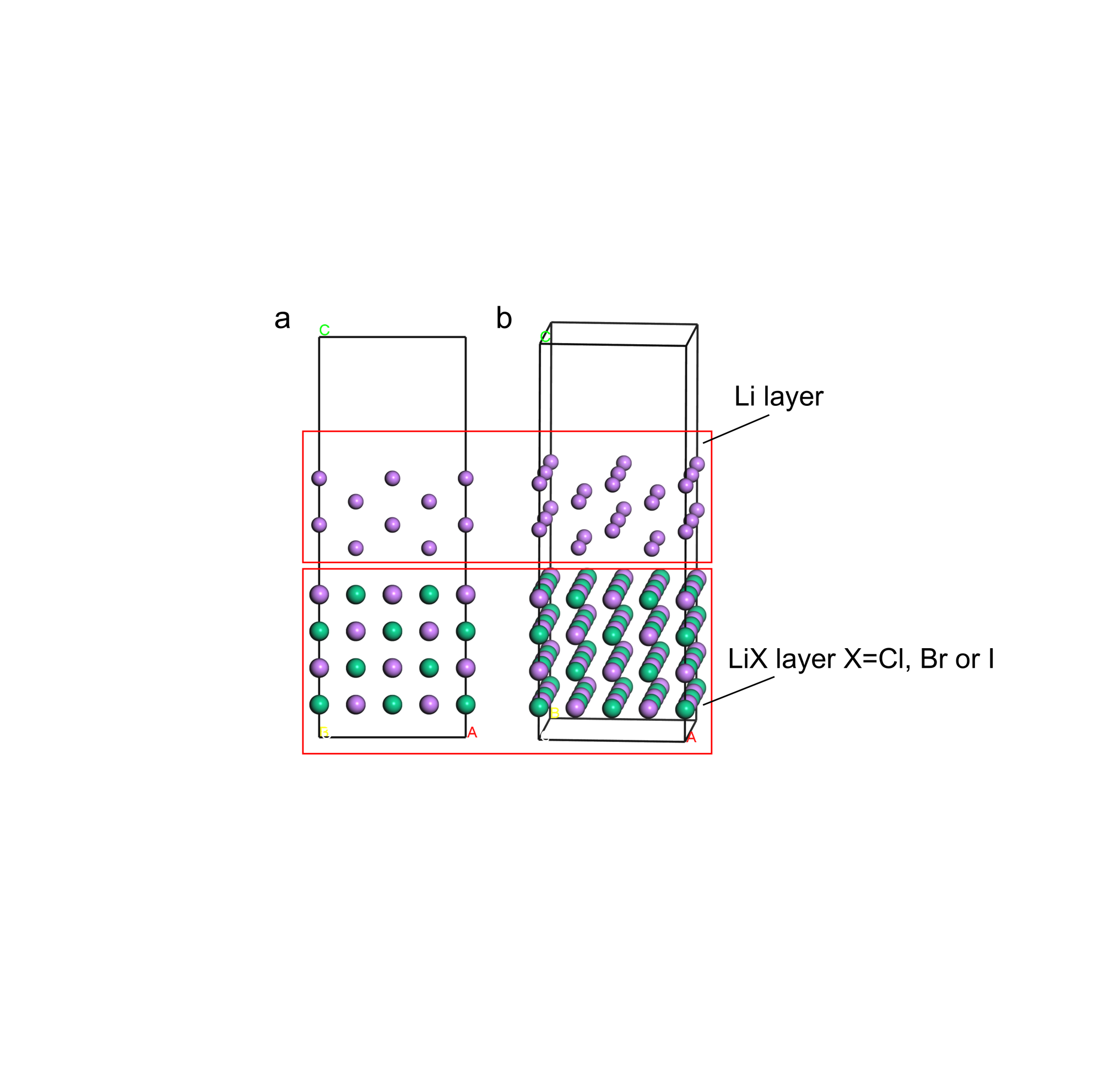
**

**Figure S26.** The interface model of Li/LiX: (a) Front view and (b) side view.

**
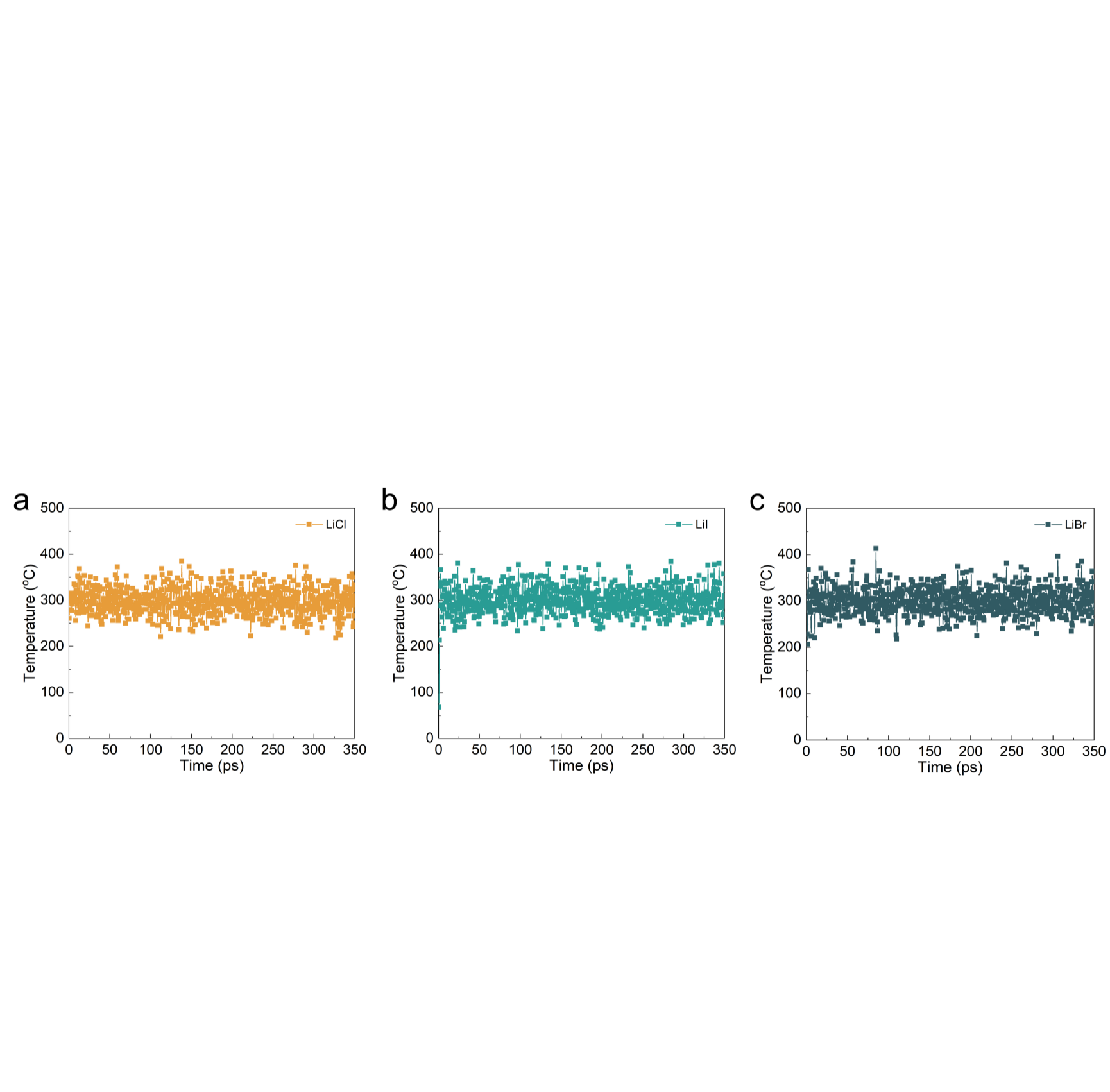
**

**Figure S27.** Curves of the temperature versus time separately of Li/LiX.

**
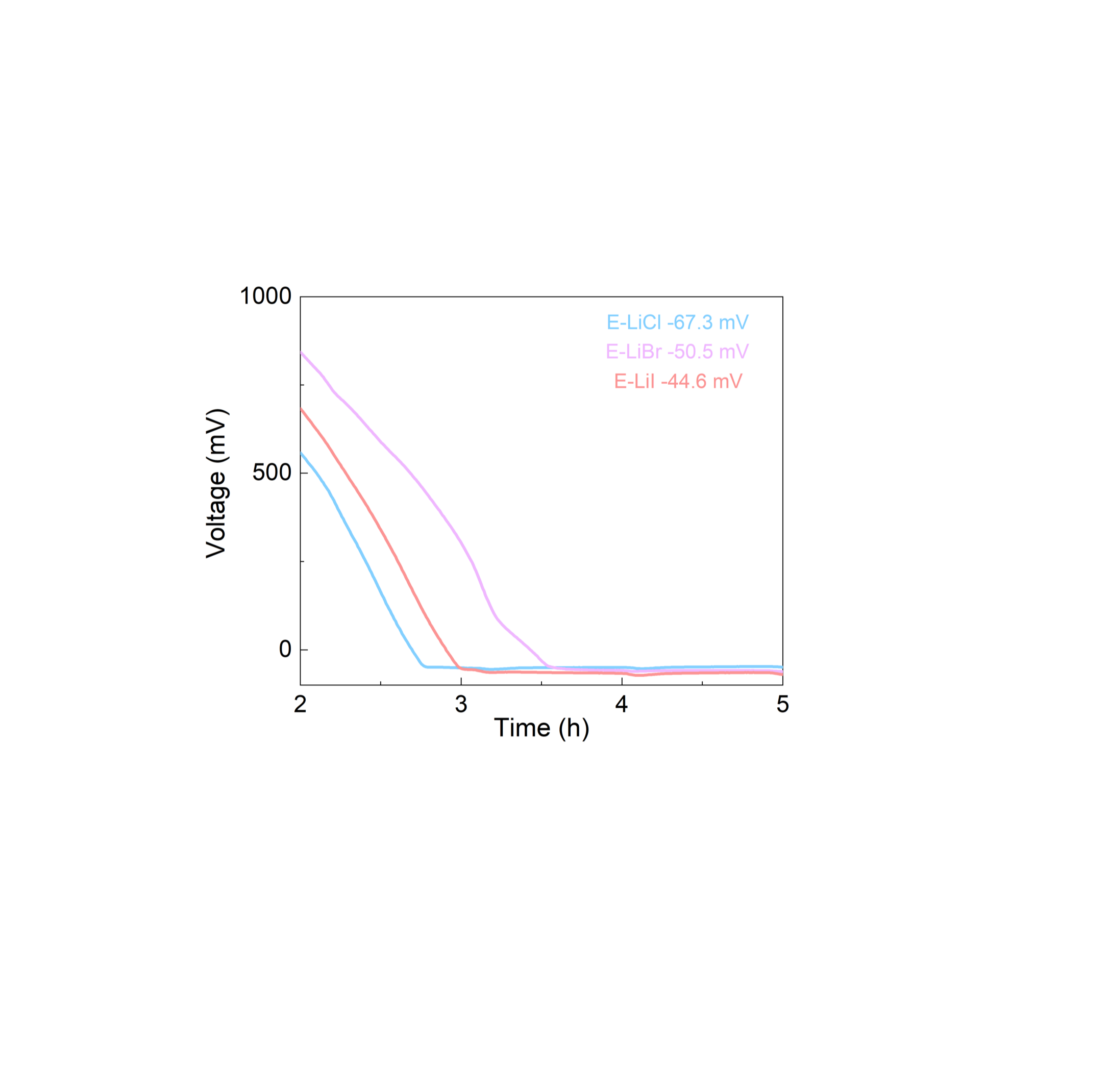
**

**Figure S28.** Nucleation potential curves of Li//Cu half-cells assembled with E-LiCl, E-LiBr and E-LiI.

**
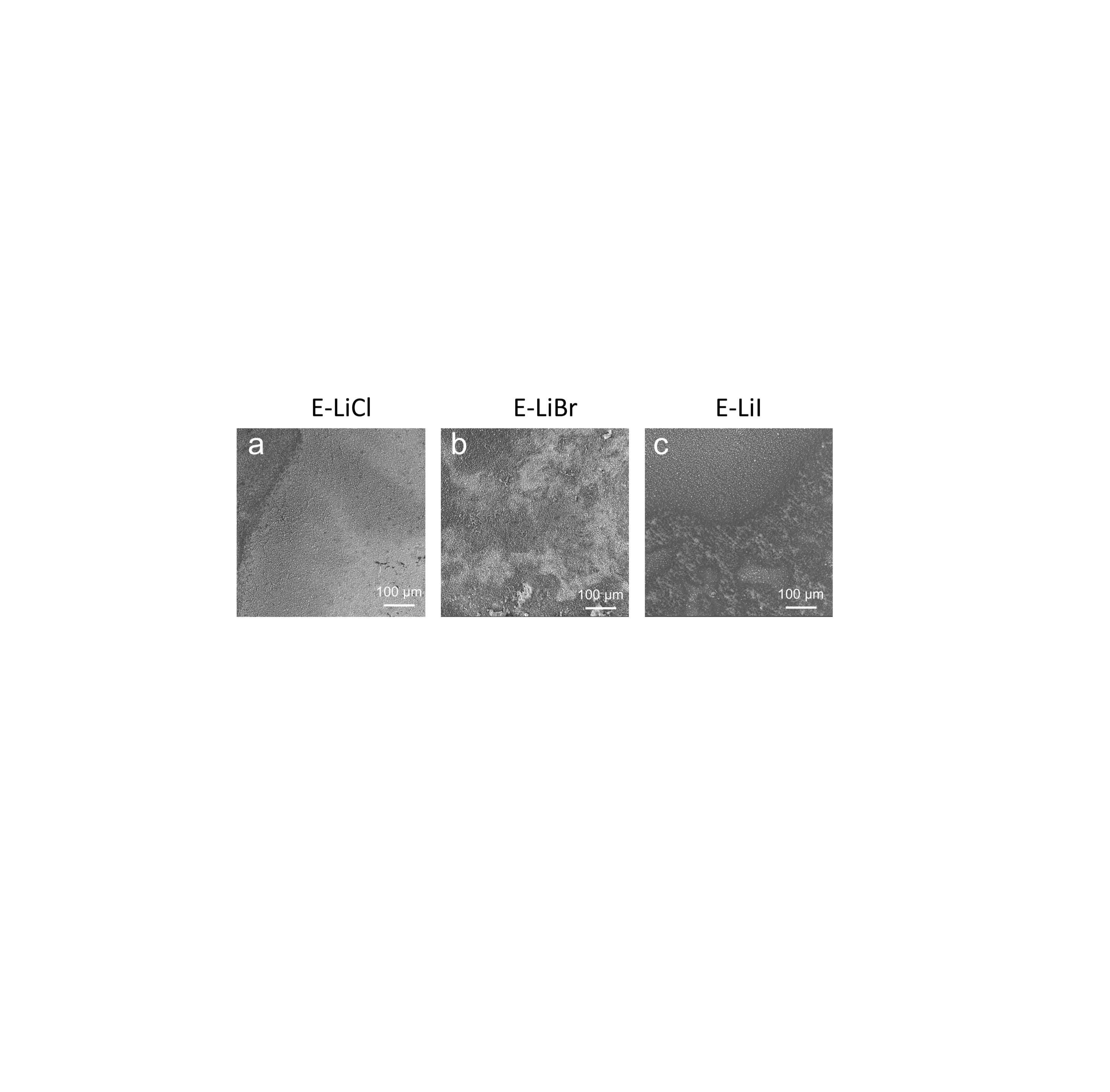
**

**FigureS29.** The SEM images on the surface of the Cu foils after the nucleation experiments.

**
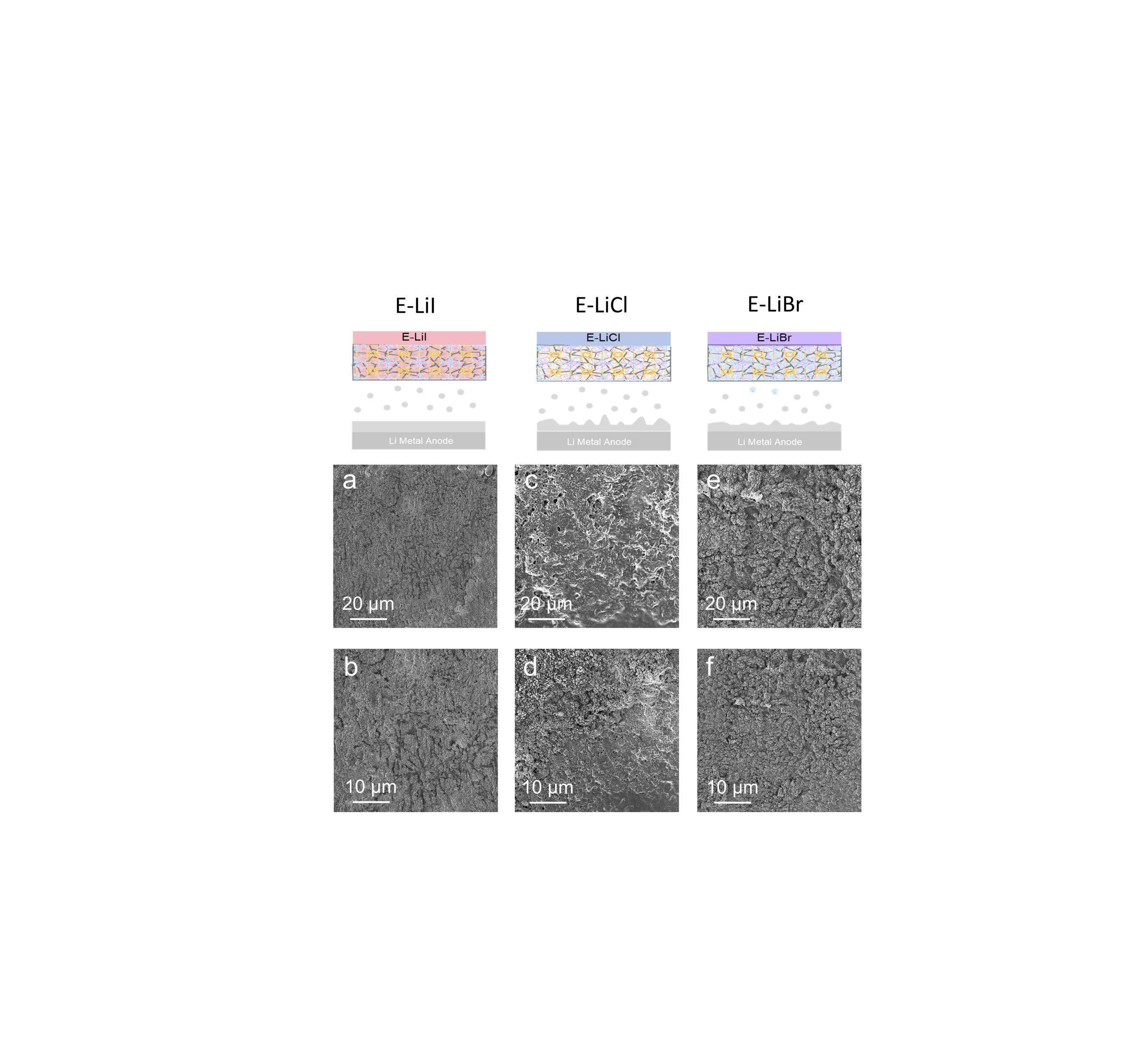
**

**Figure S30.** SEM images of lithium metal surface after plating/stripping with different electrolytes.

**
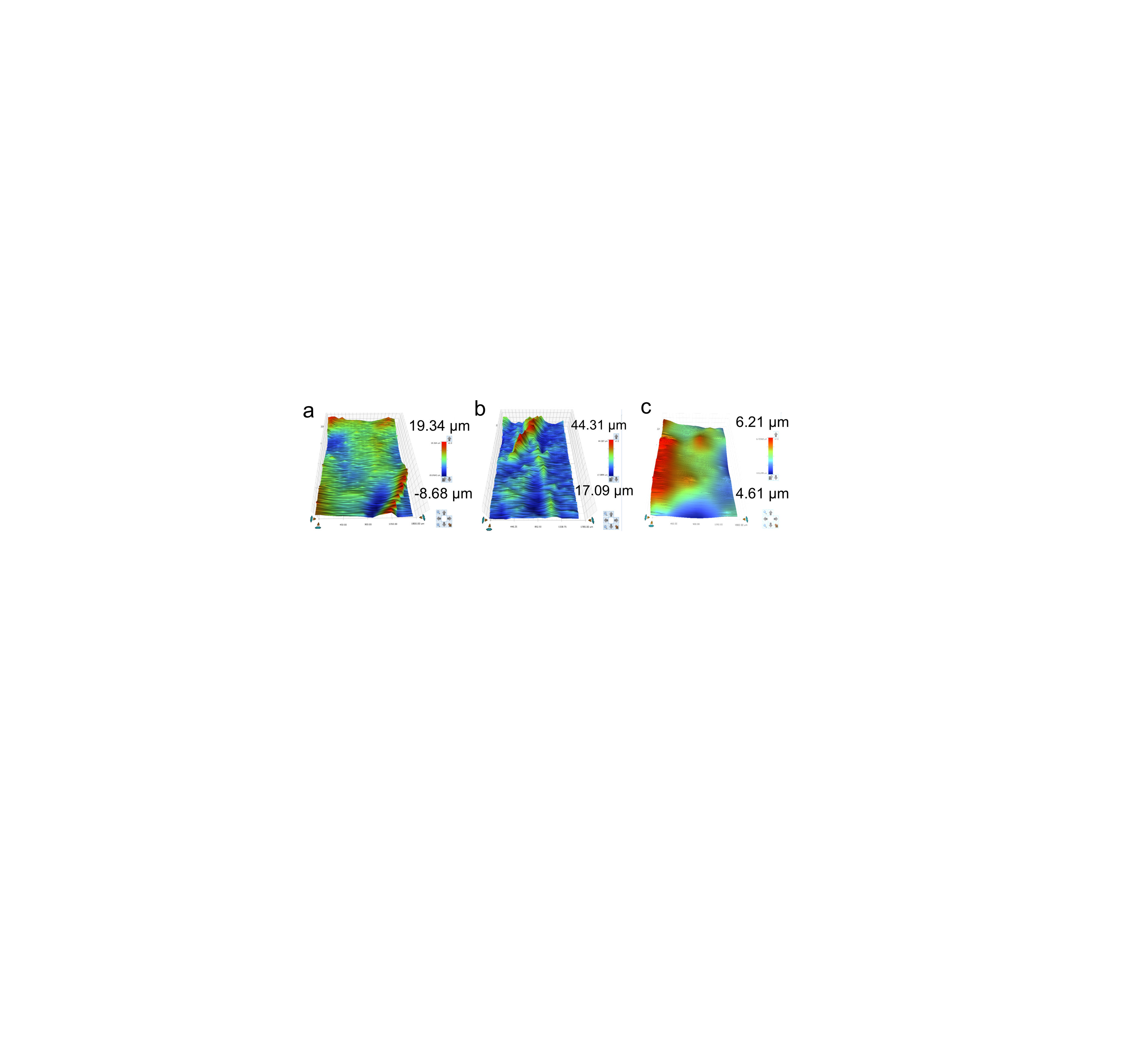
**

**Figure S31.** Step profiler tests on Li metal surfaces of (a) Li||E-LiCl||Li, (b) Li||E-LiBr||Li and (c) Li||E-LiI||Li symmetric cells.

**
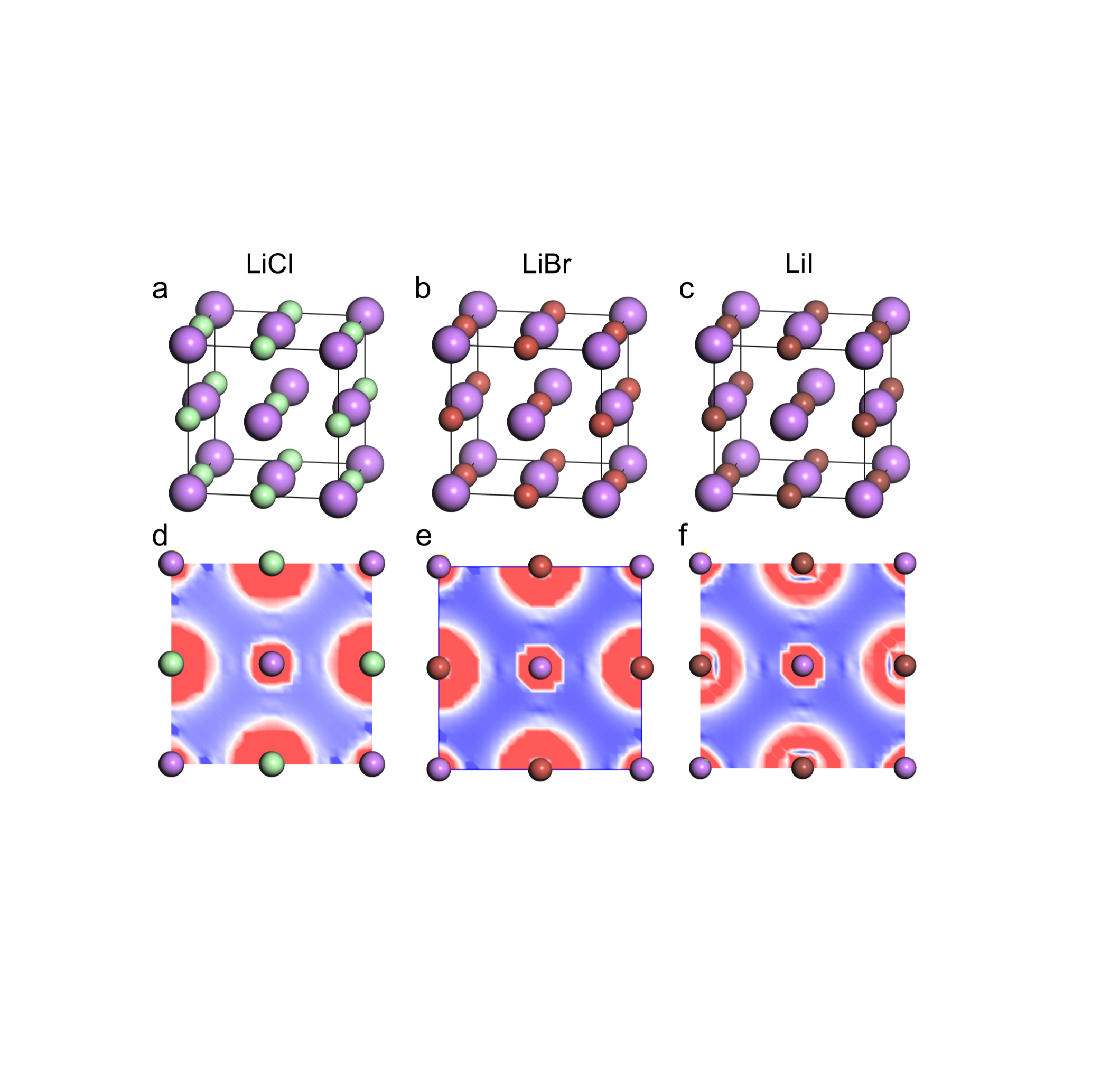
**

**Figure S32.** (a-c) Crystal protocells of LiCl, LiBr and LiI, and (d-f) the corresponding charge distributions.

**
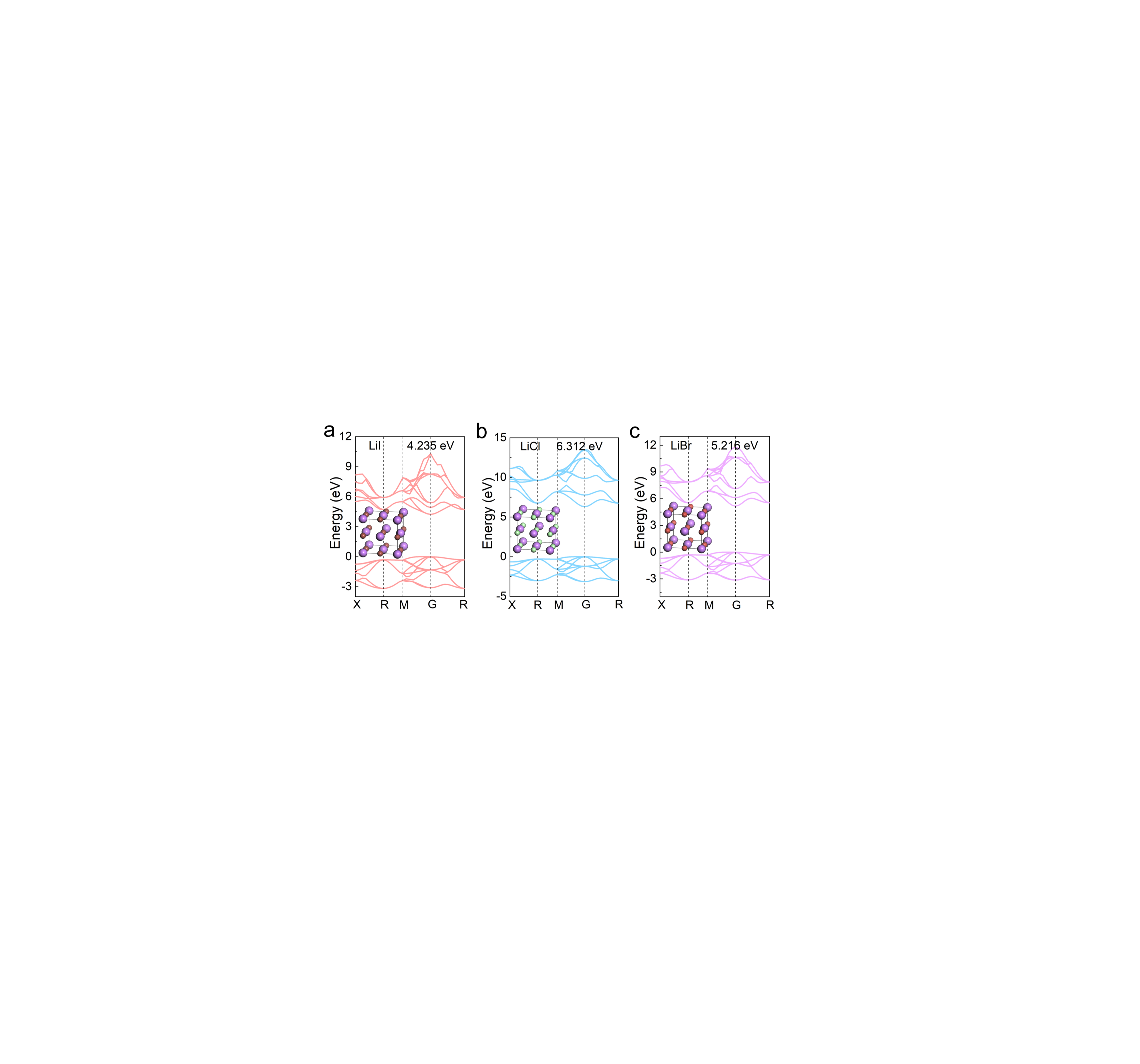
**

**Figure S33.** (a-b) Energy bands of LiI, LiCl and LiBr.

**
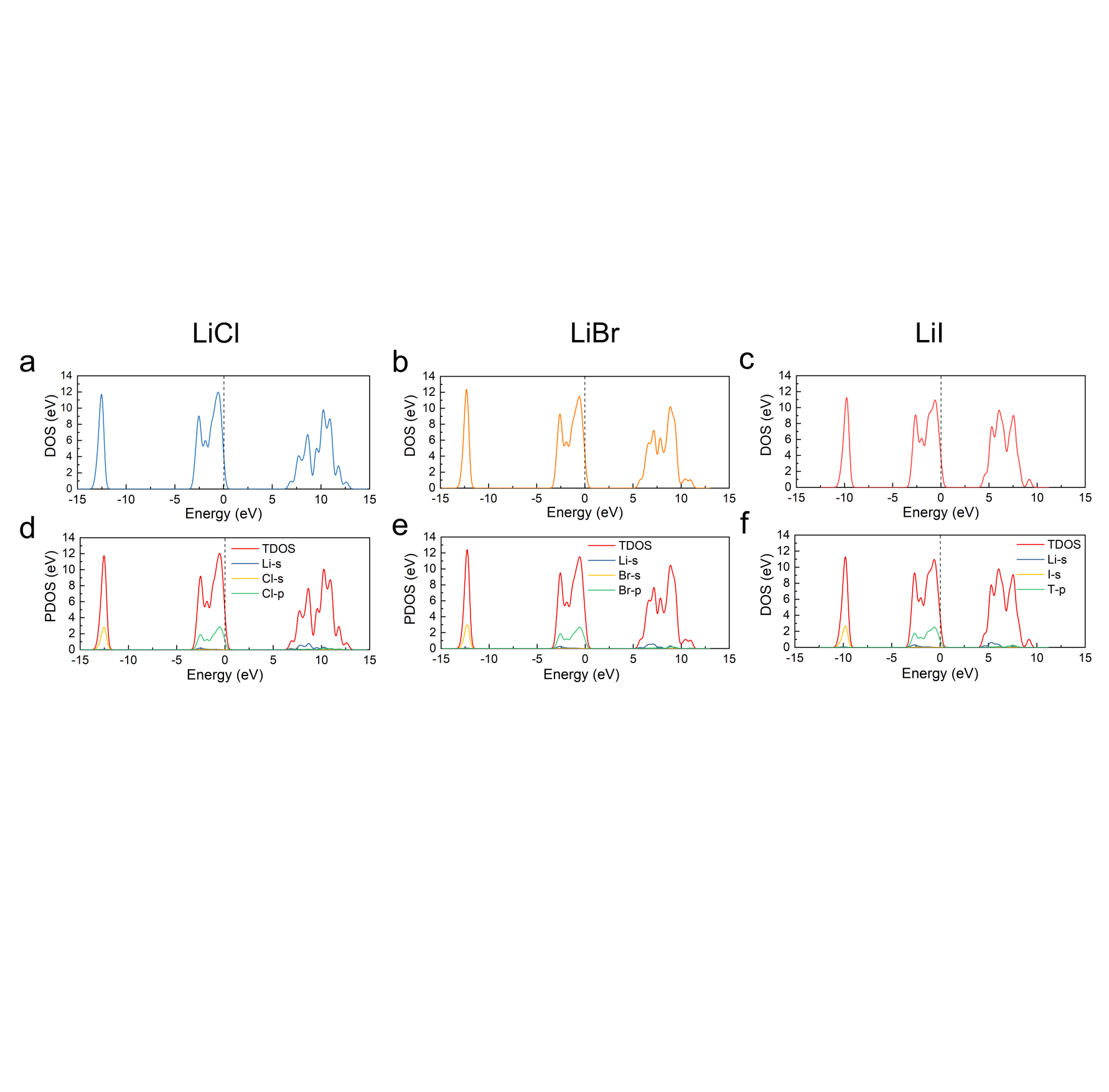
**

**Figure S34.** Density of States (DOS) and Partial Density of States (PDOS) of (a,d) LiCl, (b,e) LiBr, and (c,f) LiI.


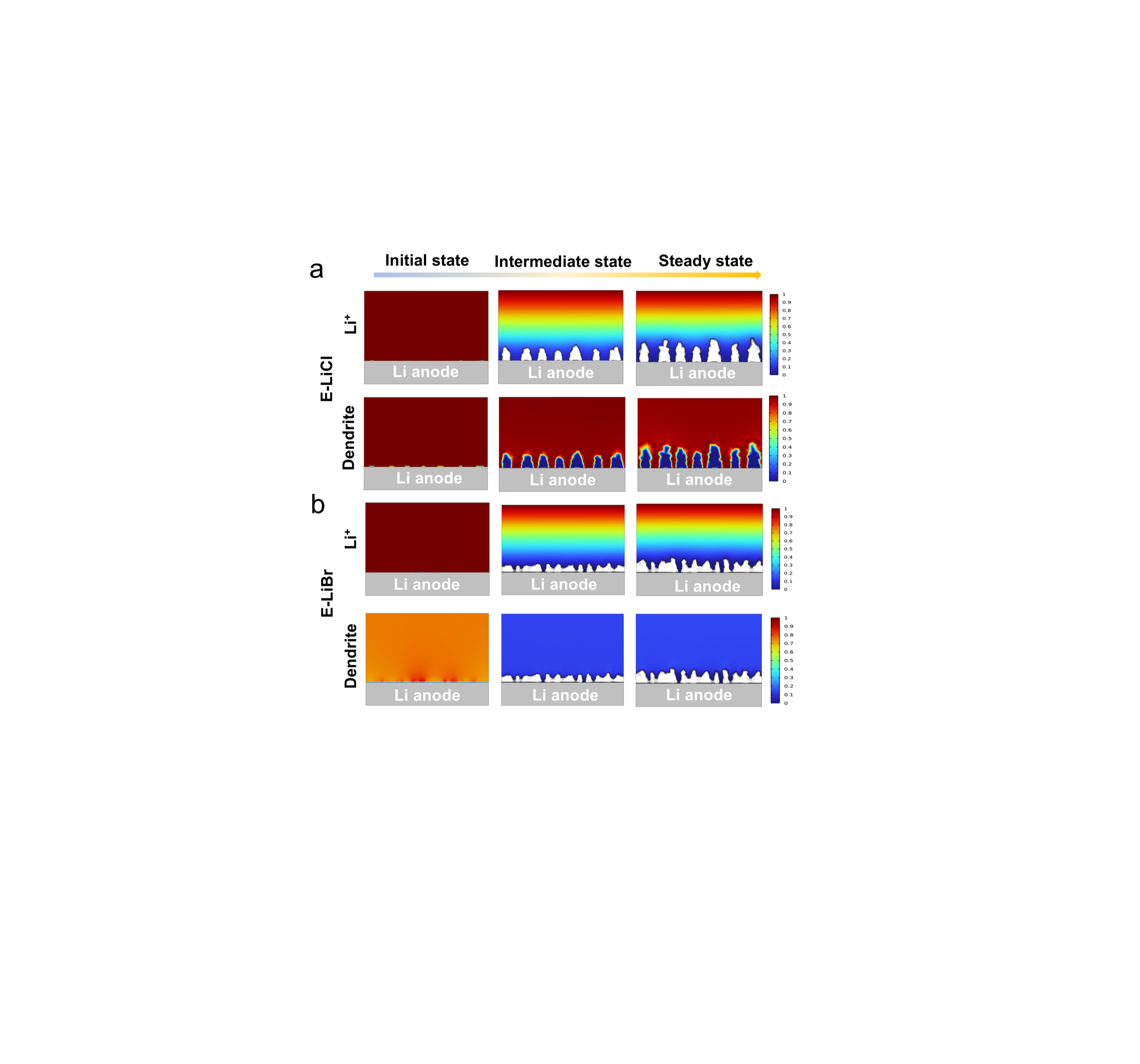


**Figure S35.** Distribution for Li^+^ concentration and Li-deposition of the (a) E-LiCl and (b) E-LiBr in different states based on COMSOL simulation analysis.

**
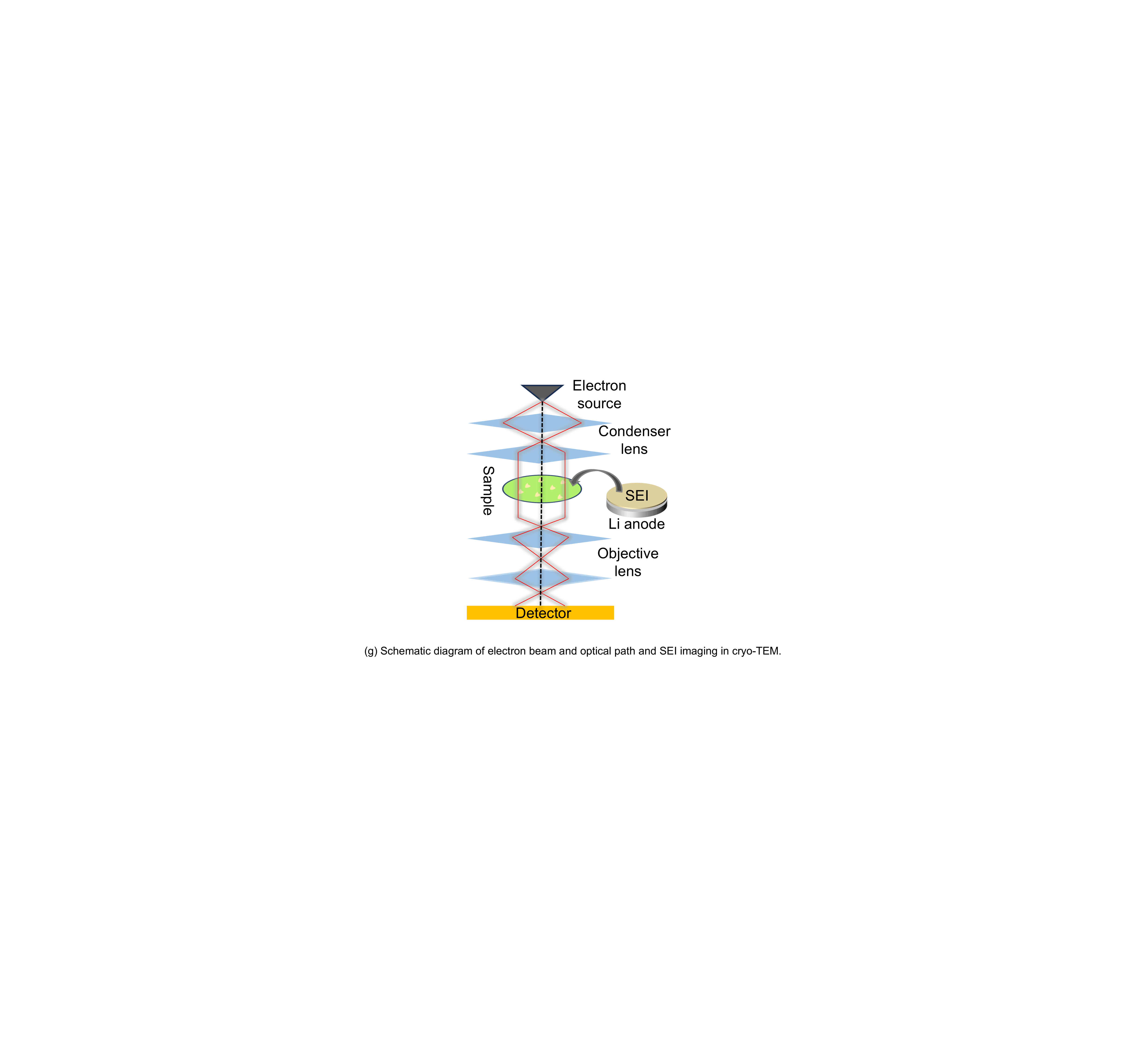
**

**Figure S36.** Schematic diagram of electron beam and optical path and SEI imaging in cryo-TEM.

**
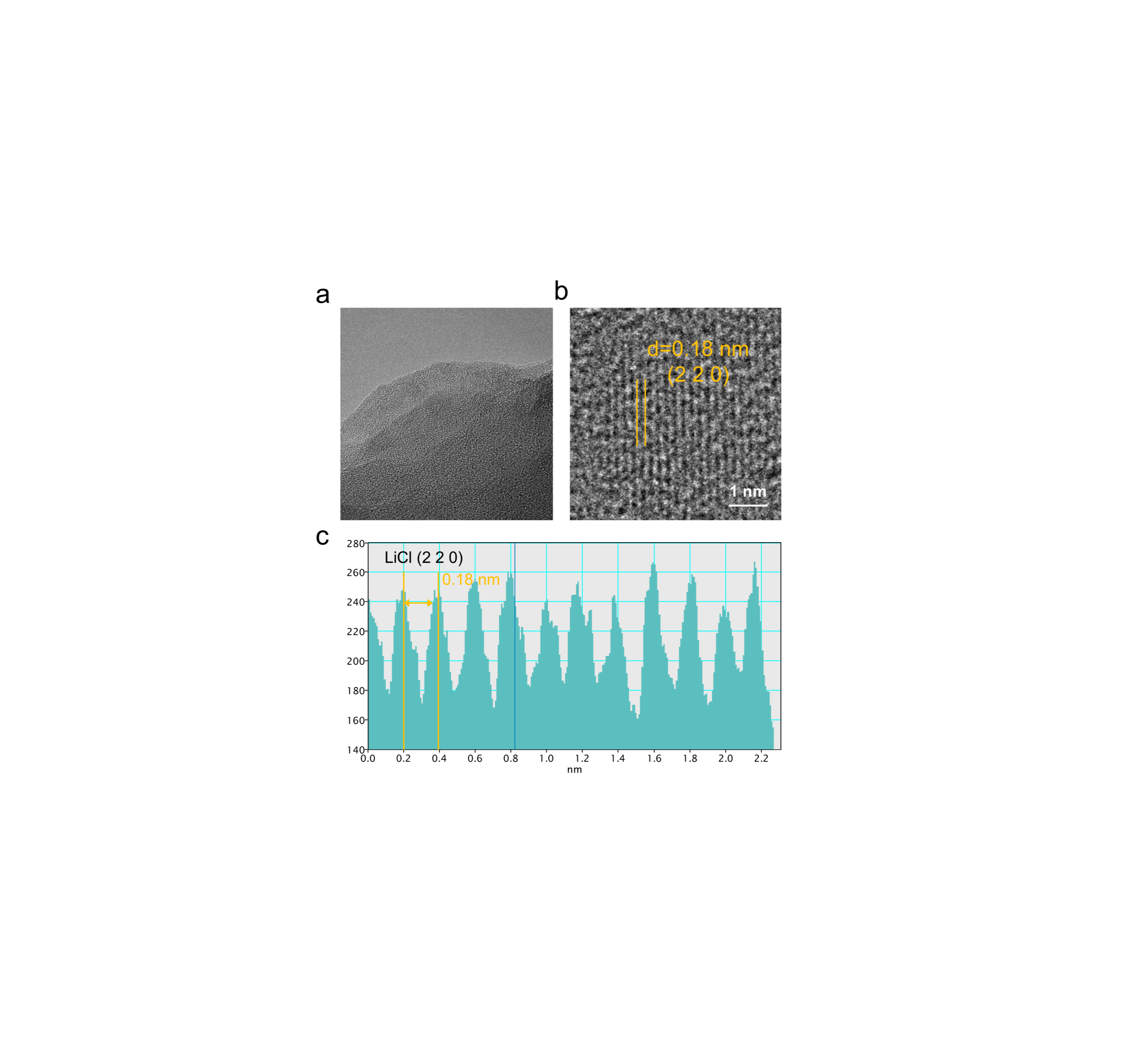
**

**Figure S37.** Cryo-TEM characterization of SEI between E-LiCl and Li anode surface, (a) TEM image, (b) HRTEM image, yellow: LiCl, and corresponding FFT.

**
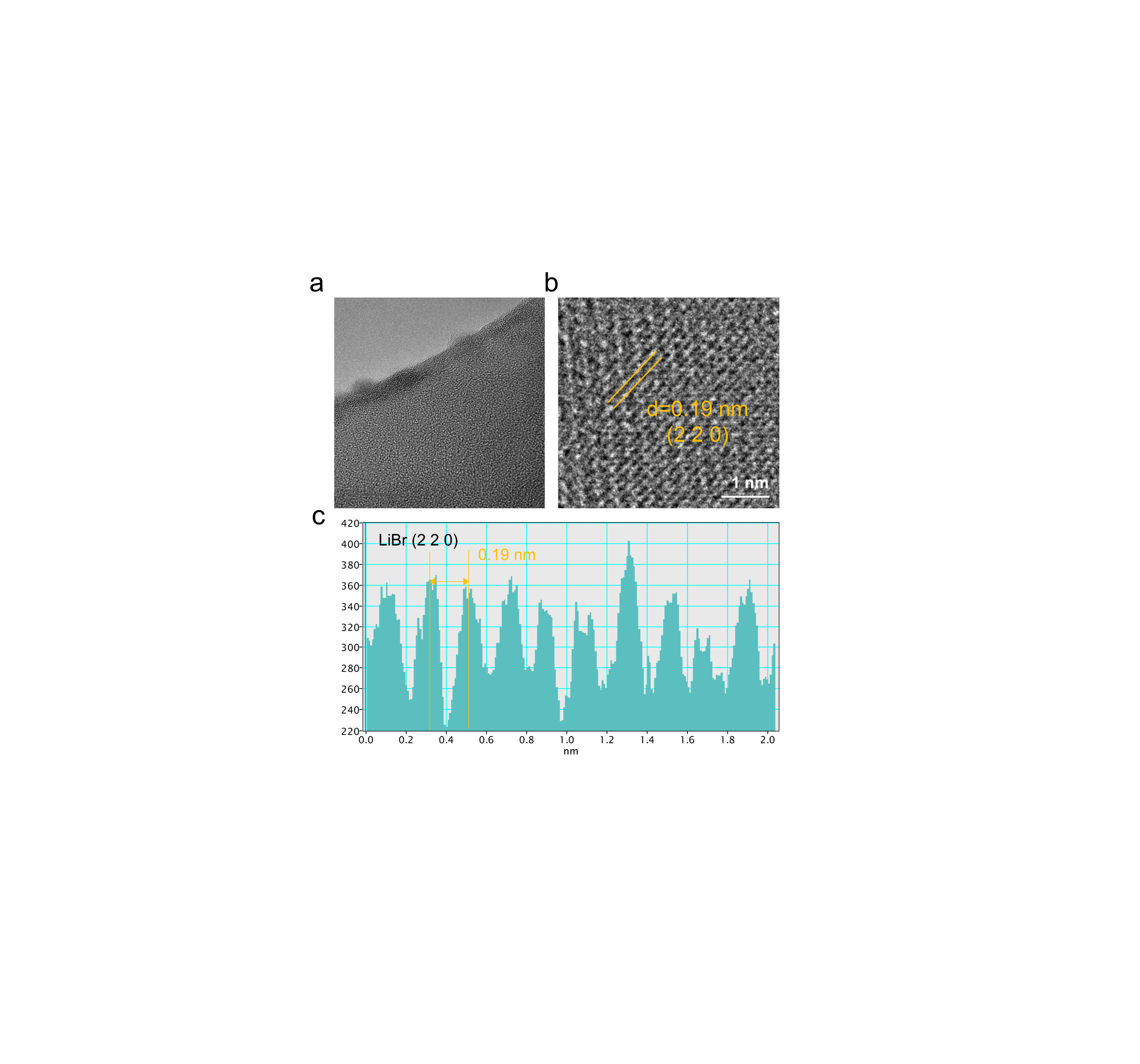
**

**Figure S38.** Cryo-TEM characterization of SEI between E-LiBr and Li anode surface, (a) TEM image, (b) HRTEM image, yellow: LiBr, and corresponding FFT.


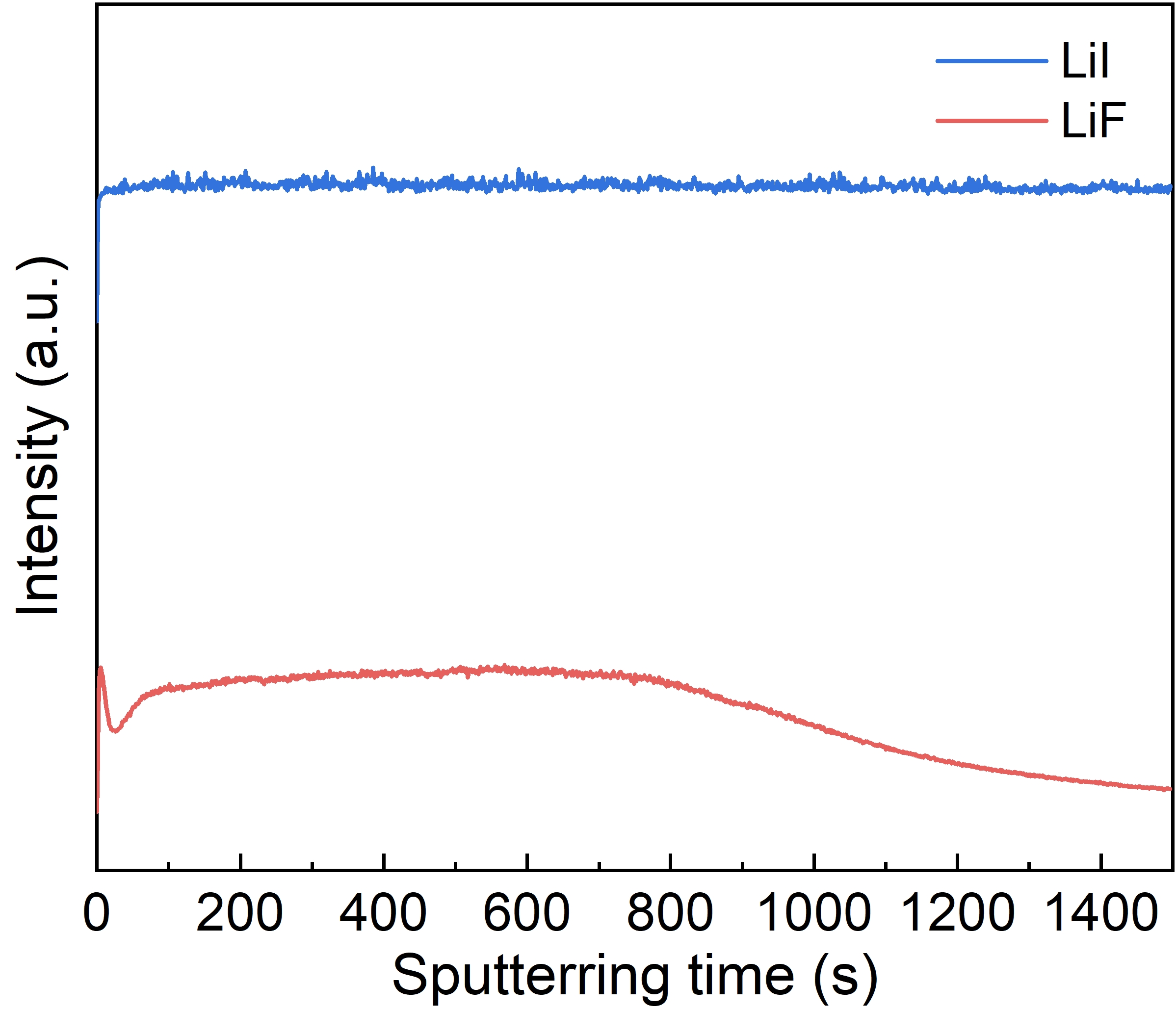


**Figure S39.** Depth profiles of Li anode surface.

**
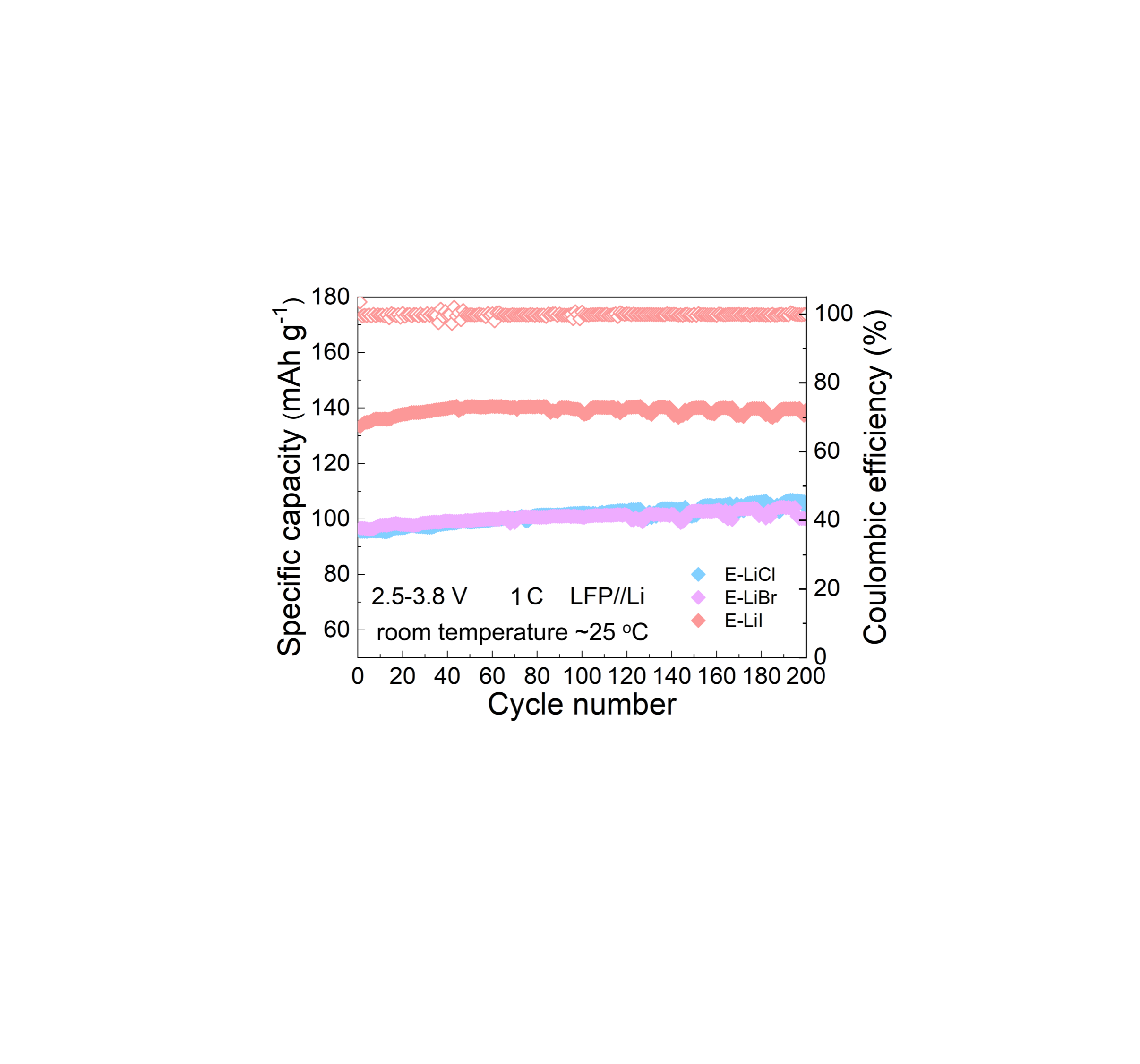
**

**Figure S40.** Cycling performance at 1 C.

**Table S1.** ICP-OES results showing the element content of LiCl@Mil-100, LiBr@Mil-100 and LiI@Mil-100.

|  | Al | Li | Li:Al |
| --- | --- | --- | --- |
| LiCl@Mil-100 | 14.49 wt% | 11.52 wt% | 0.78 |
| LiBr@Mil-100 | 14.29 wt% | 10.58 wt% | 0.74 |
| LiI@Mil-100 | 14.67 wt% | 11.02 wt% | 0.75 |

**Table S2.** Surface energy of Li@LiX at different positions of Li^+^ in LiX.

| LiX (X=Cl, Br or I) | Adsorption site | Surface energy (J m^-2^) |
| --- | --- | --- |
| LiCl | Top1 | 2.68 |
|  | Top2 | 2.86 |
|  | Hollow | 3.05 |
|  | Bridge | 2.94 |
| LiBr | Top1 | 2.41 |
|  | Top2 | 2.75 |
|  | Hollow | 3.12 |
|  | Bridge | 2.79 |
| LiI | Top1 | 2.21 |
|  | Top2 | 2.36 |
|  | Hollow | 2.89 |
|  | Bridge | 2.57 |

In this work, we calculated the adsorption energies of Li⁺ at various sites on LiX surfaces. While our results showed that Li⁺ adsorption energies were lower on LiI compared to LiCl and LiBr, indicating Li⁺ can detach more readily from LiI for migration, the adsorption energy calculation alone does not decisively prove binding affinity to a specific site. Previous studies have shown that preferential binding at a specific site is related to the associated surface energy. A lower surface energy upon binding suggests that Li⁺ adsorbs more favorably at that site, potentially serving as a starting point for migration. To explicitly link the adsorption site geometry to the ion migration pathway, we initially calculated the surface energies resulting from Li⁺ adsorption at the different crystallographic sites considered. The model setup referenced that used for the adsorption energy calculations. The calculated surface energies are summarized in **Table S2**. They reveal that the surface energy is lowest when Li⁺ binds at either Li top sites or halogen top sites, with the Li top sites exhibiting the lowest energy specifically. Consequently, when calculating the ion migration paths and associated energy barriers in this study, we primarily considered migration pathways initiating from a Li⁺ adsorbed at a Li top site or a Halogen top site on the lithium halide surface, migrating towards a neighboring site (e.g., another Li top site or Halogen top site).

**Table S3.** Overpotentials of Li//Li symmetric batteries at different current densities.

|  | Electrolyte | Current density (mA cm^-2^) | | | |
| --- | --- | --- | --- | --- | --- |
|  |  | 0.8 | 2 | 4 | 0.8 |
| Overpotentials  (mV) | PVDF-HFP | 22 | 94 | 80 | 18 |
|  | E-LiCl | 20 | 56 | 68 | 18 |
|  | E-LiBr | 18 | 56 | 62 | 12 |
|  | **E-LiI** | **12** | **40** | **48** | **8** |

**Table S4.** Comparison of E-LiI electrolyte with other reported batteries.

| **Component** | **Cycle performance**  **(mAh g^-1^)** | **Rate performance**  **(mAh g^-1^)** | **Ref.** |
| --- | --- | --- | --- |
| Cu-BDC-10 | 152.5(0.1 C, RT) | 142.1(0.1 C),139.4(0.2 C),  128.3(0.5 C),116.8(1 C),  97(2 C) | 1 |
| PEO-LITFSI/MOF-808 | 140(0.5 C) | - | 2 |
| P-PETEA-MOF | 124.5(0.5 C),100.7(1 C) | - | 3 |
| BMOF@HF/H-ZIF-8 | 135.22(0.5 C) | 147.22(0.1C),142.11(0.2C),  135.15(0.5 C),123.11(1 C),  103.15(2 C) | 4 |
| FAEI | 106.9(1 C) | 167.6(0.1C),116(1C) | 5 |
| PEO/MOFs-NH_2_ | 148.8(0.3 C) | - | 6 |
| MOFs@PP | 100(2 C) | 140(0.1 C),100(2 C) | 7 |
| HKUST-1 | 120(1 C) | 143(0.1 C),132(0.2 C),  125(0.5 C),117(1 C),  97(2 C) | 8 |
| Zn-MOF-74 | 130(0.5 C) | 152(0.1 C),94(1 C) | 9 |
| NO_2_-UIO-66-LI | 126(0.5 C) | 163(0.1 C),161(0.2 C),  146(0.5 C),99(2 C) | 10 |
| H-ZIF-8/HNT | - | 129.22(0.1 C),122.7(0.2C),  116.18(0.5 C),105.31(1 C),  88(2 C) | 11 |
| MOF-BZN-SSE | - | 160(0.1 C),153(0.2 C),  130(0.5 C) | 12 |
| SPE2-PI-ZIF8 | 110(1 C) | 162(0.1 C),134(0.5 C),  117(1 C),97(2 C),  79(3 C),58(5 C) | 13 |
| MOF-688/PVDF | 120.0 (0.2 C) | 160.6(0.1 C),152(0.2 C),  143.4(0.5 C),132.9(0.7 C),  120.7(1 C) | 14 |
| SPE_16-_MOF_10_ | 130(0.1 C) | 125.22(0.1 C),108.34(1 C) | 15 |
| Activated Hollow UiO-66 | 115(1 C) | 150(0.1 C),105(1 C),  92(2 C),80(5 C) | 16 |
| PL10HM | 155(0.1 C) | 160(0.1 C),130(0.2 C),  98(0.3 C),80(0.4 C),  67(0.5 C),48(1 C) | 17 |
| 3D-UIO-66/PAN/PEO | 150(0.2 C) | - | 18 |
| LGZ | 101.2(1 C) | 124.3(0.2C),115.1(0.5),  103.5(1C),90.4(2C),  80.6(3C),73.5(4C) | 19 |
| PUIE | 140.2(0.1C) | 137.9(0.1C),134.6(0.2C),  131.2(0.5C),100.1(1C) | 20 |
| **E-LiI** | **120(2 C)** | **140(0.2 C),135(0.5 C),**  **120(1 C),115(2 C),**  **80(5 C)** | **This work** |

**References**

1. X Wang, S Jin, L Shi, N Zhang, J Guo, D Zhang, and Z Liu ACS Appl. Mater. Interfaces 2024, 16, 33954−33962
2. Z Hong, P Li, Q Zou, L Gu, J Wang, L Deng, C Wang, Y Zhang, M Li, J Chen, R Si, and C Yang ACS Appl. Energy Mater. 2024, 7, 11967−11976
3. J. Zhou, X. Wang, J. Fu, L. Chen, X. Wei, R. Jia, L. Shi Small 2024, 20, 2309317
4. S Li, Yini Chen, Xiaolong Leng, Mingdai Yang, Waqas Ul Arifeen, Tae Jo Ko Chemical Engineering Journal 500 (2024) 157209
5. L Liu, L Zhu, Y Wang, X Guan, Z Zhang, H Li, F Wang, H Zhang, Z Zhang, Z Yang, and T Ma Angew. Chem. Int. Ed. 2025, 64, e202420001
6. L Xu, X Xiao, H Tu, F Zhu, J Wang, H Liu, W Huang, W Deng, H Hou, T Liu, X Ji, K Amine, and G Zou Adv. Mater. 2023, 35, 2303193
7. Z Hao, Y Wu, Q Zhao, Jg Tang, Q Zhang, X Ke, J Liu, Y Jin, and H Wang, Adv. Funct. Mater. 2021, 31, 2102938
8. H Liu, H Pan, Mi Yan, X Zhang, and Y Jiang Adv. Mater. 2023, 35, 2300888
9. P Dong, X Zhang, W Hiscox, J Liu, J Zamora, X Li, M Su, Q Zhang, X Guo, J McCloy, and M Song Adv. Mater. 2023, 35, 2211841
10. Y Zhou, J Chen, J Sun, and T Zhao *Nano Lett.* 2024, 24, 2033−2040
11. F Tao, X Wang, S Jin, L Tian, Z Liu, X Kang, and Z Liu Adv. Mater. 2023, 35, 2300687
12. Y Ouyang, W Gong, Q Zhang, J Wang, S Guo, Y Xiao, D Li, C Wang, X Sun, C Wang, and S Huang Adv. Mater. 2023, 35, 2304685
13. G. X. Wang, Dr. P. G. He, Prof. L.-Z. Fan Adv. Funct. Mater. 2021, 31, 2007198
14. D. Wang, H. Xie, Q. Liu, K. Mu, Z. Song, W. Xu, L. Tian, C. Zhu, J. Xu, Angew. Chem., Int. Ed 552023, 62, e202302767
15. H Luo1, D Wu, J Liang, H Zou, J Zhuang, Z Chen, H Cheng, Electrochimica Acta 513 (2025) 145543
16. Z. Liu, W. Chen, F. Zhang, F. Wu, R. Chen, L. Li, Small, **2023**, *19*, e2206655.
17. C Sun, A Yusuf, S Li, X Qia, Y Ma, D Wang, Chemical Engineering Journal 414 (2021) 128702
18. Z Li, S Wang, J Shi, Y Liu, S Zheng, H Zou, Y Chen, W Kuang, K Ding, L Chen, Y Lan, Y Cai, Q Zheng, Energy Storage Materials 47 (2022) 262–270
19. G. Jiang, C. Qu, F. Xu, E. Zhang, Q. Lu, X. Cai, S. Hausdorf, H. Wang and S. Kaskel, *Adv. Funct. Mater.*, **2021**, *31*, 2104300.
20. Z. Zhang, Y. Huang, H. Gao, J. Hang, C. Li, P. Liu, J. Membr. Sci. 2020, 598, 117800.
